# Supplementary material for: Discovery of a Drug-like, Natural Product-Inspired DCAF11 Ligand Chemotype
Source: Nat Commun. 2023 Nov 30;14:7908. doi: 10.1038/s41467-023-43657-6 (PMC10689823; doi:10.1038/s41467-023-43657-6)
Supplement: Supplementary file 1 — Supplementary Information [file 41467_2023_43657_MOESM1_ESM.pdf]

Discovery of a Drug-like, Natural Product-Inspired, DCAF11 Ligand Chemotype

Authors

Gang Xue,[1,4] Jianing Xie,[1,4] Matthias Hinterndorfer,[2] Marko Cigler,[2] Lara Dötsch,[1,3] Hana Imrichova,[2] Philipp Lampe,[1] Xiufen Cheng,[1] Soheila Rezaei Adariani,[1] Georg E. Winter*,[2] and Herbert Waldmann*[1,3]

Affiliations

[1] Department of Chemical Biology, Max Planck Institute of Molecular Physiology, Dortmund, Germany.

[2] CeMM Research Center for Molecular Medicine of the Austrian Academy of Sciences, Vienna, Austria.

[3] Technical University Dortmund, Faculty of Chemistry and Chemical Biology, Dortmund, Germany.

[4] These authors contributed equally: Gang Xue, Jianing Xie.

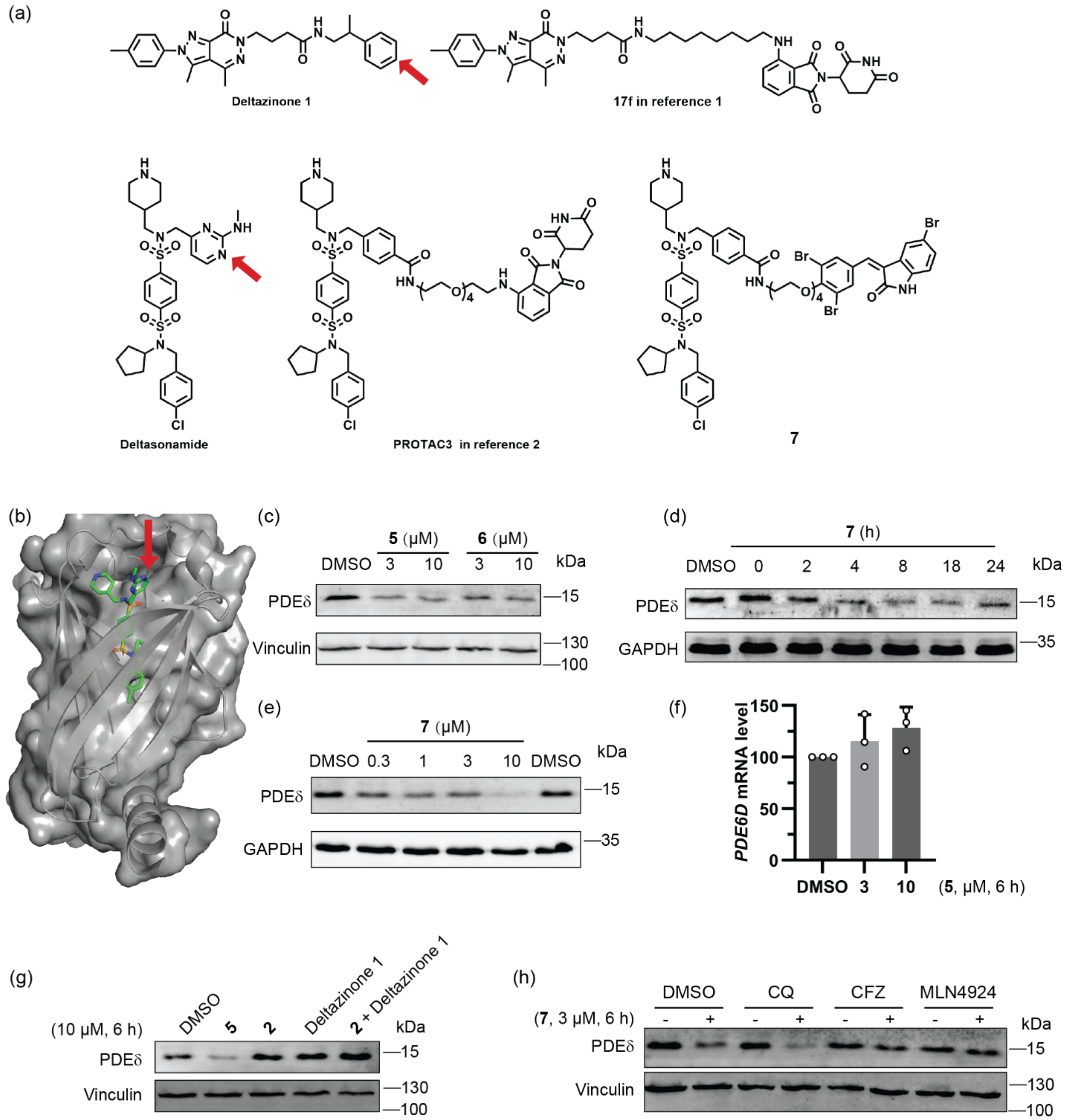

Supplementary Fig. 1 | Degradation of PDEδ induced by bifunctional arylidene-indolinones 5, 6 and 7. **a**, Structures of PDEδ ligands, PROTACs (**17f**[1], and PROTAC3[2]), and compound **7**. **17f** is based on deltazinone and PROTAC3 is based on the deltasonamide. The arrows in the figure indicate the points to connect the linker. **b,** Crystal structure of PDEδ in complex with deltasonamide (PDB: 5ML3). The arrow in the figure indicates the solvent exposed region. **c**, PDEδ levels in Jurkat cells treated with compounds **5** and **6** (6 h). Representative result of n = 3 is shown. **d**, Time-dependent degradation of PDEδ in Jurkat cells treated with compound **7** (3 μM). Representative results of two experiments is shown. **e,** Dose-dependent degradation of PDEδ in Jurkat cells treated with compound **7** (18 h). Representative result of n = 3 is shown. **f**, *PDEδ* mRNA levels in Jurkat cells treated with compound **5** for 6 h. Data are mean values ± SD (n = 3

biological replicates). **g**, PDEδ protein levels in Jurkat cells treated with compounds **5**, **2**, Deltazinone or **2** plus Deltazinone 1 (10 µM, 6 h). Representative result of n = 3 is shown. **h**, PDEδ levels in Jurkat cells pretreated with autophagy inhibitor CQ (50 µM), proteasome inhibitor CFZ (0.2 µM) and MLN4924 (1 µM) for 2 h, followed by treatment with 3 µM compound **7** for another 6 h. Representative result of n = 3 is shown.

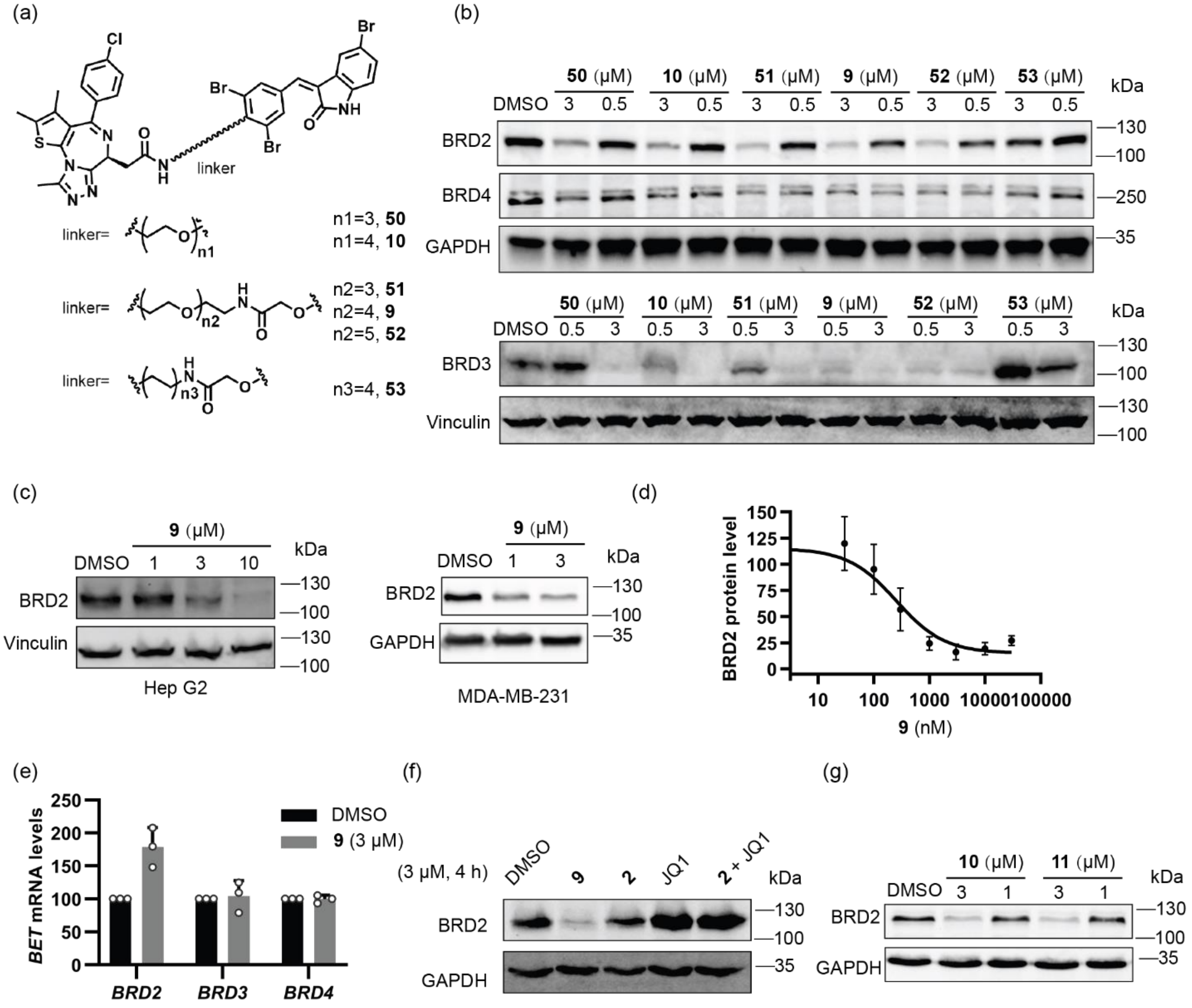

Supplementary Fig. 2 | Degradation of BET proteins and BTK induced by bifunctional degraders. **a**, Structures of the bifunctional degraders for BET proteins (**9**, **10** and **50**-**53**). **b**, BRD2, 3, and 4 proteins level in Jurkat cells treated with compounds **9**, **10** and **50**-**53** (0.5 µM and 3 µM, 6 h). One representative of two experiments is shown. **c**, BRD2 protein level in Hep G2 cells and MDA-MB-231 cells treated with compound **9** (4 h) at the indicated concentration. One representative of two experiments is shown. **d**, Quantification of the relative BRD2 protein content in Jurkat cells treated with **9** (6 h). Data were normalized to the DMSO control. Data are mean values ± SD. (n = 4 biological replicates). **e**, *BET* mRNA levels in Jurkat cells treated with **9** (3 µM, 4 h). Data are mean values ± SD (n = 3 biological replicates). **f**, BRD2 protein level in Jurkat

cells treated with **9, 2,** JQ1 or **2** plus JQ1 (3 μM, 4 h). Representative result of n = 3 is shown. **g**, BRD2 protein level in Jurkat cells treated with **10** and **11** for 6 h. Representative result of n = 3 is shown.

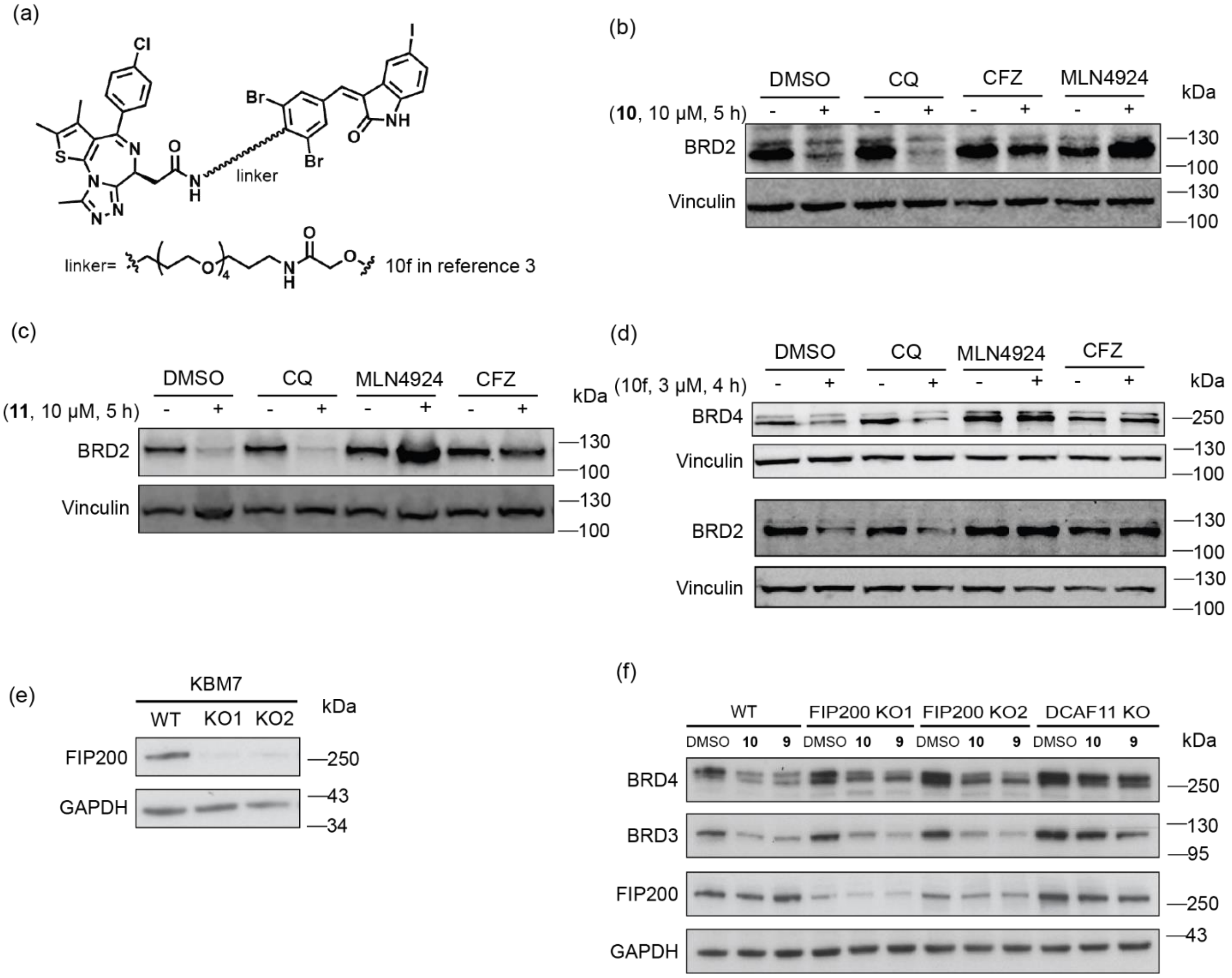

Supplementary Fig. 3 | Degradation mechanism of BET proteins induced by bifunctional degraders. a, Structure of **10f** in reference 6[3]. **b**, **c**, BRD2 protein level in Jurkat cells pretreated with autophagy inhibitor CQ (50 μM), proteasome inhibitor CFZ (1 μM) and neddylation inhibitor MLN4924 (1 μM) for 40 min, followed by treatment with 10 μM of compound **10** (**b**) and **11** (**c**) for another 5 h. Representative result of n = 3 is shown. **d**, BRD2 and BRD4 protein level in MDA-MB-231 cells pretreated with autophagy inhibitor CQ (50 μM), neddylation inhibitor MLN4924 (1 μM) or proteasome inhibitors Carfilzomib (CFZ, 1 μM) for 2 h, followed by treatment with 3 μM of compound **10f** for another 4 h. Representative result of n = 3 is shown. **e.** FIP200 level in wild type (WT) KBM7 cells and FIP200 knockout (KO1 and KO2) KBM7 cells. Representative result of n = 2. **f.** BRD3 and BRD4 protein levels in wild type (WT) KBM7 cells, two independent FIP200 knockouts and DCAF11 knockout KBM7 cells treated with compound **9** (1 μM, 6 h) and **10** (10 μM, 6 h). Representative results (n = 2).

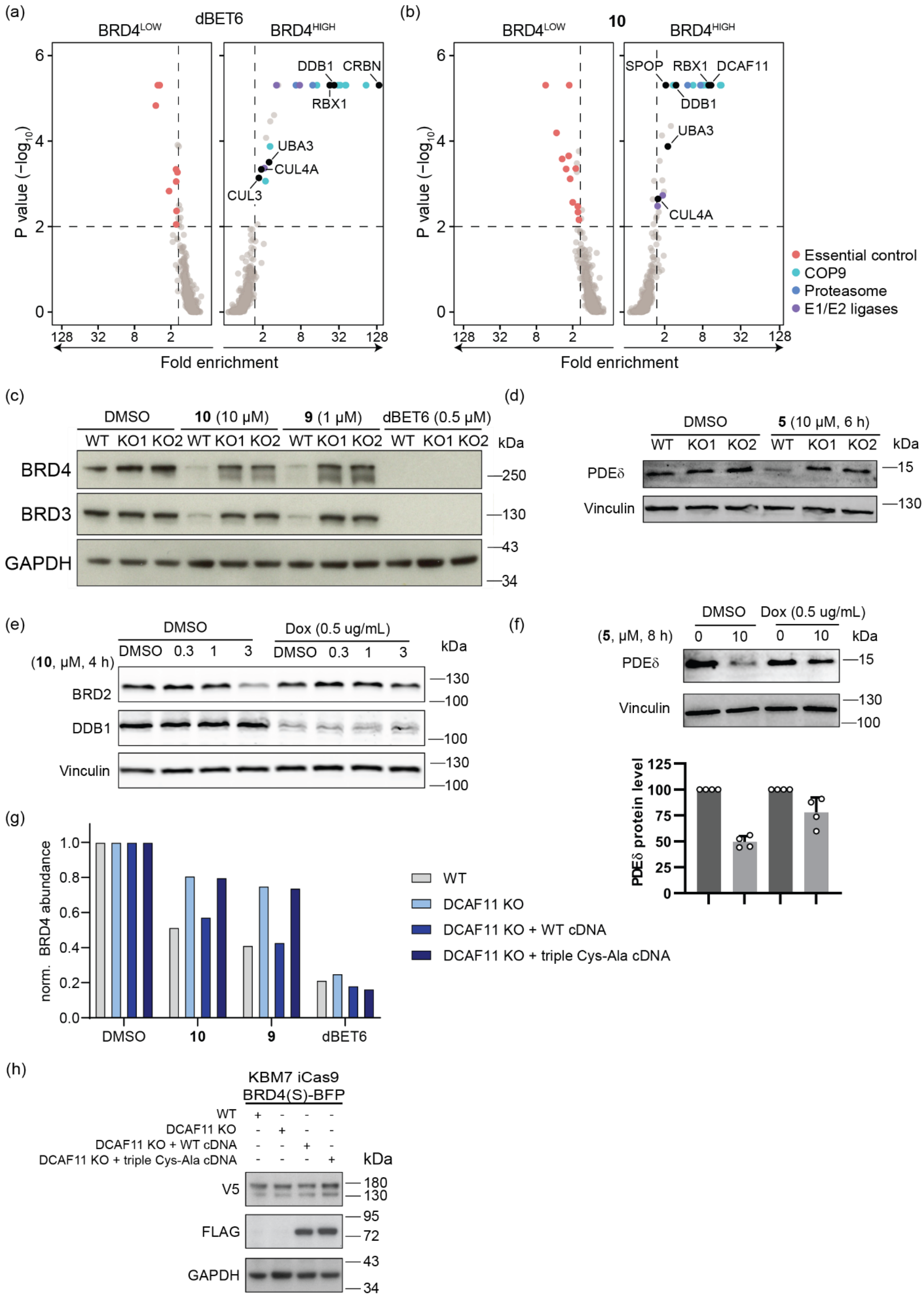

(a)
dBET6
BRD4LOW
BRD4HIGH
DDB1
CRBN
RBX1
UBA3
CUL4A
CUL3
P value (−log10)
Fold enrichment
(b)
10
SPOP
RBX1
DCAF11
DDB1
UBA3
CUL4A
Essential control
COP9
Proteasome
E1/E2 ligases
(c)
DMSO
10 (10 μM)
9 (1 μM)
dBET6 (0.5 μM)
WT KO1 KO2
kDa
BRD4
BRD3
GAPDH
(d)
DMSO
5 (10 μM, 6 h)
PDEδ
Vinculin
(e)
Dox (0.5 ug/mL)
(10, μM, 4 h)
BRD2
DDB1
Vinculin
(f)
(5, μM, 8 h)
PDEδ protein level
(g)
norm. BRD4 abundance
WT
DCAF11 KO
DCAF11 KO + WT cDNA
DCAF11 KO + triple Cys-Ala cDNA
(h)
KBM7 iCas9
BRD4(S)-BFP
V5
FLAG
GAPDH

Supplementary Fig. 4 | Degradation of BET proteins by compound 9 or 10, and of PDEδ by compound 5 is based on CRL^{DCAF11} complex. a, **b**, FACS-based CRISPR screens for regulators of BRD4 degradation induced by dBET6 (a) and **10** (b). Average gene-level fold-changes and p-values of BRD4^{HIGH} and BRD4^{LOW} cell populations compared to BRD4^{MID} fraction were calculated using MAGeCK. Essential control genes (BRD4^{LOW}) and 20S proteasome subunits, COP9 signalosome subunits and E1 or E2 ubiquitin ligases (BRD4^{HIGH}) inside the scoring window (p-value < 0.01, fold-change > 1.5) are labelled. **c**, BRD3 and BRD 4 level in wild type, DCAF11 knockout 1 (KO1) and DCAF11 knockout 2 (KO2) KBM7 cells treated with **9** (1 µM, 6 h), **10** (10 µM, 6 h) and dBET6 (0.5 µM, 6 h). Representative result of n = 3. **d**, PDEδ protein level in wild type, DCAF11 knockout 1 (KO1) and DCAF11 knockout 2 (KO2) KBM7 cells treated with compound **5** (10 µM, 6 h). Representative result of n = 3. **e**, BRD2 and DDB1 level in Dox-inducible DDB1 knockout KBM7 cells pretreated with 0.5 ug/mL Dox for 3 days, followed by treated with **10** for another 4 h. Representative result of n = 2. **f**, PDEδ level in Dox-inducible DDB1 knockout KBM7 cells pretreated with 0.5 ug/mL Dox for 3 days followed by incubation with **5** (10 µM) for another 8 h. Quantification of the relative PDEδ content is shown in the bar graph. Data were normalized to the DMSO control. Data were mean values ± SD (n=4 biological replicates). **g**, DCAF11 knockout/rescue. KBM7 iCas9 BRD4(S)-BFP reporter cells were transduced with lentivirus expressing DCAF11-targeting sgRNA as well as two different sgRNA-resistant DCAF11 expression vectors (WT DCAF11 or triple Cys-Ala mutant). After 4 days of dox-induced Cas9 expression, cells were treated with DMSO, **9** (1 µM), **10** (5 µM) or dBET6 (0.5 µM) for 6 h and BRD4-BFP levels analyzed via flow cytometry. Representative result of n = 2. **h,** Expression levels of V5-tagged BRD4(S)-BFP reporter and FLAG-tagged wild type (WT) and triple Cys-Ala mutant DCAF11 in KBM7 iCas9 cells. Representative result of n = 2.

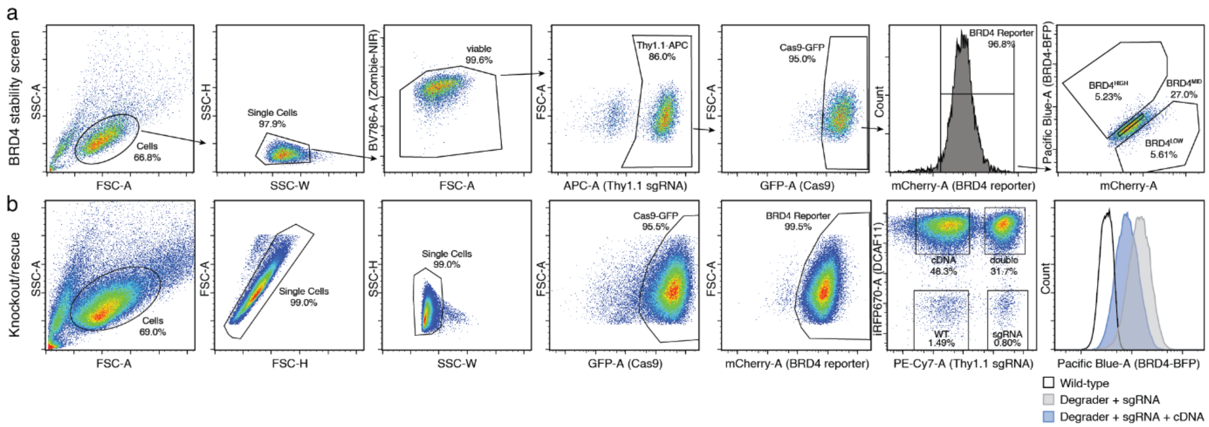

Supplementary Figure 5 | Gating strategy for flow cytometric analysis and cell sorting. Representative scatter plots of hierarchical flow cytometry gating strategies. **a**, FACS-based BRD4 stability CRISPR screens, as in Fig. 3a-c and Supplementary Fig. 4a, b. b, Knockout/rescue screen validation assay as in Supplementary Fig. 4 g. In all analyses, forward scatter area vs. side scatter area plot was used to separate viable cells from debris and dead cells. Forward scatter height vs. forward scatter area and/or side scatter width vs. side scatter height plots were used to separate single cells from aggregates. For the sorting of fixed cells in CRISPR BRD4 protein stability screens (a), dead cells were excluded based on Zombia-NIR staining (BV786-A) vs FSC-A and sgRNA library (Thy1.1-APC-A), iCas9 (GFP-A) and reporter (mCherry-A) triple positive cells were sorted into BRD4LOW, BRD4HIGH, and BRD4MID populations based on BRD4-BFP vs mCherry scatter plots. These gates were dynamically adjusted to keep the percentage at

5-10% for BRD4^{HIGH} and BRD4^{LOW} and 25-30% for BRD4^{MID} populations. For knockout/rescue experiments (b), sgRNA (Thy1.1 PE-Cy7) single positive cells were separated from sgRNA and DCAF11 cDNA (iRFP670) double positive BRD4-BFP reporter cells and the BFP/mCherry ratios of these two populations were compared.

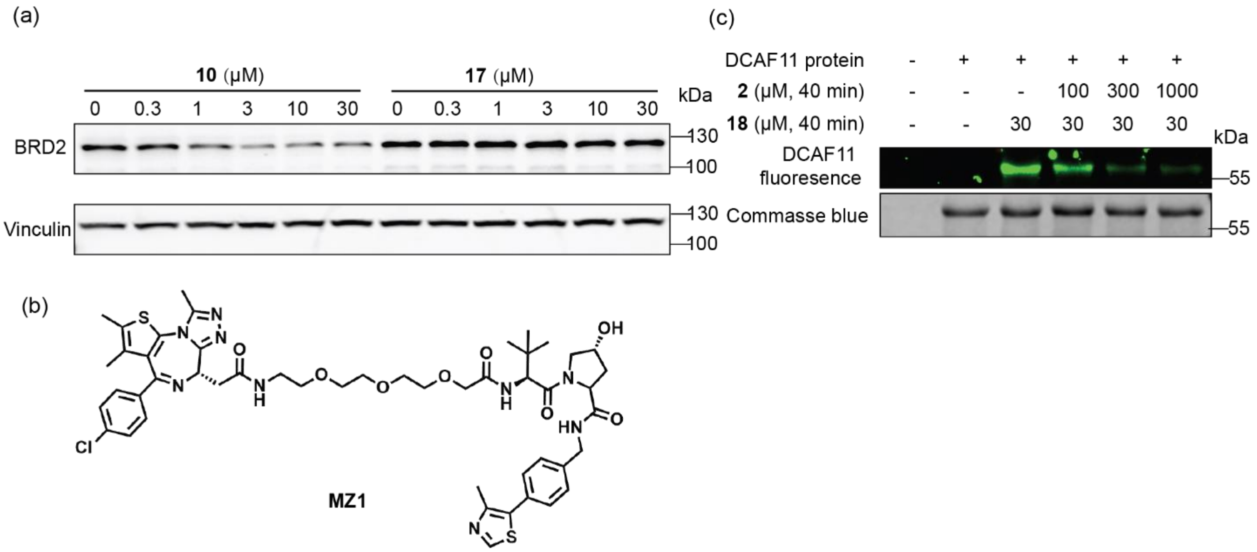

Supplementary Fig. 6 | Compounds 10 and 2 covalently bind to DCAF11. a, BRD2 in KBM7 cells treated with compound **10** and **17** for 4 h. Representative result of two experiments is shown. **b**, Structure of MZ1. MZ1 is a non-covalent BRD2, 3- and 4 PROTAC based on the VHL E3 ligase. **c**, Competition of fluorescence labeling of DCFA11. Purified DCAF11 protein was pretreated with compound 2 for 40 min, and then treated with **18** for another 40 min, followed by analysis of in-gel fluorescence.

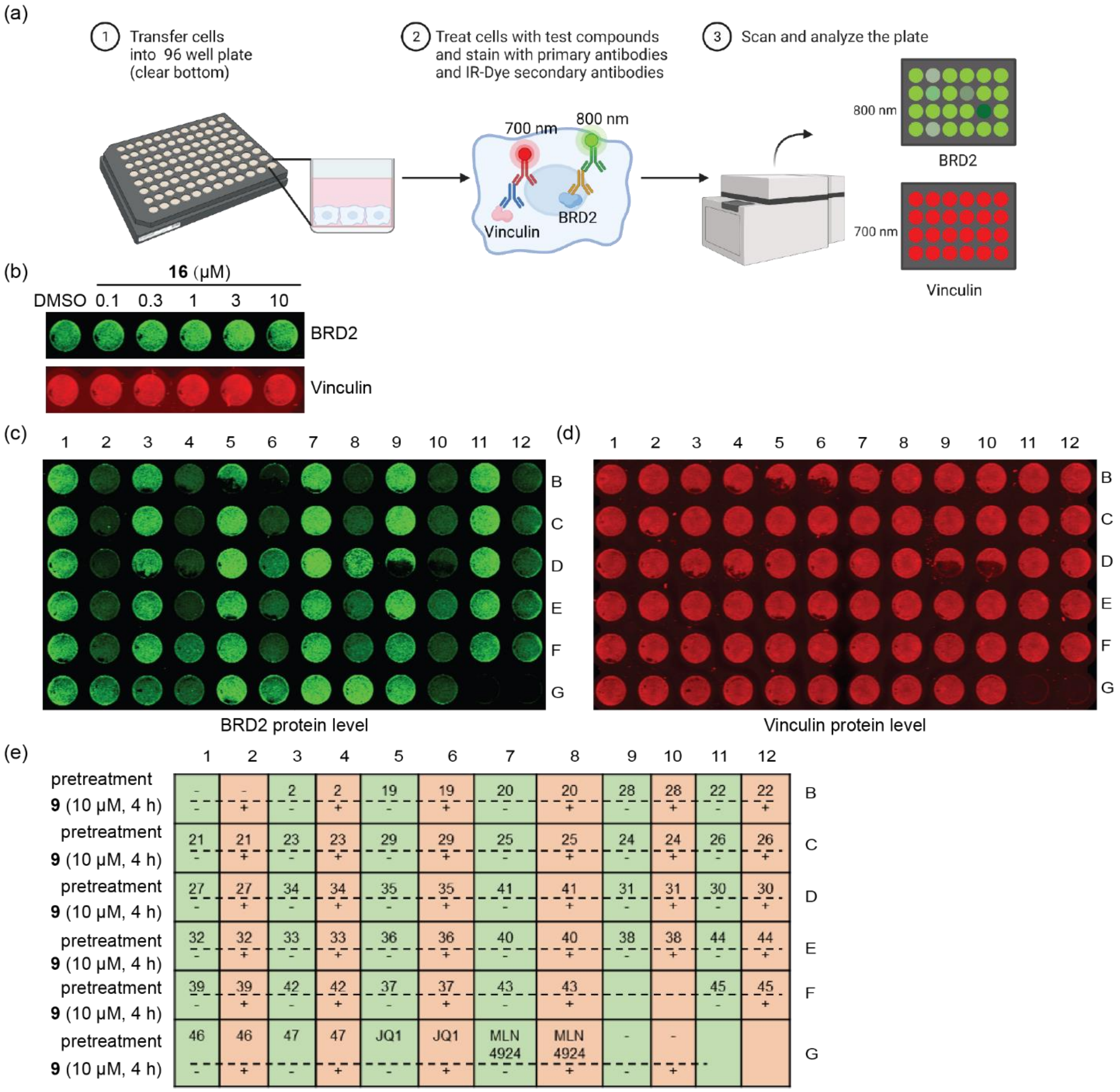

Supplementary Fig. 7 | Results of In-Cell Western. **a**, In-Cell Western assay[4] (created with BioRender.com). Hep G2 cells were seeded into 96 well plates and treated with compounds. Target proteins were stained with the primary antibodies and IR-Dye secondary antibodies, followed by scanning and analyzing the plates. **b**, In-Cell Western for BRD2 levels in Hep G2 cells treated with compound **16** for 4 h. Representative results (n = 3). **c**, **d**, **e**, Results of In-Cell Western in HepG2 cells. BRD2 protein level (c) and Vinculin protein level (d) in Hep G2 cells pretreated with compound **2** or **19**-**47** (structure shown in the Supporting Table S3) at 30 μM for 1 h, and then treated with **9** (10 μM) for another 4 h as the plate layout (e) in each well of 96 well plate, followed by scanning the plate with 700 nm and 800 nm.

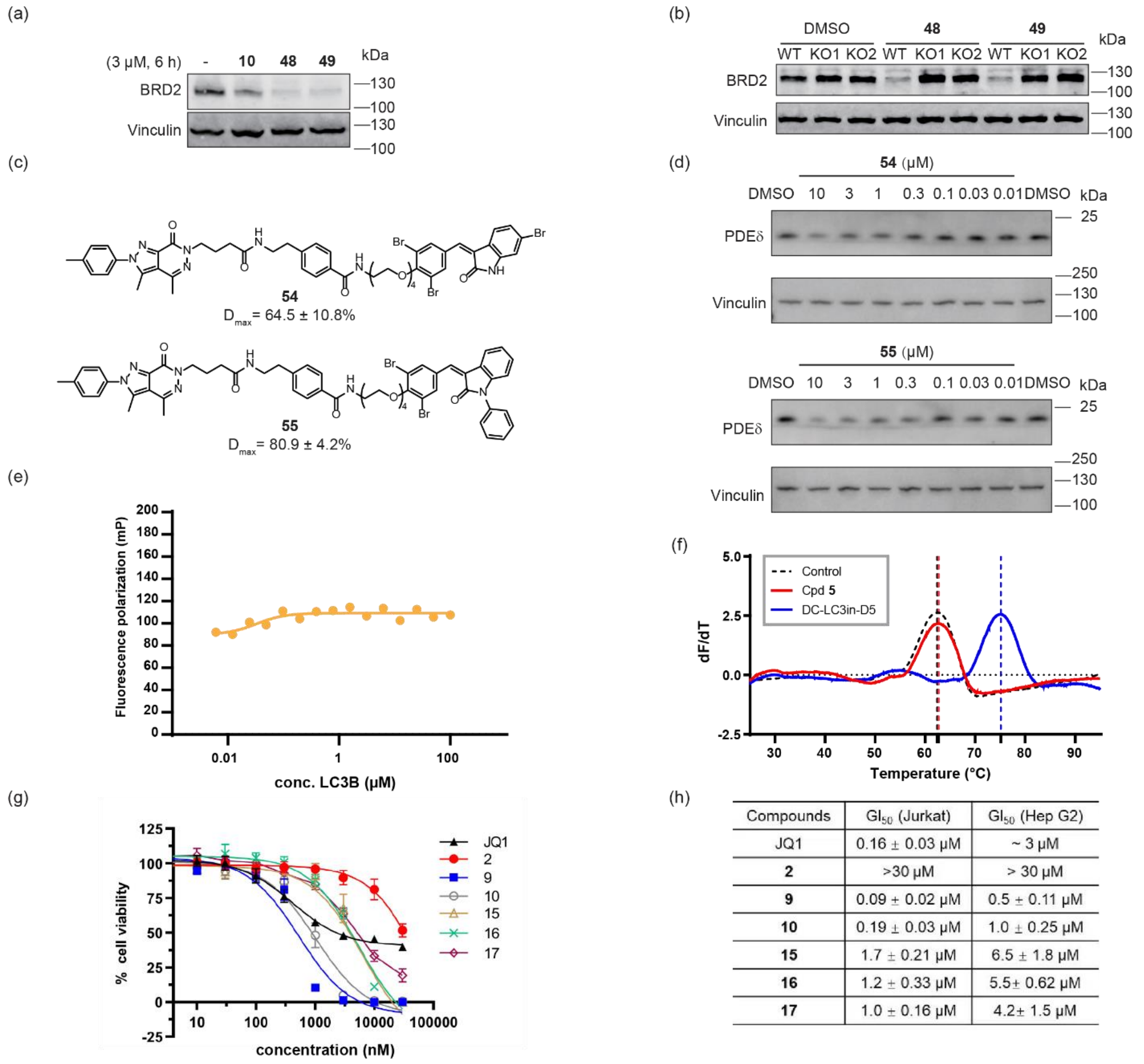

Compounds	GI_{50} (Jurkat)	GI_{50} (Hep G2)
JQ1	0.16 ± 0.03 μM	~ 3 μM
**2**	>30 μM	> 30 μM
**9**	0.09 ± 0.02 μM	0.5 ± 0.11 μM
**10**	0.19 ± 0.03 μM	1.0 ± 0.25 μM
**15**	1.7 ± 0.21 μM	6.5 ± 1.8 μM
**16**	1.2 ± 0.33 μM	5.5± 0.62 μM
**17**	1.0 ± 0.16 μM	4.2± 1.5 μM

Supplementary Fig. 8 | Results of the optimization of degradation activity and the cell viability assay. **a**, BRD2 in Jurkat cells treated with compound **10**, **48**, and **49** at 3 μM for 6 h. Representative result of n = 4 is shown. **b**, BRD2 levels in WT and DCAF11 knockout (KO1 and KO2) KBM7 cells treated with compound **48** and **49** at 3 μM, for 6 h. Data are representative of n = 3. **c**, Structures of bifunctional degraders **50** and **51** derived from the structure-activity relationship (SAR) shown in Fig. 4. **d**, Dose-dependent degradation of PDEδ in Jurkat cells treated with compound **54** and **55** for 6 h. Representative result of n = 3 is shown. **e**. Potential binding of probe **18** to LC3B was assessed by means of fluorescence polarization. Representative result of n = 2. **f.** Potential binding of compound **5** to LC3B as determined using DSF and Glo-Melt. Compound DC-LC3in-D5 was used a control. Melting temperature shift (Δ*T*m) DC-LC3in-D5 = 13.0 °C. Representative result of n = 2. **g**, Cell viability assay. Hep G2 cells were treated with compounds for 3 days, and cell viability was determined by means of the CellTiter-Glo reagent.

Data are mean values ± SD (n = 3 biological replicates). **h**, GI_{50} data in Jurkat cells and Hep G2 cells treated with compounds JQ1, **2**, **9**,**10** and **15-17**. Data are mean values ± SD (n = 3 biological replicates).

Supplementary Tables

Supplementary Table 1. Activity of compounds **2**, **19**-**47**, MLN4924 and JQ1 by qualitative ICW assay

Cmpd	Structure	Value (Mean ± SD)	Cmpd	Structure	Value (Mean ± SD)
**2**	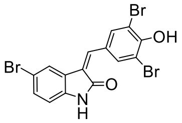	44.85 ± 4.23	**34**	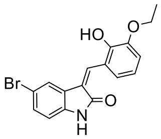	38.78 ± 7.19
**19**	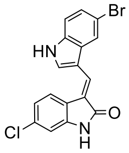	22.45 ± 2.48	**35**	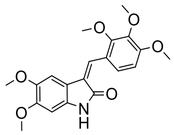	43.80 ± 10.34
**20**	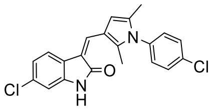	23.05 ± 3.61	**36**	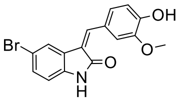	44.21 ± 2.82
**21**	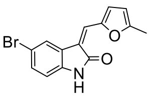	27.96 ± 5.89	**37**	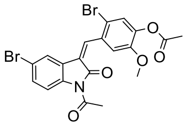	46.27 ± 6.95
**22**	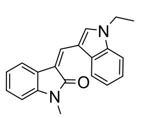	28.31 ± 1.06	**38**	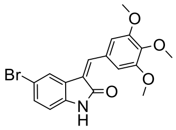	46.38 ± 2.20
**23**	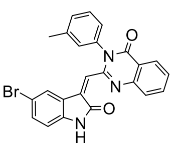	28.93 ± 2.95	**39**	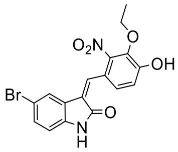	46.41 ± 4.18
**24**	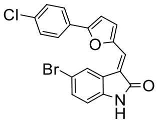	32.30 ± 3.56	**40**	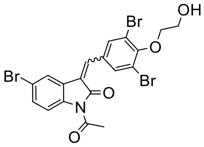	49.83 ± 3.84
**25**	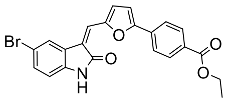	32.83 ± 1.96	**41**	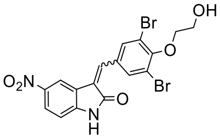	51.57 ± 11.55
**26**	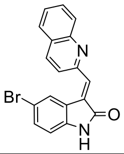	38.97 ± 1.11	**42**	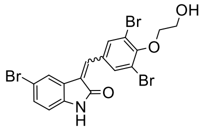	55.54 ± 6.61

Cmpd	Structure	Value (Mean ± SD)	Cmpd	Structure	Value (Mean ± SD)
**27**	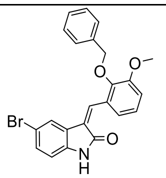	25.66 ± 3.60	**43**	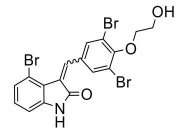	55.89 ± 8.40
**28**	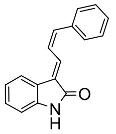	27.19 ± 3.29	**44**	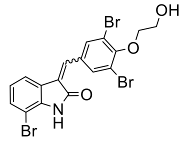	57.04 ± 9.16
**29**	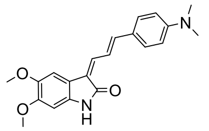	31.86 ± 2.35	**45**	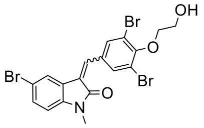	67.69 ± 4.30
**30**	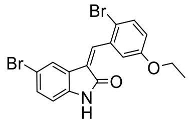	36.18 ± 4.38	**46**	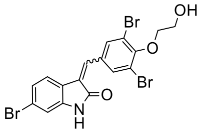	70.23 ± 9.63
**31**	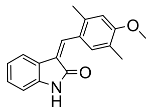	36.20 ± 1.35	**47**	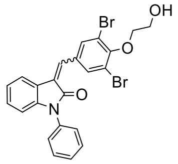	72.76 ± 4.44
**32**	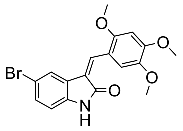	38.22 ± 2.33	**JQ1**	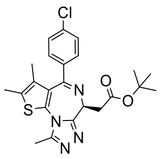	67.81 ± 1.42
**33**	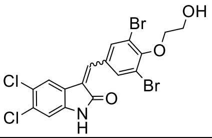	38.55 ± 3.55	**MLN 4924**	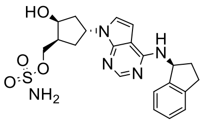	112.98 ± 3.16

Value: Relative BRD2 levels remaining in Hep G2 cells after treatment with compounds (**2**, **19-47**, JQ1 and MLN4924) followed by treatment with **9**, with DMSO control for normalization.

Supplementary Table 2. SIRT activity in presence of 3 µM compound **42**.

**Protein**	**Activity [%]** (N=1)	**Activity [%]** (N=2)	**Mean activity [%]**
**SIRT1**	93	97	95
**SIRT2**	104	111	107.5
**SIRT3**	67	67	67
**SIRT5**	95	98	96.5
**SIRT6**	87	90	88.5

Chemical synthesis

General information

Unless otherwise noted, all commercially available compounds and (anhydrous) solvents were purchased from Sigma Aldrich, TCI Europe, Alfa Aesar, or Acros Organic, and used without further purification. Analytical thin-layer chromatography (TLC) was performed on Merck silica gel aluminum plates with F-254 indicator. Compounds were visualized by irradiation with UV light or potassium permanganate staining followed by heating with a heat gun. Column chromatography was performed using silica gel Merck 60 (particle size 0.040-0.063 mm). Solvent mixtures are understood as volume/volume.

Preparative HPLC-MS: Separations were carried out using a preparative mass-directed HPLC (Agilent Series, 1100/LC/MSD VL, Agilent Series) with a reversed-phase C18 column (flow 20.0 mL/min, solvent A: 0.1% TFA in water, solvent B: 0.1% TFA in acetonitrile).

^{1}H NMR, ^{13}C NMR spectra were recorded at room temperature on a Bruker DRX400 (400 MHz), INOVA500 (500 MHz), INOVA 600 or INOVA 700 and referenced to the residual deuterated solvent signals. All reported NMR values are given in parts per million (ppm). Multiplicities are indicated s (singlet), d (doublet), t (triplet), q (quartet), m (multiplet); coupling constants (*J*) are given in Hertz (Hz).

High resolution mass spectra were recorded on a *LTQ Orbitrap* mass spectrometer coupled to an Accela HPLC-System (HPLC column: Hypersyl GOLD, 50 mm x 1 mm, particle size 1.9 µm, ionization method: electron spray ionization).

5-bromo-3-(3,5-dibromo-4-hydroxybenzylidene)indolin-2-one (2)

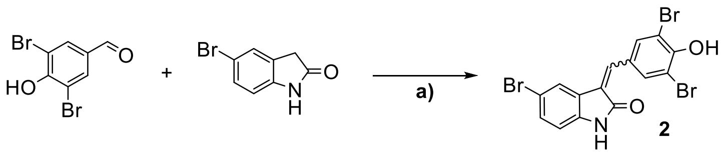

Supplementary Fig. 9 | Synthesis of compound 2. **a)** piperidine, EtOH, 90 ºC, 12 h.

a): Piperidine (5 µL, 0.05 mmol, 0.1 eq.) was added into a solution of 5-bromooxindole (0.106 g, 0.5 mmol, 1 eq.) and 3,5-dibromo-4-hydroxybenzaldehyde (0.154 g, 0.55 mmol, 1 eq.) in EtOH (6 mL) and the solution was stirred for 12 h at 90 ºC. The solution was cooled to room temperature, filtered, and the filter cake was washed with cold MeOH (5 mL) for two times to provide **2** as a yellow solid (0.109 g, 46%).

^{1}**H NMR** (500 MHz, DMSO-d_6) δ 10.78 – 10.70 (s, 1H), 8.79 (s, 1.28H), 7.93 (s, 0.66H), 7.87 (d, *J* = 2.0 Hz, 0.64H), 7.79 (s, 0.64H), 7.62 (d, *J* = 1.9 Hz, 0.36H), 7.55 (s, 0.36H), 7.42 (dd, *J* = 8.4, 2.0 Hz, 0.36H), 7.34 (dd, *J* = 8.2, 2.0 Hz, 0.64H), 6.85 (d, *J* = 8.3 Hz, 0.36H), 6.78 (d, *J* = 8.2 Hz, 0.64H).

^{13}**C NMR** (126 MHz, DMSO-d_6) δ 168.07, 166.97, 142.01, 139.33, 136.56, 136.24, 135.16, 133.45, 132.24, 130.54, 127.52, 124.41, 123.02, 122.10, 113.02, 112.58, 112.04, 111.58, 111.19.

HRMS calculated for $C_{15}H_9Br_2{}^{81}BrNO_2$ $[M + H]^+$: 473.8158, found 473.8157.

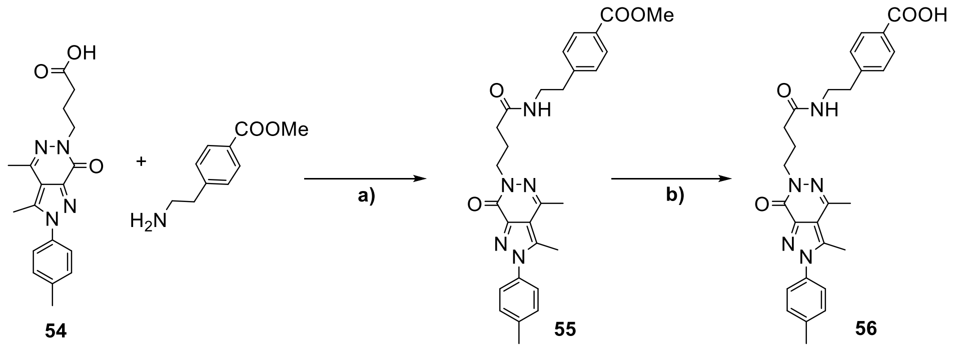

Supplementary Fig. 10 | Synthesis of compound 56. **a)** HATU, DIPEA, DMF, rt, 12 h.; **b)** LiOH, MeOH, H_2O, rt, 10 h.

Compound **54** was synthesized according to the literature[5].

a): DIEAP (1.046 mL, 6 mmol, 3 eq.) was added into the mixture of compound **54** (0.6808 g, 2 mmol, 1 eq.) and HATU (0.912 g, 2.4 mmol, 1.2 eq.) in DMF (6 mL) and stirred for 10 min. After that, methyl 4-(2-amino-ethyl)-benzoate (0.43 g, 2.4 mmol, 1.2 eq.) was

added into the resulting solution and stirred at room temperature for 4 h. The reaction mixture was diluted with EtOAc, washed with water and brine, dried over Na_2SO_4. After removing the solvent under reduced pressure, the product **55** (0.5919 g, 59%) could be obtained by flash chromatography (DCM : MeOH = 40 : 1) as a light yellow solid.

methyl 4-(2-(4-(3,4-dimethyl-7-oxo-2-(p-tolyl)-2,7-dihydro-6H-pyrazolo[3,4-d]pyridazin-6-yl)butanamido)ethyl)benzoate (55)

^{1}H NMR (700 MHz, $CDCl_3$) δ 7.93 (d, *J* = 8.3 Hz, 2H), 7.37 – 7.32 (m, 4H), 7.30 (d, *J* = 8.2 Hz, 2H), 4.13 (t, *J* = 6.0 Hz, 2H), 3.88 (s, 3H), 3.55 (q, *J* = 7.0 Hz, 2H), 2.96 (t, *J* = 7.5 Hz, 2H), 2.63 (s, 3H), 2.55 (s, 3H), 2.45 (s, 3H), 2.19 (dd, *J* = 7.9, 5.2 Hz, 2H), 2.12 (q, *J* = 5.9 Hz, 2H).

^{13}C NMR (176 MHz, $CDCl_3$) δ 172.97, 167.14, 157.14, 144.88, 141.98, 141.84, 140.03, 136.57, 135.95, 130.00, 129.81, 128.95, 128.26, 125.83, 117.84, 52.04, 48.61, 40.50, 35.77, 33.46, 25.70, 21.33, 19.97, 12.39.

MS calculated for $C_{28}H_{32}N_5O_4$ $[M + H]^+$: 502.2, found 502.2.

b): Compound **55** (0.5016 g, 1 mmol, 1 eq.) was added to the solution of LiOH (72 mg, 3 mmol, 3 eq.) in MeOH/H_2O (v/v = 5/1, 4 mL). After stirring for 10 h, the reaction mixture was diluted with EtOAc, and acidified with 1 M HCl. The aqueous layer was extracted with EtOAc, and the combined organic layer was dried over anhydrous $MgSO_4$ and concentrated under vacuum. The obtained product **56** (40 mg, 82%) can be used for next step without further purification.

4-(2-(4-(3,4-dimethyl-7-oxo-2-(p-tolyl)-2,7-dihydro-6H-pyrazolo[3,4-d]pyridazin-6-yl)butanamido)ethyl)benzoic acid (56)

^{1}H NMR (400 MHz, DMSO-*d*$_6$) δ 12.71 (s, 1H), 7.84 (t, *J* = 5.6 Hz, 1H), 7.80 – 7.73 (m, 2H), 7.43 – 7.33 (m, 4H), 7.24 (d, *J* = 8.3 Hz, 2H), 3.94 (t, *J* = 7.0 Hz, 2H), 3.21 (q, *J* = 7.1 Hz, 2H), 2.69 (t, *J* = 7.2 Hz, 2H), 2.51 (s, 3H), 2.44 (s, 3H), 2.35 (s, 3H), 2.01 (t, *J* = 7.6 Hz, 2H), 1.87 – 1.77 (m, 2H).

^{13}C NMR (101 MHz, DMSO-*d*$_6$) δ 171.42, 167.26, 155.15, 144.91, 141.13, 140.98, 139.34, 137.41, 135.89, 129.89, 129.34, 128.87, 128.64, 125.72, 117.13, 48.52, 35.08, 32.61, 24.61, 20.74, 19.42, 11.86.

HRMS calculated for $C_{27}H_{30}N_5O_4$ $[M + H]^+$: 488.2292, found 488.2287.

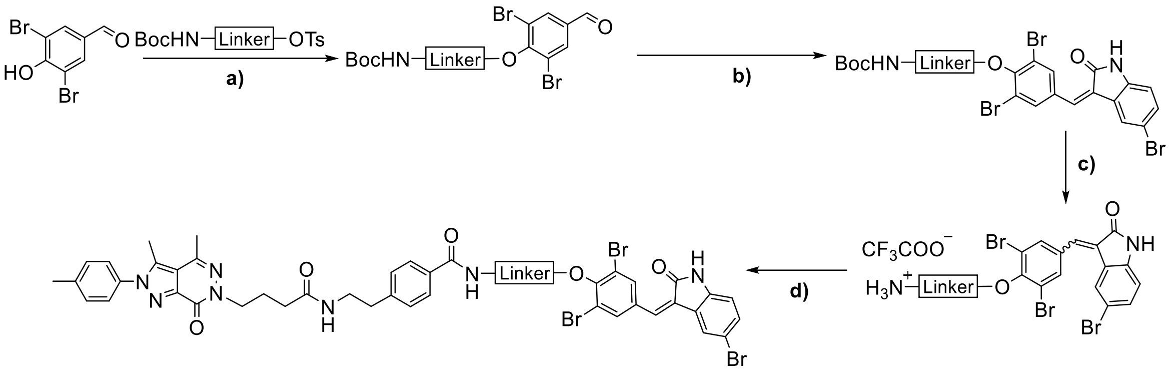

Supplementary Fig. 11 | Synthesis of compounds 5 and 6. **a)** K_2CO_3, DMF, 80 °C, 12 h; **b)** 5-bromooxindole, piperidine, EtOH, 90 °C, 6 h; **c)** TFA, DCM, rt, 1 h; **d) 56**, HATU, HOBt, DIPEA, DMF, rt, 4 h.

tert-butyl (2-(2-(2-(2-(2,6-dibromo-4-formylphenoxy)ethoxy)ethoxy)ethoxy)ethyl) carbamate (57)

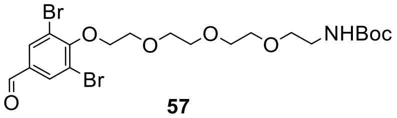

57

a): To a 25 mL flask, 3,5-dibromo-4-hydroxybenzaldehyde (0.28 g, 1 mmol, 1 eq.), *t*-Boc-*N*-amido-PEG4-Tos (0.5818 g, 1.3 mmol, 1.3 eq.), K_2CO_3 (0.4146 g, 3 mmol, 3 eq.) and anhydrous DMF (3 mL) were added. The resulting mixture was stirred at 80 °C for 12 h. After cooled to room temperature, the residue was dissolved in EtOAc, washed successively with water and brine. The organic layer was combined, dried over anhydrous Na_2SO_4, and concentrated under reduced pressure. The crude product was subjected to flash chromatography (pentane : EtOAc = 2 : 1). The final product **57** (0.3387 g, 70%) was obtained as a light yellow oil.

^{1}H NMR (400 MHz, $CDCl_3$) δ 9.85 (s, 1H), 8.02 (s, 2H), 4.32 – 4.25 (m, 2H), 3.98 – 3.87 (m, 2H), 3.80 – 3.74 (m, 2H), 3.70 – 3.60 (m, 6H), 3.53 (t, *J* = 5.1 Hz, 2H), 3.30 (t, *J* = 4.3 Hz, 2H), 1.43 (s, 9H).

^{13}C NMR (101 MHz, $CDCl_3$) δ 188.52, 158.44, 156.13, 134.27, 134.07, 119.46, 79.39, 73.12, 71.05, 70.82, 70.77, 70.44, 70.39, 70.25, 40.70, 28.57.

HRMS calculated for $C_{20}H_{30}Br_2NO_7$ $[M + H]^+$: 554.0384, found 554.0390.

tert-butyl (*Z*)-(2-(2-(2-(2-(2,6-dibromo-4-((5-bromo-2-oxoindolin-3-ylidene)methyl) phenoxy)ethoxy)ethoxy)ethoxy)ethyl)carbamate (58)

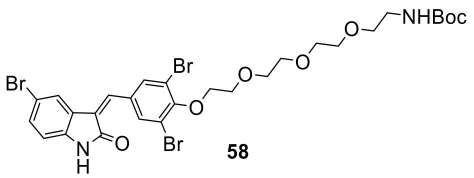

58

b): Piperidine (2 µL, 0.02 mmol, 0.1 eq.) were added into the solution of 5-bromooxindole (42.4 mg, 0.2 mmol, 1 eq.) and compound **57** (122.2 mg, 0.22 mmol, 1.1 eq.) in EtOH (3 mL) and stirred for 6 h at 90 ºC, then cooled to room temperature. After removing the solvent under reduced pressure, the product **58** (76.4 mg, 51%) could be obtained by flash chromatography (pentane : EtOAc = 2 : 3) as a red solid.

^{1}H NMR (400 MHz, DMSO-d_6) δ 10.82 (s, 1H), 8.76 (s, 2H), 7.94 – 7.78 (m, 2H), 7.38 (dd, J = 8.3, 2.0 Hz, 1H), 6.78 (d, J = 8.2 Hz, 1H), 6.70 (d, J = 6.2 Hz, 1H), 4.17 (t, J = 4.7 Hz, 2H), 3.81 (t, J = 4.7 Hz, 2H), 3.64 – 3.56 (m, 2H), 3.56 – 3.44 (m, 6H), 3.35 (t, J = 6.1 Hz, 2H), 3.03 (q, J = 6.0 Hz, 2H), 1.34 (s, 9H).

^{13}C NMR (126 MHz, DMSO-d_6) δ 166.68, 155.55, 154.18, 140.06, 136.20, 134.85, 132.64, 131.69, 126.91, 126.81, 122.86, 117.23, 113.21, 111.50, 77.55, 72.84, 69.93, 69.78, 69.50, 69.15, 28.22.

HRMS calculated for $C_{28}H_{34}Br_3N_2O_7$ [M + H]$^+$: 746.9911, found 746.9915.

(*Z*)-N-(2-(2-(2-(2-(2,6-dibromo-4-((5-bromo-2-oxoindolin-3-ylidene)methyl)phenoxy) ethoxy)ethoxy)ethoxy)ethyl)-4-(2-(4-(3,4-dimethyl-7-oxo-2-(p-tolyl)-2,7-dihydro-6H-pyrazolo[3,4-d] pyridazin-6-yl)butanamido)ethyl)benzamide (5)

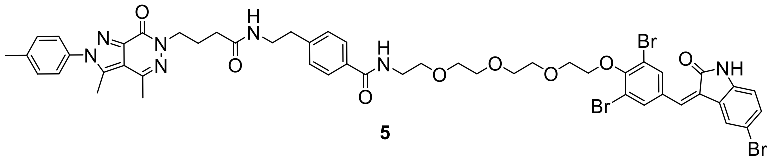

5

c-d): Compound **58** (45 mg, 0.06 mmol, 1.2 eq.) was added into a solution of TFA (1 mL) with DCM (1 mL). After stirring at room temperature for 1 h, the mixture was evaporated to remove the solvent, affording the amine intermediate **59** without further purification.

The mixture of compound **3** (24.4 mg, 0.05 mmol, 1 eq.), DIPEA (52.3 µL, 0.3 mmol, 6 eq.), HOBT (8.8 mg, 0.065 mmol, 1.3 eq.) and HATU (22.8 mg, 0.06 mmol, 1.2 eq.) in DMF (2 mL) was stirred for 10 min. After that, intermediate **59** was added into the resulting solution and stirred at room temperature for 4 h. The reaction mixture was diluted with

EtOAc, washed with water and brine, dried over Na_2SO_4, and concentrated in vacuo. Purification by preparative HPLC elution with 10-100% MeCN in H_2O (with 0.1% TFA) afforded the trifluoroacetic acid salt of (*Z*)-stereoisomer **5** (12.9 mg, 21%) as a yellow solid, which was then used for biological evaluation. Additionally, isomerization was detected when compound **5** was incubate in phosphate-buffered saline (PBS)/MeCN (v/v = 1/1) as a 100 µM solution at 37 ^{o}C, in which *Z/E* ratio decreased to 4.7 : 1 after 6 h and 1.2 : 1 after 18 h, respectively (Supplementary Fig. 10).

^{1}H NMR (400 MHz, DMSO-d_6) δ 10.81 (s, 1H), 8.74 (s, 2H), 8.38 (t, *J* = 5.6 Hz, 1H), 7.90 – 7.80 (m, 3H), 7.74 (d, *J* = 8.2 Hz, 2H), 7.47 – 7.38 (m, 4H), 7.36 (dd, *J* = 8.3, 2.0 Hz, 1H), 7.24 (d, *J* = 8.2 Hz, 2H), 6.77 (d, *J* = 8.3 Hz, 1H), 4.14 (t, *J* = 4.7 Hz, 2H), 3.99 (t, *J* = 7.0 Hz, 2H), 3.81 – 3.76 (m, 2H), 3.60 – 3.55 (m, 2H), 3.53 – 3.47 (m, 8H), 3.37 (q, *J* = 5.9 Hz, 2H), 3.25 (q, *J* = 6.9 Hz, 2H), 2.71 (t, *J* = 7.2 Hz, 2H), 2.55 (s, 3H), 2.48 (s, 3H), 2.40 (s, 3H), 2.10 – 2.03 (m, 2H), 1.88 (p, *J* = 7.6 Hz, 2H).

^{13}C NMR (176 MHz, DMSO-d_6) δ 171.40, 166.66, 166.09, 155.14, 154.15, 142.88, 141.12, 140.94, 140.04, 139.31, 137.35, 137.34, 136.19, 135.87, 134.82, 132.63, 132.27, 131.65, 129.87, 128.47, 127.20, 126.89, 126.80, 125.68, 122.83, 117.20, 117.13, 113.21, 111.48, 72.81, 69.92, 69.79, 69.78, 69.62, 69.48, 68.92, 48.55, 34.93, 32.62, 24.62, 20.73, 19.40, 11.85.

HRMS calculated for $C_{50}H_{53}Br_3N_7O_8$ $[M + H]^+$: 1116.1500, found 1116.1532.

HPLC method for stability test

Time (min)	%A	%B
0	90	10
1	90	10
2	40	60
7	20	80
8	5	95
10	5	95
12	90	10

A: ddH2O + 0.1% (v/v) formic acid; B: acetonitrile + 0.1% (v/v) formic acid; flow rate:0.25 mL/min.

Instruments: Velos Pro (Thermo Fisher Scientific, US), Ultimate 3000 HPLC (Thermo Fisher Scientific, US).

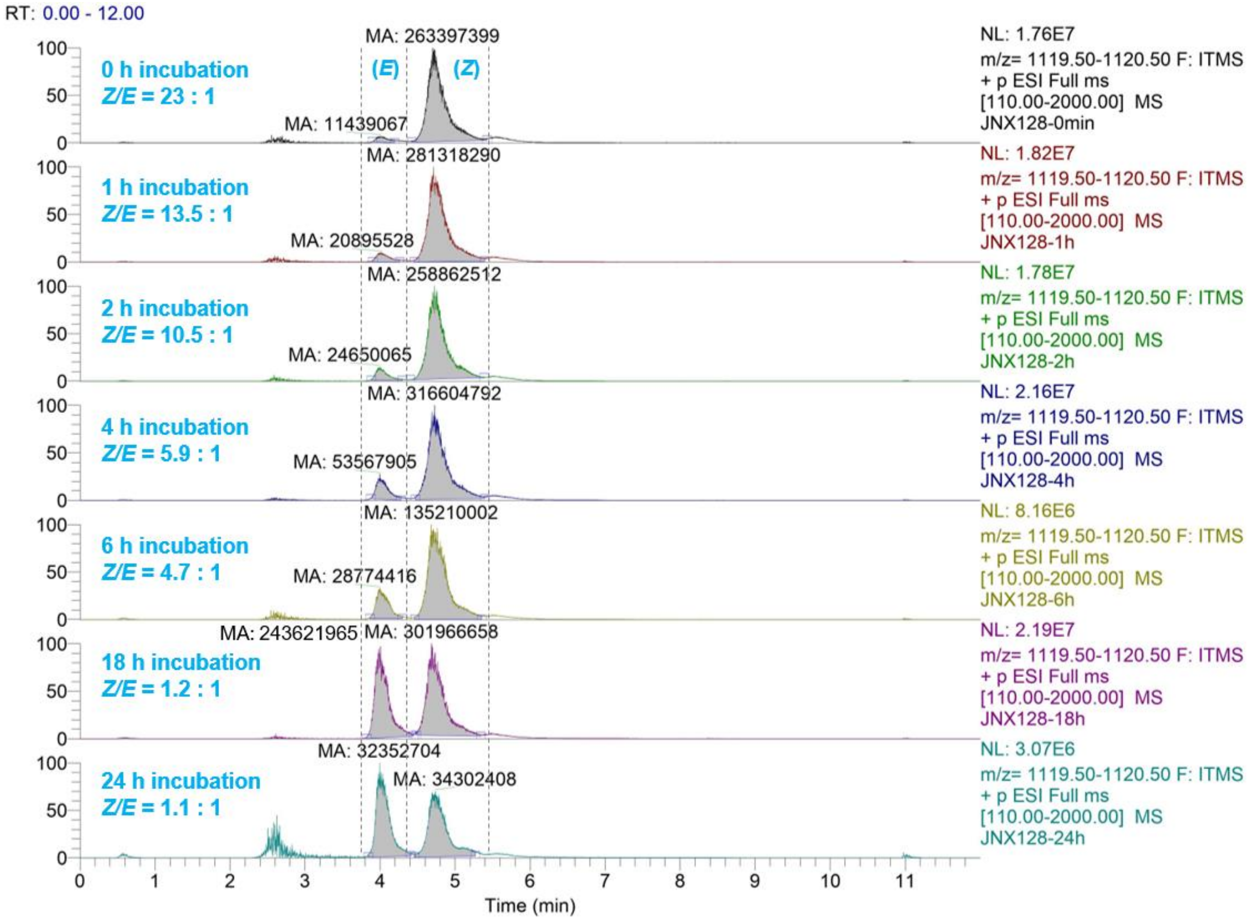

Supplementary Fig. 12 | Stability of the (*Z*)-configured compound 5. 100 µM compound **5** was incubate in PBS/MeCN (v/v = 1/1) for up to 24 h at 37 ºC. Then the solution was subjected to HPLC-ESI-MS analysis.

Following the same synthetic methods to get compound 6.

(*Z*)-N-(2-(2-(2-(2-(2,6-dibromo-4-((5-iodo-2-oxoindolin-3-ylidene)methyl)phenoxy) ethoxy)ethoxy)ethoxy)ethyl)-4-(2-(4-(3,4-dimethyl-7-oxo-2-(p-tolyl)-2,7-dihydro-6H-pyrazolo[3,4-d]pyridazin-6-yl)butanamido)ethyl)benzamide (6)

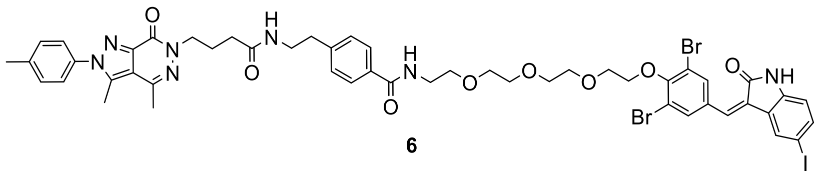

6

Compound **6** (7.6 mg, 13%) was obtained as a yellow solid.

^{1}H NMR (600 MHz, DMSO-d_6) δ 10.83 (s, 1H), 8.78 (s, 2H), 8.41 (t, *J* = 5.6 Hz, 1H), 8.04 (d, *J* = 1.7 Hz, 1H), 7.89 (t, *J* = 5.6 Hz, 1H), 7.85 (s, 1H), 7.76 (d, *J* = 8.3 Hz, 2H), 7.55 (dd, *J* = 8.1, 1.7 Hz, 1H), 7.51 – 7.46 (m, 2H), 7.43 (d, *J* = 8.3 Hz, 2H), 7.27 (d, *J* = 8.2 Hz, 2H), 6.69 (d, *J* = 8.1 Hz, 1H), 4.19 – 4.14 (m, 2H), 4.02 (t, *J* = 7.1 Hz, 2H), 3.82 – 3.80 (m, 2H), 3.60 (dd, *J* = 6.0, 3.7 Hz, 2H), 3.55 – 3.51 (m, 8H), 3.39 (q, *J* = 5.9 Hz, 2H), 3.27 (q, *J* = 6.9 Hz, 2H), 2.74 (t, *J* = 7.2 Hz, 2H), 2.59 (s, 3H), 2.43 (s, 3H), 2.09 (t, *J* = 7.6 Hz, 2H), 1.93 – 1.87 (m, 2H).

^{13}C NMR (151 MHz, DMSO-d_6) δ 171.38, 166.49, 166.08, 155.14, 154.11, 142.88, 141.12, 140.96, 140.48, 139.33, 137.46, 137.38, 136.18, 135.87, 134.59, 132.70, 132.26, 129.88, 128.48, 128.39, 127.20, 127.12, 126.72, 125.70, 117.20, 117.13, 111.95, 84.19, 72.81, 69.92, 69.78, 69.62, 69.48, 68.92, 48.54, 34.93, 32.61, 24.62, 20.74, 19.42, 11.86.

HRMS calculated for $C_{50}H_{53}Br_2IN_7O_8$ $[M + H]^+$: 1164.1362, found 1164.1383.

(*Z*)-4-(((4-(N-(4-chlorobenzyl)-N-cyclopentylsulfamoyl)-N-(piperidin-4-ylmethyl) phenyl)sulfonamido)methyl)-N-(2-(2-(2-(2-(2,6-dibromo-4-((5-bromo-2-oxoindolin-3-ylidene)methyl)phenoxy)ethoxy)ethoxy)ethoxy)ethyl)benzamide (7)

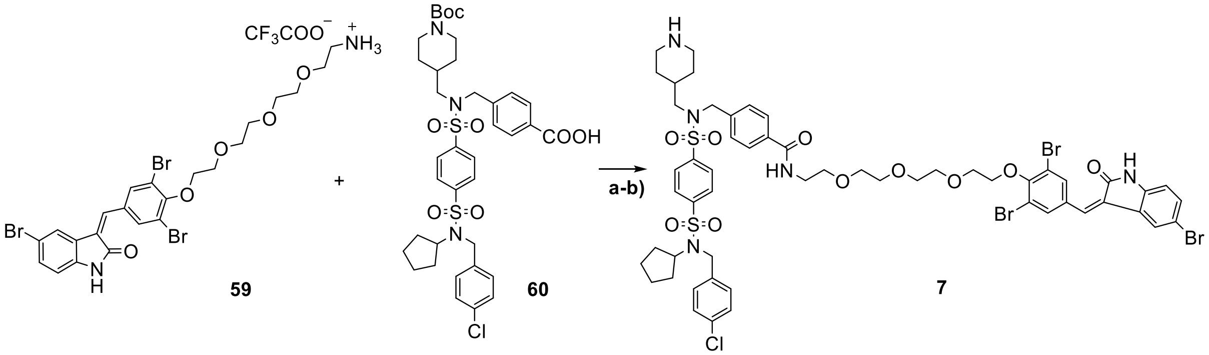

Supplementary Fig. 13 | Synthesis of compound 7. **a)** HATU, HOBt, DIPEA, DMF, rt, 4 h; **b)** TFA, DCM, rt, 1 h.

Compound **60** was synthesized according to the literature[2].

Compound **58** (45 mg, 0.06 mmol, 1.2 eq.) was added into a solution of TFA (1 mL) with DCM (1 mL). After stirring at room temperature for 1 h, the mixture was evaporated to remove the solvent, affording the amine intermediate **59** without further purification.

a): The mixture of compound **60** (38 mg, 0.05 mmol, 1 eq.), DIPEA (52.3 μL, 0.3 mmol, 6 eq.), HOBT (8.8 mg, 0.065 mmol, 1.3 eq.) and HATU (22.8 mg, 0.06 mmol, 1.2 eq.) in DMF (2 mL) was stirred for 10 min. After that, intermediate **59** was added into the resulting solution and stirred at room temperature for 4 h. The reaction mixture was diluted with EtOAc, washed with water and brine, dried over Na_2SO_4, and concentrated in vacuo. The product could be obtained by flash chromatography (DCM : MeOH = 20 : 1) as a yellow oil.

b): Afterwards, the product was dissolved into a solution of TFA (1 mL) with DCM (1 mL). After stirring at room temperature for 1 h, the mixture was evaporated to remove the solvent. Trifluoroacetic acid salt of **7** (12.6 mg, 18%) was obtained as a yellow solid by preparative HPLC with mobile phase of water and MeCN (0.1% TFA).

^{1}H NMR (700 MHz, DMSO-d_6) δ 10.79 (s, 1H), 8.71 (s, 2H), 8.50 (d, *J* = 11.2 Hz, 1H), 8.45 (t, *J* = 5.6 Hz, 1H), 8.18 (q, *J* = 11.0 Hz, 1H), 8.06 – 7.96 (m, 4H), 7.83 (d, *J* = 2.0 Hz, 1H), 7.80 (s, 1H), 7.74 (d, *J* = 8.2 Hz, 2H), 7.40 – 7.30 (m, 5H), 7.26 (d, *J* = 8.1 Hz, 2H), 6.74 (d, *J* = 8.2 Hz, 1H), 4.33 (d, *J* = 8.5 Hz, 4H), 4.24 – 4.18 (m, 1H), 4.12 – 4.08 (m, 2H), 3.78 – 3.72 (m, 2H), 3.55 – 3.52 (m, 2H), 3.50 – 3.42 (m, 8H), 3.34 (q, *J* = 5.9 Hz, 2H), 3.16 – 3.09 (m, 2H), 2.99 (d, *J* = 7.3 Hz, 2H), 2.58 (q, *J* = 12.0 Hz, 2H), 1.66 – 1.50 (m, 3H), 1.48 – 1.34 (m, 4H), 1.33 – 1.24 (m, 2H), 1.15 – 1.00 (m, 4H).

^{13}C NMR (176 MHz, DMSO-d_6) δ 166.69, 165.78, 158.42, 158.23, 158.05, 157.86, 154.15, 143.69, 142.53, 140.07, 139.45, 138.36, 136.20, 134.81, 133.72, 132.66, 131.70, 131.53, 128.67, 128.23, 128.17, 128.14, 128.04, 127.35, 126.94, 126.81, 122.85, 117.21, 113.23, 111.52, 72.82, 69.92, 69.78, 69.61, 69.49, 68.90, 59.14, 53.38, 51.73, 46.14, 42.64, 31.73, 28.61, 25.90, 22.89.

HRMS calculated for $C_{55}H_{62}Br_3ClN_5O_{10}S_2$ $[M + H]^+$: 1288.1171, found 1288.1211.

(*S*,*Z*)-2-(4-(4-chlorophenyl)-2,3,9-trimethyl-6H-thieno[3,2-f][1,2,4]triazolo[4,3-a][1,4] diazepin-6-yl)-N-(2-(2-(2-(2-(2,6-dibromo-4-((5-bromo-2-oxoindolin-3-ylidene) methyl)phenoxy)ethoxy)ethoxy)ethoxy)ethyl)acetamide (10)

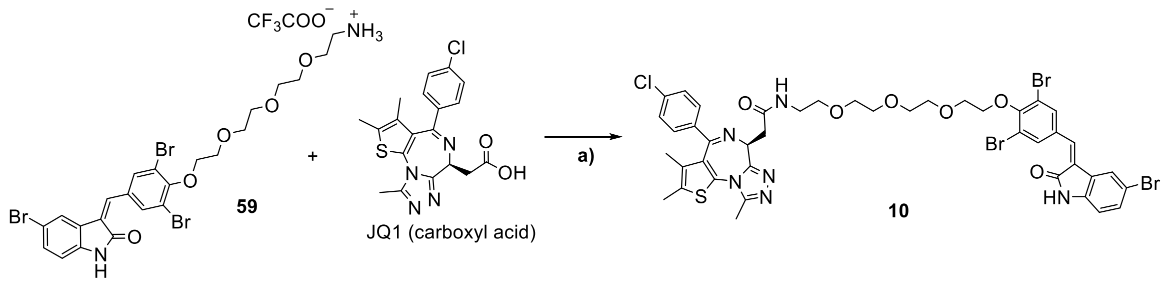

Supplementary Fig. 14 | Synthesis of compound 10. **a)** HATU, DIPEA, DMF, rt, 4 h.

Compound **58** (45 mg, 0.06 mmol, 1.2 eq.) was added into a solution of TFA (1 mL) with DCM (1 mL). After stirring at room temperature for 1 h, the mixture was evaporated to remove the solvent, affording the amine intermediate **59** without further purification.

The mixture of JQ1 carboxyl acid (20 mg, 0.05 mmol, 1 eq.), DIPEA (52.3 µL, 0.3 mmol, 6 eq.), and HATU (22.8 mg, 0.06 mmol, 1.2 eq.) in DMF (2 mL) was stirred for 10 min. After that, intermediate **59** was added into the resulting solution and stirred at room temperature for 4 h. The reaction mixture was diluted with EtOAc, washed with water and brine, dried over Na_2SO_4, and concentrated in vacuo. Trifluoroacetic acid salt of **10** (21.8 mg, 38%) was obtained as a yellow solid by preparative HPLC with mobile phase of water and MeCN (0.1% TFA).

^{1}H NMR (400 MHz, DMSO-*d*$_6$) δ 10.80 (s, 1H), 8.73 (s, 2H), 8.25 (t, *J* = 5.6 Hz, 1H), 7.86 (d, *J* = 2.0 Hz, 1H), 7.82 (s, 1H), 7.47 – 7.33 (m, 5H), 6.77 (d, *J* = 8.3 Hz, 1H), 4.51 (dd, *J* = 8.1, 6.0 Hz, 1H), 4.18 – 4.10 (m, 2H), 3.85 – 3.76 (m, 2H), 3.63 – 3.58 (m, 2H), 3.56 – 3.51 (m, 6H), 3.44 (t, *J* = 5.9 Hz, 2H), 3.33 – 3.15 (m, 4H), 2.58 (s, 3H), 2.37 (s, 3H), 1.58 (s, 3H).

^{13}C NMR (101 MHz, DMSO-*d*$_6$) δ 169.59, 166.67, 163.17, 155.03, 154.15, 149.96, 140.06, 136.59, 136.19, 135.33, 134.80, 132.63, 132.16, 131.66, 130.91, 130.19, 129.89, 129.63, 128.44, 126.92, 126.81, 122.84, 117.20, 113.22, 111.48, 72.83, 69.95, 69.82, 69.64, 69.50, 69.22, 53.74, 38.67, 37.40, 14.03, 12.66, 11.27.

HRMS calculated for $C_{42}H_{41}Br_3ClN_6O_6S$ $[M + H]^+$: 1029.0041, found 1029.0079.

Following the same synthetic methods to get other compounds 11 and 50.

(*S*,*Z*)-2-(4-(4-chlorophenyl)-2,3,9-trimethyl-6H-thieno[3,2-f][1,2,4]triazolo[4,3-a][1,4] diazepin-6-yl)-N-(2-(2-(2-(2-(2,6-dibromo-4-((5-iodo-2-oxoindolin-3-ylidene)methyl) phenoxy)ethoxy)ethoxy)ethoxy)ethyl)acetamide (11)

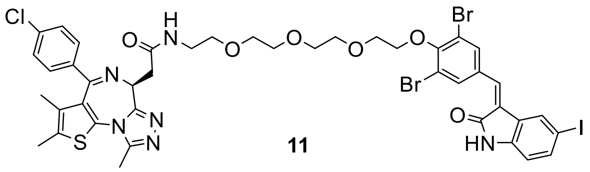

11

Compound **11** (8.4 mg, 14%) was obtained as a yellow solid.

^{1}H NMR (700 MHz, DMSO-*d*$_6$) δ 10.83 (s, 1H), 8.78 (s, 2H), 8.28 (t, *J* = 5.7 Hz, 1H), 8.05 (d, *J* = 1.7 Hz, 1H), 7.86 (s, 1H), 7.55 (dd, *J* = 8.1, 1.7 Hz, 1H), 7.51 – 7.46 (m, 2H), 7.42 (d, *J* = 8.2 Hz, 2H), 6.69 (d, *J* = 8.1 Hz, 1H), 4.52 (dd, *J* = 8.2, 5.9 Hz, 1H), 4.19 – 4.17 (m, 2H), 3.85 – 3.81 (m, 2H), 3.65 – 3.62 (m, 2H), 3.58 – 3.54 (m, 6H), 3.47 (t, *J* = 5.9 Hz, 2H), 3.33 – 3.19 (m, 4H), 2.60 (s, 3H), 2.40 (s, 3H), 1.61 (s, 3H).

^{13}C NMR (176 MHz, DMSO-*d*$_6$) δ 169.62, 166.50, 163.09, 158.56, 158.34, 158.13, 157.92, 155.06, 154.12, 149.89, 140.49, 137.47, 136.67, 136.18, 135.27, 134.60, 132.71, 132.23, 130.80, 130.18, 129.86, 129.64, 129.58, 128.46, 128.41, 127.13, 126.74, 117.22, 116.26, 114.60, 112.96, 111.97, 84.21, 72.84, 69.94, 69.82, 69.81, 69.64, 69.50, 69.22, 53.78, 38.65, 37.44, 14.06, 12.68, 11.30.

HRMS calculated for $C_{42}H_{41}Br_2ClIN_6O_6S$ [M + H]$^+$: 1076.9903, found 1076.9946.

(*S*,*Z*)-2-(4-(4-chlorophenyl)-2,3,9-trimethyl-6H-thieno[3,2-f][1,2,4]triazolo[4,3-a][1,4] diazepin-6-yl)-N-(2-(2-(2-(2,6-dibromo-4-((5-bromo-2-oxoindolin-3-ylidene)methyl) phenoxy)ethoxy)ethoxy)ethyl)acetamide (50)

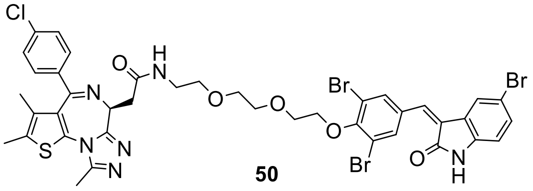

50

Compound **50** (13.8 mg, 25%) was obtained as a yellow solid.

^{1}H NMR (400 MHz, DMSO-*d*$_6$) δ 10.81 (s, 1H), 8.74 (s, 2H), 8.24 (t, *J* = 5.6 Hz, 1H), 7.87 (d, *J* = 2.0 Hz, 1H), 7.83 (s, 1H), 7.47 – 7.33 (m, 5H), 6.77 (d, *J* = 8.2 Hz, 1H), 4.54 – 4.46 (m, 1H), 4.21 – 4.11 (m, 2H), 3.86 – 3.79 (m, 2H), 3.66 – 3.60 (m, 2H), 3.60 – 3.53 (m, 2H), 3.46 (t, *J* = 5.8 Hz, 2H), 3.35 – 3.15 (m, 4H), 2.58 (s, 3H), 2.37 (s, 3H), 1.58 (s, 3H).

^{13}C NMR (101 MHz, DMSO-*d*$_6$) δ 169.58, 166.67, 163.15, 155.02, 154.15, 149.94, 140.06, 136.60, 136.18, 135.31, 134.81, 132.63, 132.16, 131.67, 130.88, 130.18, 129.88, 129.62, 128.44, 126.92, 126.81, 122.85, 117.21, 113.21, 111.47, 72.83, 69.89, 69.65, 69.51, 69.24, 53.74, 38.67, 37.41, 14.03, 12.66, 11.27.

HRMS calculated for $C_{40}H_{37}Br_3ClN_6O_5S$ [M + H]$^+$: 984.9779, found 984.9807.

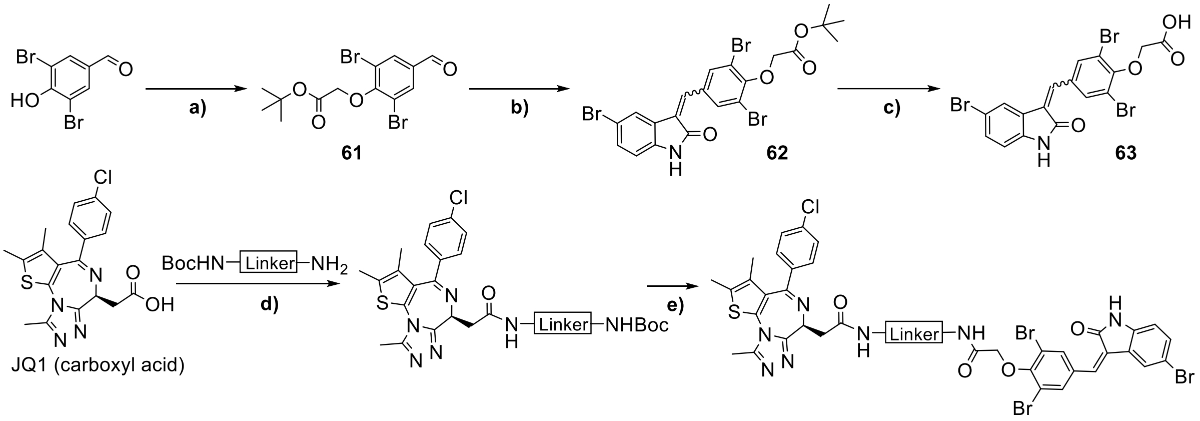

Supplementary Fig. 15 | Synthesis of compounds 9, 51-53. **a)** *tert*-butyl bromoacetate, K_2CO_3, DMF, 80 ^oC, 12 h; **b)** piperidine, EtOH, 90 ^oC, 6 h; **c)** TFA, DCM, rt, 2 h; **d)** HATU, DIPEA, DMF, rt, 4 h; **e)** (i) TFA, DCM, rt, 1 h; (ii) **63**, HATU, DIPEA, DMF, rt, 4 h.

tert-butyl 2-(2,6-dibromo-4-formylphenoxy)acetate (61)

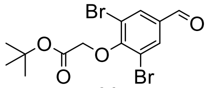

61

a): To a 25 mL flask, 3,5-dibromo-4-hydroxybenzaldehyde (0.56 g, 2 mmol, 1 eq.), *tert*-butyl bromoacetate (0.5852 g, 3 mmol, 1.5 eq.), K_2CO_3 (0.8292 g, 6 mmol, 3 eq.) and anhydrous DMF (5 mL) were added. The resulting mixture was stirred at 80 ^oC for 12 h. After cooled to room temperature, the residue was dissolved in EtOAc, washed successively with water and brine. The organic layer was combined, dried over anhydrous Na_2SO_4, and concentrated under reduced pressure. The crude product was subjected to flash chromatography (pentane : EtOAc = 6 : 1). The final product **61** (0.654 g, 83%) was obtained as a white solid.

^{1}H NMR (400 MHz, $CDCl_3$) δ 9.86 (s, 1H), 8.03 (s, 2H), 4.60 (s, 2H), 1.53 (s, 9H).

^{13}C NMR (126 MHz, $CDCl_3$) δ 188.45, 166.37, 157.39, 134.53, 134.06, 119.08, 82.85, 69.68, 28.21.

HRMS calculated for $C_{13}H_{15}Br_2O_4$ [M + H]$^+$: 392.9332, found 392.9337.

tert-butyl 2-(2,6-dibromo-4-((5-bromo-2-oxoindolin-3-ylidene)methyl)phenoxy) acetate (62)

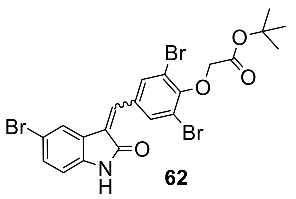
62

b): Piperidine (2 µL, 0.02 mmol, 0.1 eq.) were added into the solution of 5-bromooxindole (42.4 mg, 0.2 mmol, 1 eq.) and compound **61** (86.7 mg, 0.22 mmol, 1.1 eq.) in EtOH (3 mL) and stirred for 6 h at 90 ^oC, then cooled to room temperature. After removing the solvent under reduced pressure, the product **62** (89.4 mg, 76%) could be obtained by flash chromatography (pentane : EtOAc = 3 : 1) as a deep yellow solid.

^{1}H NMR (600 MHz, DMSO-d_6) δ 10.81 (s, 1H), 8.77 (s, 1H), 8.01 (s, 0.85H), 7.86 (d, J = 23.7 Hz, 1.13H), 7.55 (d, J = 34.3 Hz, 0.87H), 7.40 (d, 8.3 Hz, 1H), 6.81 (d, J = 7.7 Hz, 1H), 4.60 (s, 2H), 1.48 (s, 9H).

^{13}C NMR (151 MHz, DMSO-d_6) δ 167.73, 166.62, 166.20, 166.18, 152.93, 152.48, 142.40, 140.07, 136.17, 134.64, 133.82, 133.45, 133.31, 132.99, 132.88, 131.69, 128.17, 127.10, 126.75, 124.83, 122.85, 122.54, 117.53, 116.80, 113.21, 112.67, 112.19, 111.46, 81.74, 69.49, 69.45, 27.70.

HRMS calculated for $C_{21}H_{19}Br_3NO_4$ $[M + H]^+$: 585.8859, found 585.8858.

tert-butyl (*S*)-(1-(4-(4-chlorophenyl)-2,3,9-trimethyl-6H-thieno[3,2-f][1,2,4]triazolo [4,3-a][1,4]diazepin-6-yl)-2-oxo-6,9,12,15-tetraoxa-3-azaheptadecan-17-yl) carbamate (64)

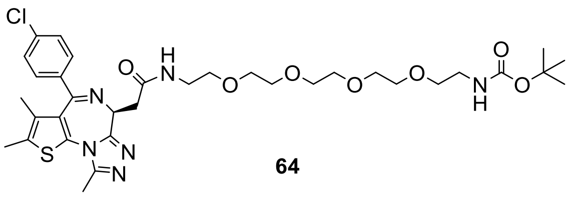
64

d): DIPEA (104.5 µL, 0.6 mmol, 3 eq.) and HATU (91.2 mg, 0.24 mmol, 1.2 eq.) were added sequentially into the solution of JQ1 carboxyl acid (80.2 mg, 0.2 mmol, 1 eq.) in DMF. After stirred for 10 min, the solution of *tert*-butyl (14-amino-3,6,9,12-tetraoxatetradecyl)carbamate (80.7 mg, 0.24 mmol, 1.2 eq. mmol) in DMF (2 mL) was added into the reaction mixture. After stirring at room temperature for 4 h, the reaction mixture was purified by flash column chromatography (DCM : MeOH = 15 : 1) to obtain the compound **64** (90.6 mg, 63%) as a slightly yellow solid.

^{1}H NMR (500 MHz, $CDCl_3$) δ 7.40 (d, *J* = 8.2 Hz, 2H), 7.31 (d, *J* = 8.4 Hz, 2H), 7.26 – 7.17 (m, 1H), 4.66 (t, *J* = 6.9 Hz, 1H), 3.69 – 3.58 (m, 14H), 3.54 – 3.48 (m, 5H), 3.40 (m, 1H), 3.29 (s, 2H), 2.66 (s, 3H), 2.39 (s, 3H), 1.66 (s, 3H), 1.41 (s, 9H).

^{13}C NMR (126 MHz, $CDCl_3$) δ 170.55, 163.90, 156.12, 155.66, 149.88, 136.82, 136.54, 132.08, 131.00, 130.97, 130.56, 129.95, 128.71, 79.03, 70.60, 70.56, 70.51, 70.34, 70.26, 70.18, 69.88, 54.30, 40.31, 39.44, 38.82, 28.46, 14.44, 13.13, 11.82.

HRMS calculated for $C_{34}H_{48}ClN_6O_7S$ $[M + H]^+$: 719.2988, found 719.3013.

(*S*,*Z*)-2-(4-(4-chlorophenyl)-2,3,9-trimethyl-6H-thieno[3,2-f][1,2,4]triazolo[4,3-a][1,4] diazepin-6-yl)-N-(1-(2,6-dibromo-4-((5-bromo-2-oxoindolin-3-ylidene)methyl) phenoxy)-2-oxo-6,9,12,15-tetraoxa-3-azaheptadecan-17-yl)acetamide (9)

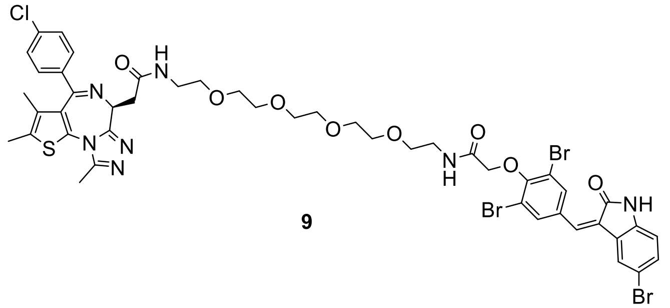

9

c, e): Compound **62** (58.8 mg, 0.1 mmol, 1 eq.) was dissolved in DCM (1 mL) and TFA (1 mL). After stirring at room temperature for 2 h, the mixture was evaporated to remove the solvent, affording the intermediate **63** without further purification.

Compound **64** (71.9 mg, 0.1 mmol, 1 eq.) was added into a solution of TFA (1 mL) with DCM (1 mL). After stirring at room temperature for 1 h, the mixture was evaporated to remove the solvent. DMF (3 mL), intermediate **63**, DIPEA (104.5 µL, 0.6 mmol, 6 eq.) and HATU (45.6 mg, 0.12 mmol, 1.2 eq.) were added sequentially to the residue. After stirring at room temperature for 4 h, the reaction mixture was diluted with EtOAc, washed with water and brine, dried over Na_2SO_4, and concentrated in vacuo. Trifluoroacetic acid salt of **9** (36.2 mg, 29%) was obtained as a yellow solid by preparative HPLC with mobile phase of water and MeCN (0.1% TFA).

^{1}H NMR (400 MHz, DMSO-*d*$_6$) δ 10.82 (s, 1H), 8.75 (s, 2H), 8.25 (t, *J* = 5.7 Hz, 1H), 8.04 (t, *J* = 5.7 Hz, 1H), 7.90 – 7.77 (m, 2H), 7.48 – 7.30 (m, 5H), 6.77 (d, *J* = 8.3 Hz, 1H), 4.55 – 4.47 (m, 1H), 4.44 (s, 2H), 3.58 – 3.39 (m, 16H), 3.37 – 3.14 (m, 6H), 2.57 (s, 3H), 2.37 (s, 3H), 1.58 (s, 3H).

^{13}C NMR (101 MHz, DMSO-*d*$_6$) δ 169.61, 166.64, 166.34, 163.13, 155.01, 152.81, 149.92, 140.11, 136.61, 136.15, 135.30, 134.57, 133.19, 132.17, 131.76, 130.86, 130.18, 129.87,

129.59, 128.44, 127.25, 126.73, 122.89, 116.97, 113.23, 111.51, 71.01, 69.82, 69.81, 69.79, 69.76, 69.61, 69.20, 68.71, 53.74, 38.65, 38.32, 37.40, 14.01, 12.65, 11.26.

HRMS calculated for $C_{46}H_{48}Br_3ClN_7O_8S$ [M + H]$^+$: 1130.0518, found 1130.0523.

Following the same synthetic methods to get other compounds 51-53.

(*S*,*Z*)-2-(4-(4-chlorophenyl)-2,3,9-trimethyl-6H-thieno[3,2-f][1,2,4]triazolo[4,3-a][1,4] diazepin-6-yl)-N-(1-(2,6-dibromo-4-((5-bromo-2-oxoindolin-3-ylidene)methyl) phenoxy)-2-oxo-6,9,12-trioxa-3-azatetradecan-14-yl)acetamide (51)

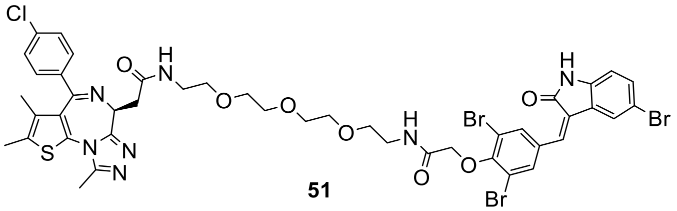

51

Compound **51** (38.4 mg, 32%) was obtained as a yellow solid.

^{1}H NMR (400 MHz, DMSO-d_6) δ 10.82 (s, 1H), 8.75 (s, 2H), 8.25 (t, J = 5.6 Hz, 1H), 8.05 (t, J = 5.5 Hz, 1H), 7.90 – 7.79 (m, 2H), 7.49 – 7.32 (m, 5H), 6.77 (d, J = 8.3 Hz, 1H), 4.53 – 4.40 (m, 3H), 3.55 – 3.41 (m, 12H), 3.38 – 3.15 (m, 6H), 2.56 (s, 3H), 2.37 (s, 3H), 1.57 (s, 3H).

^{13}C NMR (101 MHz, DMSO-d_6) δ 169.64, 166.63, 166.37, 166.33, 163.04, 155.02, 152.83, 149.81, 140.10, 136.67, 136.14, 135.23, 134.56, 133.17, 132.21, 131.76, 130.72, 130.13, 129.82, 129.53, 128.42, 127.22, 126.72, 122.86, 116.96, 113.21, 111.50, 71.02, 69.80, 69.78, 69.62, 69.60, 69.21, 68.72, 53.78, 38.64, 38.31, 37.46, 14.01, 12.64, 11.26.

HRMS calculated for $C_{44}H_{44}Br_3ClN_7O_4S$ [M + H]$^+$: 1086.0256, found 1086.0269.

(*S*,*Z*)-2-(4-(4-chlorophenyl)-2,3,9-trimethyl-6H-thieno[3,2-f][1,2,4]triazolo[4,3-a][1,4] diazepin-6-yl)-N-(1-(2,6-dibromo-4-((5-bromo-2-oxoindolin-3-ylidene)methyl) phenoxy)-2-oxo-6,9,12,15,18-pentaoxa-3-azaicosan-20-yl)acetamide (52)

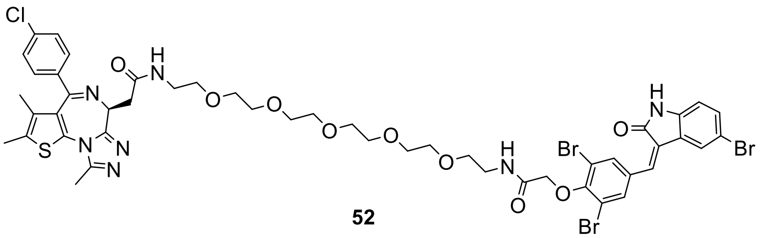

52

Compound **52** (16.8 mg, 13%) was obtained as a yellow solid.

^{1}H NMR (400 MHz, DMSO-*d*$_6$) δ 10.83 (s, 1H), 8.76 (s, 2H), 8.26 (t, *J* = 5.6 Hz, 1H), 8.05 (t, *J* = 5.7 Hz, 1H), 7.90 – 7.83 (m, 2H), 7.49 – 7.36 (m, 5H), 6.78 (d, *J* = 8.1 Hz, 1H), 4.56 – 4.49 (m, 1H), 4.46 (s, 2H), 3.55 – 3.42 (m, 20H), 3.37 – 3.18 (m, 6H), 2.59 (s, 3H), 2.39 (s, 3H), 1.59 (s, 3H).

^{13}C NMR (101 MHz, DMSO-*d*$_6$) δ 169.60, 166.64, 166.34, 163.15, 155.02, 152.82, 149.94, 140.12, 136.60, 136.16, 135.31, 134.57, 133.20, 132.16, 131.77, 130.89, 130.19, 129.88, 129.60, 128.44, 127.25, 126.74, 122.89, 116.97, 113.23, 111.51, 71.01, 69.81, 69.79, 69.78, 69.75, 69.61, 69.20, 68.71, 53.74, 40.43, 38.65, 38.32, 37.40, 14.01, 12.66, 11.26.

HRMS calculated for $C_{48}H_{52}Br_3ClN_7O_9S$ [M + H]$^+$: 1174.0780, found 1174.0788.

(*S*,*Z*)-2-(4-(4-chlorophenyl)-2,3,9-trimethyl-6H-thieno[3,2-f][1,2,4]triazolo[4,3-a][1,4] diazepin-6-yl)-N-(8-(2-(2,6-dibromo-4-((5-bromo-2-oxoindolin-3-ylidene)methyl) phenoxy)acetamido)octyl)acetamide (53)

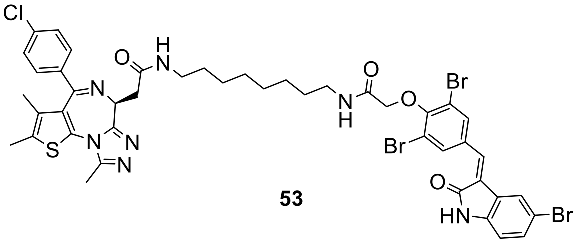

53

Compound **53** (27.7 mg, 24%) was obtained as a yellow solid.

^{1}H NMR (400 MHz, DMSO-*d*$_6$) δ 10.82 (s, 1H), 8.76 (s, 2H), 8.13 (t, *J* = 5.7 Hz, 1H), 8.08 (t, *J* = 5.9 Hz, 1H), 7.88 (d, *J* = 2.0 Hz, 1H), 7.85 (s, 1H), 7.47 – 7.36 (m, 5H), 6.78 (d, *J* = 8.3 Hz, 1H), 4.50 (dd, *J* = 8.1, 6.0 Hz, 1H), 4.41 (s, 2H), 3.27 – 3.03 (m, 6H), 2.57 (s, 3H), 2.38 (s, 3H), 1.59 (s, 3H), 1.49 – 1.38 (m, 4H), 1.31 – 1.22 (m, 8H).

^{13}C NMR (101 MHz, DMSO-*d*$_6$) δ 169.24, 166.64, 166.01, 163.06, 155.07, 152.83, 149.88, 140.12, 136.62, 136.17, 135.29, 134.60, 133.17, 132.18, 131.77, 130.83, 130.13, 129.84, 129.60, 128.42, 127.23, 126.74, 122.89, 117.00, 113.22, 111.52, 71.01, 53.86, 38.47, 38.34, 37.59, 29.24, 28.99, 28.78, 28.71, 26.37, 26.33, 14.02, 12.66, 11.26.

HRMS calculated for $C_{44}H_{44}Br_3ClN_7O_4S$ [M + H]$^+$: 1038.0409, found 1038.0414.

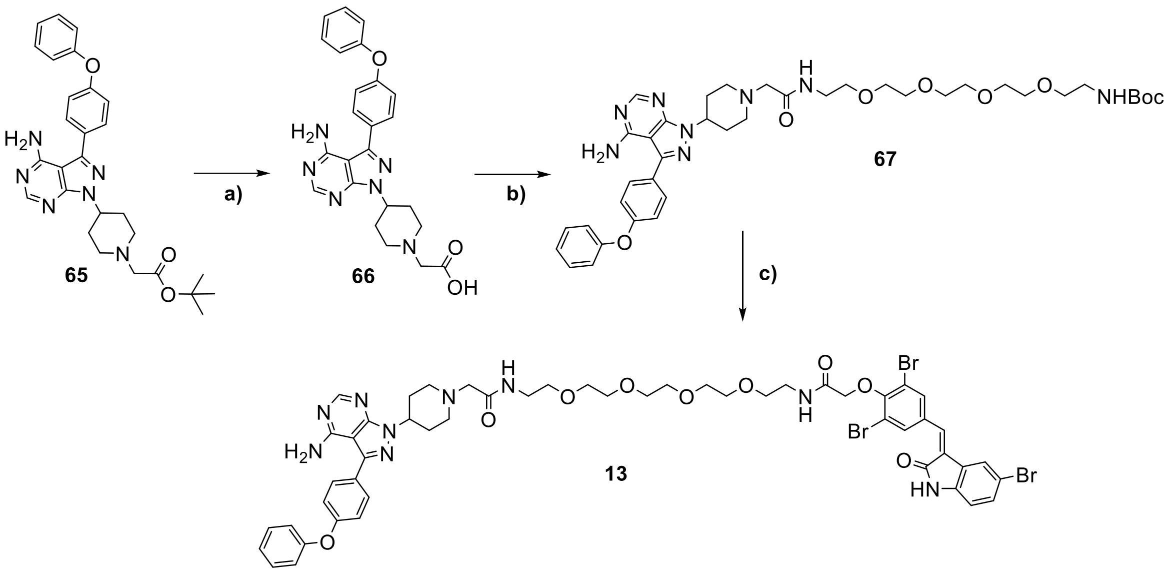

Supplementary Fig. 16 | Synthesis of compound 13. **a)** TFA, DCM, rt, 2 h; **b)** HATU, DIPEA, DMF, rt, 4 h; **c)** (i) TFA, DCM, rt, 1 h; (ii) **63**, HATU, DIPEA, DMF, rt, 4 h.

Compound **65** was synthesized according to the literature[6].

a): Compound **65** (100 mg, 0.2 mmol, 1 eq.) was dissolved in DCM (1 mL) and TFA (1 mL). After stirring at room temperature for 2 h, the mixture was evaporated to remove the solvent, affording the intermediate **66** without further purification.

b): DIPEA (209 µL, 1.2 mmol, 6 eq.) and HATU (91.2 mg, 0.24 mmol, 1.2 eq.) were added sequentially into the solution of compound **66** in DMF (3 mL). After stirred for 10 min, the solution of *tert*-butyl (14-amino-3,6,9,12-tetraoxatetradecyl)carbamate (80.7 mg, 0.24 mmol, 1.2 eq. mmol) in DMF (2 mL) was added into the reaction mixture. After stirring at room temperature for 4 h, the reaction mixture was purified by flash column chromatography (DCM : MeOH = 10 : 1) to obtain the compound **67** (44.2 mg, 29%) as a slightly yellow solid.

tert-butyl (1-(4-(4-amino-3-(4-phenoxyphenyl)-1H-pyrazolo[3,4-d]pyrimidin-1-yl)piperidin -1-yl)-2-oxo-6,9,12,15-tetraoxa-3-azaheptadecan-17-yl)carbamate (67)

^{1}H NMR (500 MHz, $CDCl_3$) δ 8.28 (s, 1H), 7.61 – 7.50 (m, 3H), 7.35 – 7.29 (m, 2H), 7.12 – 7.06 (m, 3H), 7.03 – 6.99 (m, 2H), 3.58 – 3.49 (m, 15H), 3.44 (td, J = 5.3, 3.0 Hz, 4H), 3.23 – 3.02 (m, 6H), 2.59 – 2.36 (m, 4H), 2.08 – 1.96 (m, 2H), 1.35 (s, 9H).

^{13}**C NMR** (126 MHz, $CDCl_3$) δ 158.59, 157.93, 156.43, 155.56, 153.99, 143.81, 130.08, 127.93, 124.16, 119.61, 119.20, 98.70, 79.30, 70.55, 70.51, 70.49, 70.32, 70.18, 70.07, 61.11, 53.23, 40.41, 38.96, 31.14, 28.52.

HRMS calculated for $C_{39}H_{55}N_8O_8$ $[M + H]^+$: 763.4137, found 763.4151.

c): Compound **62** (29.4 mg, 0.05 mmol, 1 eq.) was dissolved in DCM (1 mL) and TFA (1 mL). After stirring at room temperature for 2 h, the mixture was evaporated to remove the solvent, affording the intermediate **63** without further purification.

Compound **67** (38.1 mg, 0.05 mmol, 1 eq.) was added into a solution of TFA (1 mL) with DCM (1 mL). After stirring at room temperature for 1 h, the mixture was evaporated to remove the solvent. DMF (3 mL), intermediate **63**, DIPEA (52.3 µL, 0.3 mmol, 6 eq.) and HATU (22.8 mg, 0.06 mmol, 1.2 eq.) were added sequentially to the residue. After stirring at room temperature for 4 h, the reaction mixture was diluted with EtOAc, washed with water and brine, dried over Na_2SO_4, and concentrated in vacuo. Trifluoroacetic acid salt of **13** (12.3 mg, 19%) was obtained as a yellow solid by preparative HPLC with mobile phase of water and MeCN (0.1% TFA).

(*Z*)-2-(4-(4-amino-3-(4-phenoxyphenyl)-1H-pyrazolo[3,4-d]pyrimidin-1-yl)piperidin-1-yl)-N-(1-(2,6-dibromo-4-((5-bromo-2-oxoindolin-3-ylidene)methyl)phenoxy)-2-oxo-6,9,12,15-tetr aoxa-3-azaheptadecan-17-yl)acetamide (13)

^{1}**H NMR** (600 MHz, DMSO-d_6) δ 10.87 (s, 1H), 8.79 (s, 2H), 8.69 (t, J = 5.7 Hz, 1H), 8.36 (s, 1H), 8.11 (s, 1H), 7.91 (d, J = 2.0 Hz, 1H), 7.88 (s, 1H), 7.66 (d, J = 8.2 Hz, 2H), 7.47 – 7.43 (m, 2H), 7.41 (dd, J = 8.2, 2.0 Hz, 1H), 7.22 – 7.16 (m, 3H), 7.15 – 7.12 (m, 2H), 6.81 (d, J = 8.2 Hz, 1H), 5.10 – 5.00 (m, 1H), 4.47 (s, 2H), 3.97 (s, 2H), 3.64 (d, J = 11.8 Hz, 2H), 3.57 – 3.47 (m, 17H), 3.42 – 3.29 (m, 6H), 2.64 – 2.54 (m, 1H), 2.34 – 2.12 (m, 2H).

^{13}**C NMR** (151 MHz, DMSO-d_6) δ 166.67, 166.39, 164.03, 158.60, 158.37, 158.14, 157.92, 157.43, 156.72, 156.15, 153.25, 152.81, 144.19, 140.14, 136.18, 134.60, 133.24, 131.83, 130.17, 130.09, 127.31, 127.29, 126.74, 123.93, 122.92, 119.11, 118.95, 117.23, 117.01, 115.27, 113.32, 113.26, 111.57, 97.33, 71.01, 69.83, 69.80, 69.79, 69.76, 69.61, 68.78, 68.70, 56.60, 51.64, 50.76, 40.06, 38.85, 38.29, 28.06.

HRMS calculated for $C_{51}H_{55}Br_3N_9O_9$ $[M + H]^+$: 1174.1667, found 1174.1705.

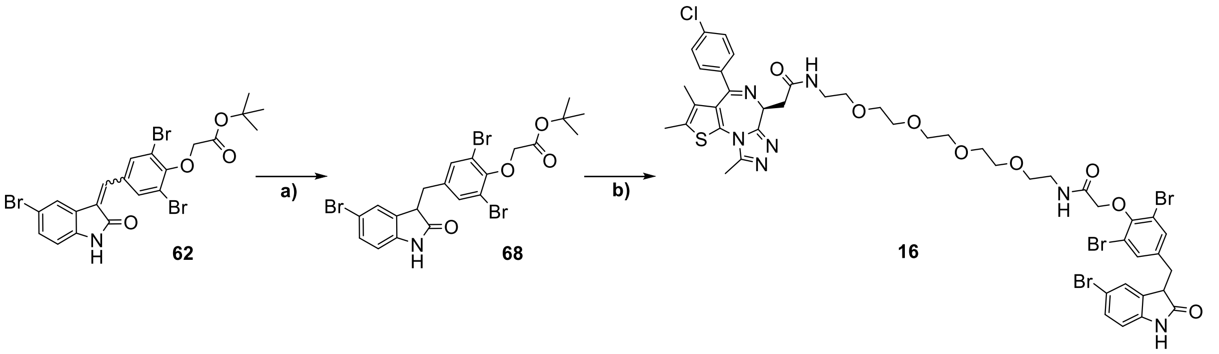

Supplementary Fig. 17 | Synthesis of compound 16. **a)** $NaBH_4$, DMF, EtOH, 0 °C, 2 min; **b)** (i) TFA, DCM, rt, 1 h; (ii) **64**, HATU, DIPEA, DMF, rt, 4 h.

a): In a 10 mL flask, compound **62** (117.6 mg, 0.2 mmol, 1 eq.) was dissolved in DMF/EtOH (v/v = 5/1, 4 mL) and the mixture was cooled down to 0 °C. $NaBH_4$ (45.6 mg, 1.2 mmol, 6 eq.) was added and stirred for 2 min. Afterwards, the reaction was diluted with EtOAc, washed with water and brine, dried over Na_2SO_4, and concentrated in vacuo. The reaction mixture was purified by flash column chromatography (pentane : EtOAc = 5 : 1) to give the compound **68** (73.2 mg, 62%) as a slightly yellow solid.

tert-butyl 2-(2,6-dibromo-4-((5-bromo-2-oxoindolin-3-yl)methyl)phenoxy)acetate (68)

^{1}H NMR (500 MHz, $CDCl_3$) δ 9.13 (s, 1H), 7.39 – 7.30 (m, 3H), 7.01 (s, 1H), 6.78 (d, *J* = 8.2 Hz, 1H), 4.48 (s, 2H), 3.70 (dd, *J* = 8.1, 5.1 Hz, 1H), 3.29 (dd, *J* = 14.0, 5.1 Hz, 1H), 2.95 (dd, *J* = 14.0, 8.0 Hz, 1H), 1.53 (s, 9H).

^{13}C NMR (126 MHz, $CDCl_3$) δ 178.55, 166.94, 151.39, 140.65, 136.47, 133.60, 131.38, 130.22, 127.78, 117.82, 114.99, 111.70, 82.49, 69.66, 47.15, 35.11, 28.22.

HRMS calculated for $C_{21}H_{21}Br_3NO_4$ $[M + H]^+$: 587.9015, found 587.9015.

b): Compound **68** (28 mg, 0.05 mmol, 1 eq.) was dissolved in DCM (1 mL) and TFA (1 mL). After stirring at room temperature for 2 h, the mixture was evaporated to remove the solvent, affording the hydrogenated **63** without further purification.

Compound **64** (35.9 mg, 0.05 mmol, 1 eq.) was added into a solution of TFA (1 mL) with DCM (1 mL). After stirring at room temperature for 1 h, the mixture was evaporated to remove the solvent. DMF (3 mL), hydrogenated **63**, DIPEA (52.3 µL, 0.3 mmol, 6 eq.) and HATU (22.8 mg, 0.06 mmol, 1.2 eq.) were added sequentially to the residue. After stirring at room temperature for 4 h, the reaction mixture was diluted with EtOAc, washed with water and brine, dried over Na_2SO_4, and concentrated in vacuo. Trifluoroacetic acid salt of **16** (27.8 mg, 49%) was obtained as a light yellow solid by preparative HPLC with mobile phase of water and MeCN (0.1% TFA).

2-((*S*)-4-(4-chlorophenyl)-2,3,9-trimethyl-6H-thieno[3,2-f][1,2,4]triazolo[4,3-a][1,4] diazepin-6-yl)-N-(1-(2,6-dibromo-4-((5-bromo-2-oxoindolin-3-yl)methyl)phenoxy)-2-oxo-6,9,12,15-tetraoxa-3-azaheptadecan-17-yl)acetamide (16)

^{1}H NMR (700 MHz, DMSO-d_6) δ 10.52 (s, 1H), 8.28 (t, *J* = 5.7 Hz, 1H), 8.01 (t, *J* = 5.8 Hz, 1H), 7.51 – 7.45 (m, 4H), 7.43 (d, *J* = 8.2 Hz, 2H), 7.32 (dd, *J* = 8.2, 2.0 Hz, 1H), 7.28 – 7.22 (m, 1H), 6.71 (d, *J* = 8.2 Hz, 1H), 4.55 – 4.50 (m, 1H), 4.32 (s, 2H), 3.89 (t, *J* = 6.4 Hz, 1H), 3.54 – 3.44 (m, 16H), 3.35 – 3.19 (m, 7H), 3.08 – 3.01 (m, 1H), 2.60 (s, 3H), 2.41 (s, 3H), 1.62 (s, 3H).

^{13}C NMR (101 MHz, DMSO-d_6) δ 177.38, 169.66, 166.44, 162.97, 155.09, 149.84, 149.76, 141.87, 137.96, 136.74, 135.20, 133.64, 132.25, 130.96, 130.66, 130.45, 130.12, 129.81, 129.54, 128.42, 127.32, 116.63, 112.85, 111.05, 70.83, 69.80, 69.75, 69.60, 69.57, 69.20, 68.67, 53.83, 46.09, 38.63, 38.23, 37.51, 33.22, 14.03, 12.65, 11.27.

HRMS calculated for $C_{46}H_{50}Br_3ClN_7O_8S$ [M + H]$^+$: 1132.0675, found 1132.0664.

2-((*S*)-4-(4-chlorophenyl)-2,3,9-trimethyl-6H-thieno[3,2-f][1,2,4]triazolo[4,3-a][1,4] diazepin-6-yl)-N-(2-(2-(2-(2-(2,6-dibromo-4-((5-bromo-2-oxoindolin-3-yl)methyl) phenoxy)ethoxy)ethoxy)ethoxy)ethyl)acetamide (17)

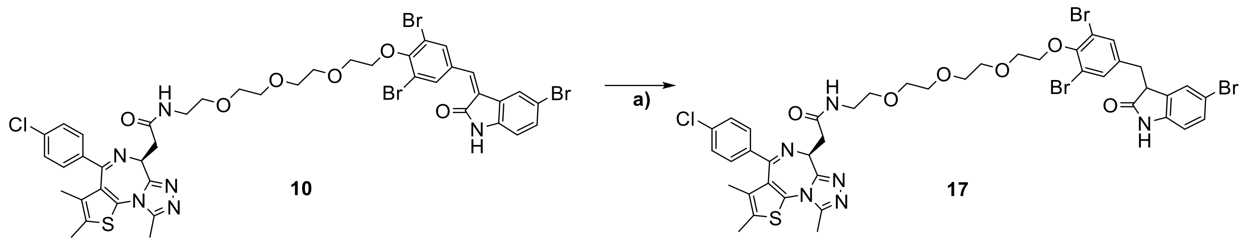

Supplementary Fig. 18 | Synthesis of compound 17. **a)** $NaBH_4$, DMF, EtOH, 0 ºC, 2 min.

In a 10 mL flask, compound **10** (51.6 mg, 0.05 mmol, 1 eq.) was dissolved in DMF/EtOH (v/v = 5/1, 2 mL) and the mixture was cooled down to 0 ºC. $NaBH_4$ (11.4 mg, 0.3 mmol, 6 eq.) was added and stirred for 2 min. Afterwards, the reaction was diluted with EtOAc, washed with water and brine, dried over Na_2SO_4, and concentrated in vacuo. Trifluoroacetic acid salt of **17** (26.9 mg, 52%) was obtained as a light yellow solid by preparative HPLC with mobile phase of water and MeCN (0.1% TFA).

^{1}H NMR (500 MHz, DMSO-d_6) δ 10.51 (s, 1H), 8.28 (t, *J* = 5.6 Hz, 1H), 7.48 (d, *J* = 8.1 Hz, 2H), 7.46 – 7.40 (m, 4H), 7.31 (dd, *J* = 8.2, 1.9 Hz, 1H), 7.26 (s, 1H), 6.70 (d, *J* = 8.2 Hz, 1H), 4.56 – 4.50 (m, 1H), 4.03 (t, *J* = 4.7 Hz, 2H), 3.88 (t, *J* = 6.3 Hz, 1H), 3.81 – 3.75

(m, 2H), 3.64 – 3.59 (m, 2H), 3.59 – 3.53 (m, 6H), 3.46 (t, *J* = 5.9 Hz, 2H), 3.34 – 3.17 (m, 5H), 3.03 (dd, *J* = 14.0, 6.5 Hz, 1H), 2.61 (s, 3H), 2.41 (s, 3H), 1.62 (s, 3H).

^{13}C NMR (126 MHz, DMSO-d_6) δ 177.45, 169.62, 163.13, 158.73, 158.43, 158.13, 157.84, 155.07, 151.05, 149.95, 141.88, 137.26, 136.65, 135.31, 133.60, 132.21, 131.04, 130.87, 130.48, 130.20, 129.89, 129.62, 128.47, 127.36, 118.85, 116.91, 116.54, 114.23, 112.88, 111.92, 111.06, 72.42, 69.91, 69.82, 69.65, 69.37, 69.23, 53.78, 46.15, 38.66, 37.43, 33.15, 14.07, 12.69, 11.30.

HRMS calculated for $C_{42}H_{43}Br_3ClN_6O_6S$ $[M + H]^+$: 1031.0198, found 1031.0233.

(*Z*)-N-(2-(2-(2-(2-(2,6-dibromo-4-((5-bromo-2-oxoindolin-3-ylidene)methyl)phenoxy) ethoxy)ethoxy)ethoxy)ethyl)-3-(5,5-difluoro-7,9-dimethyl-5H-dipyrrolo[1,2-c:2',1'-f] [1,3,2]diazaborinin-3-yl)propanamide (18)

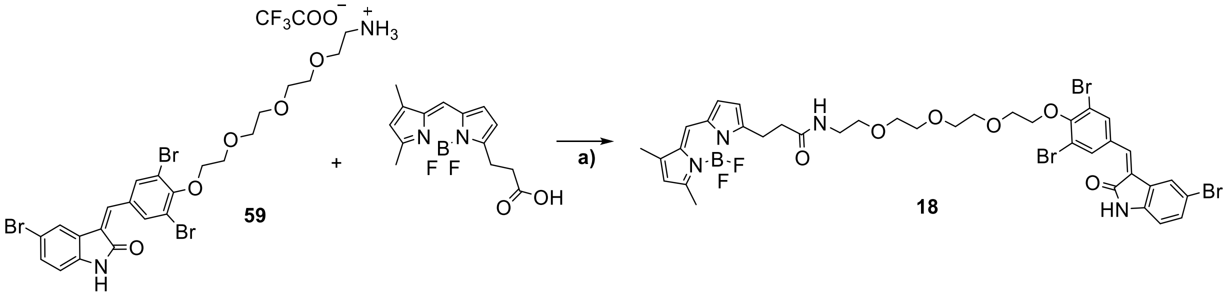

Supplementary Fig. 19 | Synthesis of compound 18. **a)** HATU, DIPEA, DMF, rt, 4 h.

Compound **58** (15.4 mg, 0.02 mmol, 1.2 eq.) was added into a solution of TFA (1 mL) with DCM (1 mL). After stirring at room temperature for 1 h, the mixture was evaporated to remove the solvent, affording the amine intermediate **59** without further purification.

The mixture of 3-Bodipy-propanoic acid (5 mg, 0.017 mmol, 1 eq.), DIPEA (17.8 µL, 0.1 mmol, 6 eq.), and HATU (7.6 mg, 0.02 mmol, 1.2 eq.) in DMF (2 mL) was stirred for 10 min. After that, intermediate **59** was added into the resulting solution and stirred at room temperature for 4 h. The reaction mixture was diluted with EtOAc, washed with water and brine, dried over Na_2SO_4, and concentrated in vacuo. Trifluoroacetic acid salt of **18** (21.8 mg, 38%) was obtained as a yellow solid by preparative HPLC with mobile phase of water and MeCN (0.1% TFA).

^{1}H NMR (700 MHz, DMSO-d_6) δ 10.85 (s, 1H), 8.78 (s, 2H), 7.99 (t, *J* = 5.7 Hz, 1H), 7.91 (d, *J* = 2.0 Hz, 1H), 7.87 (s, 1H), 7.66 (s, 1H), 7.40 (dd, *J* = 8.2, 2.0 Hz, 1H), 7.07 (d, *J* = 4.0 Hz, 1H), 6.81 (d, *J* = 8.2 Hz, 1H), 6.34 (d, *J* = 4.0 Hz, 1H), 6.28 (s, 1H), 4.19 – 4.16 (m, 2H), 3.85 – 3.80 (m, 2H), 3.62 (dd, *J* = 5.9, 3.8 Hz, 2H), 3.57 – 3.50 (m, 6H), 3.42 (t,

J = 5.8 Hz, 2H), 3.22 (q, *J* = 5.8 Hz, 2H), 3.07 (t, *J* = 7.7 Hz, 2H), 2.50 – 2.47 (m, 2H), 2.46 (s, 3H), 2.25 (s, 3H).

^{13}C NMR (176 MHz, DMSO-d_6) δ 170.90, 166.70, 159.07, 157.85, 154.17, 144.01, 140.06, 136.21, 134.87, 134.41, 132.96, 132.65, 131.70, 128.91, 126.90, 126.83, 125.29, 122.87, 120.23, 117.23, 116.59, 113.23, 111.51, 72.83, 69.94, 69.80, 69.60, 69.49, 69.12, 38.63, 33.64, 23.97, 14.50, 10.99.

HRMS calculated for $C_{37}H_{39}BBr_3F_2N_4O_6$ [M + H]$^+$: 921.0475, found 921.0496.

For compound 14 following the same synthetic methods according to Supplementary Fig. 11.

(Z)-N-(2-(2-(2-(2-(2,6-dibromo-4-((6-bromo-2-oxoindolin-3-ylidene)methyl)phenoxy) ethoxy)ethoxy)ethoxy)ethyl)-5-(2-oxohexahydro-1H-thieno[3,4-d]imidazol-4-yl)pentanamide (14)

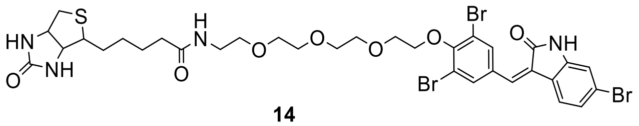

14

^{1}H NMR (600 MHz, DMSO-d_6) δ 10.86 (s, 1H), 8.78 (s, 2H), 7.91 (d, *J* = 2.0 Hz, 1H), 7.88 (s, 1H), 7.83 (t, *J* = 5.7 Hz, 1H), 7.41 (dd, *J* = 8.2, 2.0 Hz, 1H), 6.81 (d, *J* = 8.3 Hz, 1H), 6.41 (s, 1H), 6.35 (s, 1H), 4.30 (dd, *J* = 7.7, 5.0 Hz, 1H), 4.18 (dd, *J* = 5.7, 3.7 Hz, 2H), 4.16 – 4.08 (m, 1H), 3.83 – 3.78 (m, 2H), 3.62 (t, *J* = 3.3 Hz, 2H), 3.52 – 3.41 (m, 8H), 3.22 – 3.13 (m, 2H), 2.84 – 2.75 (m, 1H), 2.73 (t, *J* = 7.3 Hz, 2H), 2.06 (t, *J* = 7.5 Hz, 2H), 1.53 – 1.38 (m, 4H), 1.32 – 1.19 (m, 2H).

^{13}C NMR (151 MHz, DMSO-d_6) δ 172.66, 167.18, 163.19, 154.66, 140.51, 136.66, 135.33, 133.11, 132.20, 127.39, 127.27, 123.34, 117.71, 113.72, 112.01, 73.33, 70.40, 70.25, 70.05, 69.98, 69.62, 61.51, 59.66, 55.89, 38.93, 35.57, 28.67, 28.50, 25.73.

HRMS calculated for $C_{33}H_{40}Br_3N_4O_7S$ [M + H]$^+$: 873.0162, found 873.0166.

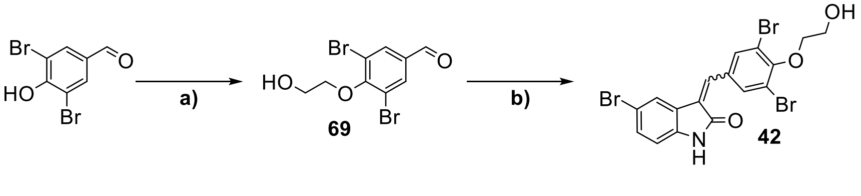

Supplementary Fig. 20 | Synthesis of compound 42. **a)** 2-bromoethanol, DMF, 80 ^{o}C, microwave, 3 h; **b)** 5-bromooxindole, piperidine, EtOH, 90 ^{o}C, 12 h.

a): To a 25 mL flask, 3,5-dibromo-4-hydroxybenzaldehyde (1.68 g, 6 mmol, 1 eq.), 2-bromoethanol (850 µL, 12 mmol, 2 eq.), K_2CO_3 (2.4876 g, 18 mmol, 3 eq.) and anhydrous DMF (8 mL) were added. The resulting mixture was stirred at 80 ^oC under microwave for 3 h. After cooled to room temperature, the residue was dissolved in EtOAc, washed successively with water and brine. The organic layer was combined, dried over anhydrous Na_2SO_4, and concentrated under reduced pressure. The crude product was subjected to flash chromatography (pentane : EtOAc = 5 : 1). The final product **69** (1.108 g, 57%) was obtained as a white solid.

3,5-dibromo-4-(2-hydroxyethoxy)benzaldehyde (69)

^{1}H NMR (500 MHz, $CDCl_3$) δ 9.88 (s, 1H), 8.06 (s, 2H), 4.33 – 4.27 (m, 2H), 4.07 – 4.01 (m, 2H).

^{13}C NMR (126 MHz, $CDCl_3$) δ 188.45, 157.83, 134.14, 131.09, 119.33, 75.44, 62.12.

HRMS calculated for $C_9H_9Br_2O_3$ $[M + H]^+$: 322.8913, found 322.8917.

b): Piperidine (2 µL, 0.02 mmol, 0.1 eq.) were added into the solution of 5-bromooxindole (42.4 mg, 0.2 mmol, 1 eq.) and compound **69** (71.3 mg, 0.22 mmol, 1.1 eq.) in EtOH (3 mL) and stirred for 12 h at 90 ^oC. The reaction solution was cooled to room temperature, filtered, and the filter cake was washed with cold MeOH (5 mL) for two times to provide **42** as a deep yellow solid (35.2 mg, 34%).

5-bromo-3-(3,5-dibromo-4-(2-hydroxyethoxy)benzylidene)indolin-2-one (42)

^{1}H NMR (700 MHz, DMSO-d_6) δ 10.85 (s, 1H), 8.78 (s, 2H), 7.91 (d, J = 2.0 Hz, 1H), 7.87 (s, 1H), 7.40 (dd, J = 8.2, 2.0 Hz, 1H), 6.80 (d, J = 8.2 Hz, 1H), 4.94 (t, J = 5.7 Hz, 1H), 4.07 (t, J = 5.4 Hz, 2H), 3.81 (q, J = 5.5 Hz, 2H).

^{13}C NMR (176 MHz, DMSO-d_6) δ 166.69, 154.25, 140.05, 136.22, 134.87, 132.61, 131.67, 126.88, 126.82, 122.86, 117.27, 113.22, 111.49, 75.11, 60.10.

HRMS calculated for $C_{17}H_{13}Br_3NO_3$ $[M + H]^+$: 515.8440, found 515.8436.

Following the same synthetic methods to get other compounds 33, 40-41, 43-47.

5,6-dichloro-3-(3,5-dibromo-4-(2-hydroxyethoxy)benzylidene)indolin-2-one (33)

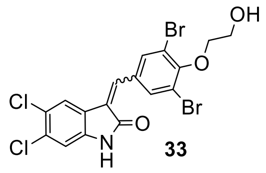

33

Compound **33** (29.5 mg, 29%) was obtained as a yellow solid.

^{1}H NMR (500 MHz, DMSO-d_6) δ 10.95 (s, 1H), 8.75 (s, 1.09H), 8.03 (s, 0.92H), 7.93 (d, J = 28.4 Hz, 1.14H), 7.63 (s, 0.46H), 7.54 (s, 0.52H), 7.07 (s, 0.47H), 7.01 (s, 0.56H), 4.94 (t, J = 5.6 Hz, 1H), 4.14 – 4.03 (m, 2H), 3.90 – 3.76 (m, 2H).

^{13}C NMR (126 MHz, DMSO-d_6) δ 167.86, 166.68, 154.41, 153.88, 143.07, 140.61, 136.26, 135.60, 134.68, 133.53, 132.70, 132.43, 132.24, 131.07, 127.24, 126.06, 125.34, 123.62, 123.47, 122.92, 121.81, 121.11, 118.04, 117.32, 111.78, 111.13, 75.13, 75.10, 60.10.

HRMS calculated for $C_{17}H_{12}Br_2Cl_2NO_3$ [M + H]$^+$: 505.8556, found 505.8561.

1-acetyl-5-bromo-3-(3,5-dibromo-4-(2-hydroxyethoxy)benzylidene)indolin-2-one (40)

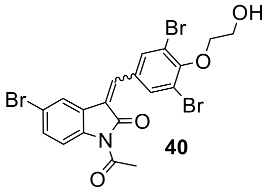

40

Compound **40** (19.0 mg, 17%) was obtained as a yellow solid.

^{1}H NMR (600 MHz, DMSO-d_6) δ 8.60 (s, 1.35H), 8.13 (d, J = 8.7 Hz, 0.32H), 8.06 (d, J = 2.1 Hz, 0.68H), 8.05 – 7.96 (m, 2H), 7.78 (s, 0.31H), 7.66 (d, J = 2.1 Hz, 0.31H), 7.59 (dd, J = 8.8, 2.1 Hz, 0.31H), 7.53 (dd, J = 8.7, 2.1 Hz, 0.68H), 5.00 – 4.89 (m, 1H), 4.16 – 4.03 (m, 2H), 3.88 – 3.76 (m, 2H), 2.62 (s, 3H).

^{13}C NMR (151 MHz, DMSO-d_6) δ 170.56, 170.32, 166.59, 165.16, 154.68, 154.17, 139.35, 137.45, 136.76, 136.35, 133.55, 132.90, 132.27, 132.04, 131.83, 126.84, 125.70, 124.34, 124.29, 123.27, 122.49, 118.08, 118.04, 117.61, 117.37, 117.08, 116.47, 75.17, 75.14, 60.11, 60.09, 26.64, 26.48.

HRMS calculated for $C_{19}H_{15}Br_3NO_4$ [M + H]$^+$: 557.8546, found 557.8540.

3-(3,5-dibromo-4-(2-hydroxyethoxy)benzylidene)-5-nitroindolin-2-one (41)

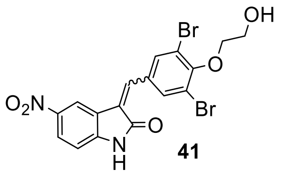

41

Compound **41** (28.1 mg, 29%) was obtained as a yellow solid.

^{1}H NMR (600 MHz, DMSO-*d*$_6$) δ 11.41 (s, 1H), 8.82 (s, 2H), 8.63 (d, *J* = 2.3 Hz, 1H), 8.17 (dd, *J* = 8.6, 2.3 Hz, 1H), 8.13 (s, 1H), 7.01 (d, *J* = 8.6 Hz, 1H), 4.94 (t, *J* = 5.7 Hz, 1H), 4.08 (t, *J* = 5.4 Hz, 2H), 3.82 (q, *J* = 5.4 Hz, 2H).

^{13}C NMR (151 MHz, DMSO-*d*$_6$) δ 167.30, 154.62, 146.35, 142.14, 136.69, 136.49, 132.37, 125.84, 125.73, 125.26, 117.35, 115.84, 109.63, 75.14, 60.11.

HRMS calculated for $C_{17}H_{13}Br_2N_2O_5$ [M + H]$^+$: 482.9186, found 482.9196.

4-bromo-3-(3,5-dibromo-4-(2-hydroxyethoxy)benzylidene)indolin-2-one (43)

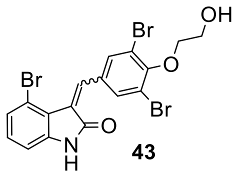

43

Compound **43** (35.2 mg, 34%) was obtained as a yellow solid.

^{1}H NMR (500 MHz, DMSO-*d*$_6$) δ 10.91 (s, 1H), 8.46 (s, 2H), 8.45 (s, 1H), 7.25 – 7.14 (m, 2H), 6.88 (d, *J* = 7.6 Hz, 1H), 4.94 (t, *J* = 5.7 Hz, 1H), 4.06 (t, *J* = 5.4 Hz, 2H), 3.82 (q, *J* = 5.5 Hz, 2H).

^{13}C NMR (126 MHz, DMSO-*d*$_6$) δ 166.11, 153.80, 143.58, 136.00, 135.82, 132.08, 130.74, 128.09, 126.43, 120.59, 116.89, 116.49, 109.09, 75.02, 60.08.

HRMS calculated for $C_{17}H_{13}Br_3NO_3$ [M + H]$^+$: 515.8440, found 515.8436.

7-bromo-3-(3,5-dibromo-4-(2-hydroxyethoxy)benzylidene)indolin-2-one (44)

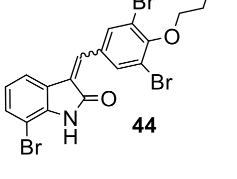

44

Compound **44** (59.0 mg, 57%) was obtained as a yellow solid.

^{1}H NMR (500 MHz, DMSO-d_6) δ 10.98 (s, 1H), 8.78 (s, 1.25H), 7.99 (s, 0.73H), 7.83 (s, 0.65H), 7.69 (d, J = 7.6 Hz, 0.66H), 7.60 (s, 0.42H), 7.50 – 7.37 (m, 1.41H), 6.99 (t, J = 7.8 Hz, 0.62H), 6.87 (t, J = 7.9 Hz, 0.38H), 4.99 – 4.90 (m, 1H), 4.08 (q, J = 5.4 Hz, 2H), 3.89 – 3.76 (m, 2H).

^{13}C NMR (126 MHz, DMSO-d_6) δ 168.03, 166.90, 154.23, 153.69, 140.06, 136.17, 134.86, 134.25, 133.43, 133.14, 132.97, 132.53, 131.94, 128.71, 127.63, 126.30, 122.85, 122.38, 121.21, 119.10, 117.97, 117.24, 102.93, 102.04, 75.11, 75.06, 60.10.

HRMS calculated for $C_{17}H_{13}Br_3NO_3$ [M + H]$^+$: 515.8440, found 515.8438.

5-bromo-3-(3,5-dibromo-4-(2-hydroxyethoxy)benzylidene)-1-methylindolin-2-one (45)

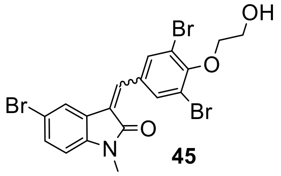

45

Compound **45** (20.2 mg, 19%) was obtained as a yellow solid.

^{1}H NMR (600 MHz, DMSO-d_6) δ 8.79 (s, 1.51H), 8.01 (s, 0.48H), 7.93 (d, J = 2.0 Hz, 0.75H), 7.89 (s, 0.75H), 7.67 (s, 0.23H), 7.56 – 7.52 (m, 0.48H), 7.48 (dd, J = 8.3, 1.9 Hz, 0.75H), 7.04 (d, J = 8.8 Hz, 0.22H), 6.98 (d, J = 8.3 Hz, 0.75H), 4.98 – 4.91 (m, 1H), 4.12 – 4.04 (m, 2H), 3.87 – 3.79 (m, 2H), 3.19 (s, 3H).

^{13}C NMR (151 MHz, DMSO-d_6) δ 166.38, 164.86, 154.35, 153.82, 143.47, 141.18, 136.28, 135.06, 134.59, 133.55, 132.77, 132.76, 132.46, 131.58, 127.03, 125.67, 124.55, 122.50, 121.86, 117.97, 117.28, 113.88, 113.33, 111.07, 110.51, 75.12, 75.10, 60.10, 60.09, 26.15, 26.04.

HRMS calculated for $C_{18}H_{15}Br_3NO_3$ [M + H]$^+$: 529.8597, found 529.8594.

6-bromo-3-(3,5-dibromo-4-(2-hydroxyethoxy)benzylidene)indolin-2-one (46)

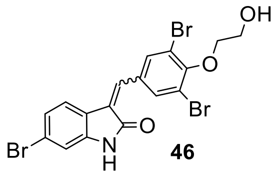

46

Compound **46** (58.0 mg, 56%) was obtained as a yellow solid.

^{1}H NMR (500 MHz, DMSO-d_6) δ 10.79 (s, 1H), 7.98 (s, 2H), 7.58 (s, 1H), 7.33 (d, J = 8.3 Hz, 1H), 7.13 – 7.08 (m, 1H), 7.04 (d, J = 1.8 Hz, 1H), 4.94 (t, J = 5.7 Hz, 1H), 4.08 (t, J = 5.4 Hz, 2H), 3.82 (q, J = 5.5 Hz, 2H).

^{13}C NMR (126 MHz, DMSO-d_6) δ 168.04, 153.62, 144.66, 133.35, 133.24, 133.17, 128.00, 123.96, 123.74, 123.18, 119.77, 118.01, 113.10, 75.04, 60.09.

HRMS calculated for $C_{17}H_{13}Br_3NO_3$ [M + H]$^+$: 515.8440, found 515.8437.

3-(3,5-dibromo-4-(2-hydroxyethoxy)benzylidene)-1-phenylindolin-2-one (47)

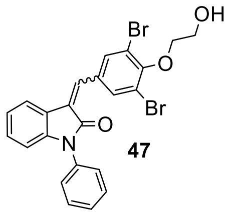

47

Compound **47** (23.7 mg, 23%) was obtained as a yellow solid.

^{1}H NMR (600 MHz, DMSO-d_6) δ 8.78 (s, 0.60H), 8.06 (s, 1.35H), 7.95 (s, 0.28H), 7.83 (dd, J = 7.7, 1.2 Hz, 0.29H), 7.72 (s, 0.66H), 7.63 – 7.58 (m, 2H), 7.56 (dd, J = 7.8, 1.1 Hz, 0.76H), 7.52 – 7.46 (m, 3H), 7.33 – 7.27 (m, 1H), 7.16 (t, J = 7.5 Hz, 0.31H), 7.03 (t, J = 7.3 Hz, 0.67H), 6.79 (d, J = 7.9 Hz, 0.70H), 6.75 (d, J = 7.8 Hz, 0.30H), 4.14 – 4.05 (m, 2H), 3.87 – 3.79 (m, 2H).

^{13}C NMR (151 MHz, DMSO-d_6) δ 166.32, 164.76, 154.12, 153.69, 144.04, 141.95, 136.12, 134.25, 134.16, 134.09, 133.81, 133.75, 133.44, 133.20, 132.64, 130.95, 130.72, 129.68, 129.57, 128.17, 128.10, 127.69, 126.95, 126.86, 126.60, 123.62, 122.51, 122.35, 120.23, 119.99, 118.00, 117.22, 109.62, 108.98, 75.11, 75.09, 60.12, 60.09.

HRMS calculated for $C_{23}H_{18}Br_2NO_3$ [M + H]$^+$: 513.9648, found 513.9654.

For compounds 48-49, 54-55 following the same synthetic methods according to Supplementary Fig. S11 and S13.

(*S*,*Z*)-2-(4-(4-chlorophenyl)-2,3,9-trimethyl-6H-thieno[3,2-f][1,2,4]triazolo[4,3-a][1,4] diazepin-6-yl)-N-(2-(2-(2-(2-(2,6-dibromo-4-((6-bromo-2-oxoindolin-3-ylidene) methyl)phenoxy)ethoxy)ethoxy)ethoxy)ethyl)acetamide (48)

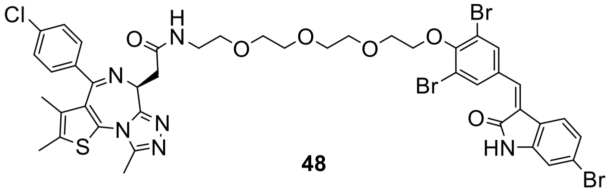

48

Compound **48** (17.5 mg, 34%) was obtained as a yellow solid.

^{1}H NMR (700 MHz, DMSO-d_6) δ 10.86 (s, 1H), 8.76 (s, 2H), 8.28 (t, *J* = 5.6 Hz, 1H), 7.82 (s, 1H), 7.61 (d, *J* = 8.1 Hz, 1H), 7.49 – 7.46 (m, 2H), 7.42 (d, *J* = 8.5 Hz, 2H), 7.22 (dd, *J* = 8.1, 1.8 Hz, 1H), 6.99 (d, *J* = 1.8 Hz, 1H), 4.51 (dd, *J* = 8.3, 5.9 Hz, 1H), 4.21 – 4.16 (m, 2H), 3.86 – 3.81 (m, 2H), 3.65 – 3.61 (m, 2H), 3.58 – 3.54 (m, 6H), 3.47 – 3.46 (m, 2H), 3.29 – 3.19 (m, 4H), 2.59 (s, 3H), 2.40 (s, 3H), 1.62 (s, 3H).

^{13}C NMR (176 MHz, DMSO-d_6) δ 169.66, 166.83, 163.01, 155.10, 154.03, 149.82, 142.30, 136.74, 136.04, 135.22, 134.06, 132.71, 132.27, 130.70, 130.15, 129.83, 129.56, 128.45, 127.05, 123.98, 123.83, 122.04, 121.80, 117.21, 112.34, 72.82, 69.94, 69.81, 69.64, 69.51, 69.22, 53.83, 38.64, 37.50, 14.07, 12.68, 11.30.

HRMS calculated for $C_{42}H_{41}Br_3ClN_6O_6S$ $[M + H]^+$: 1029.0041, found 1029.0071.

(*S*,*Z*)-2-(4-(4-chlorophenyl)-2,3,9-trimethyl-6H-thieno[3,2-f][1,2,4]triazolo[4,3-a][1,4] diazepin-6-yl)-N-(2-(2-(2-(2-(2,6-dibromo-4-((2-oxo-1-phenylindolin-3-ylidene) methyl)phenoxy)ethoxy)ethoxy)ethoxy)ethyl)acetamide (49)

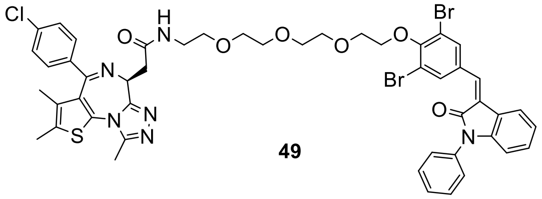

49

Compound **49** (10.9 mg, 19%) was obtained as a yellow solid.

^{1}H NMR (500 MHz, DMSO-d_6) δ 8.78 (s, 2H), 8.27 (t, *J* = 5.6 Hz, 1H), 7.95 (s, 1H), 7.83 (d, *J* = 7.5 Hz, 1H), 7.62 – 7.57 (m, 2H), 7.52 – 7.46 (m, 5H), 7.44 – 7.40 (m, 2H), 7.29 (td, *J* = 7.7, 1.2 Hz, 1H), 7.15 (td, *J* = 7.5, 1.0 Hz, 1H), 6.75 (d, *J* = 7.9 Hz, 1H), 4.53 – 4.49 (m, 1H), 4.19 – 4.17 (m, 2H), 3.86 – 3.81 (m, 2H), 3.65 – 3.61 (m, 2H), 3.58 – 3.53

(m, 6H), 3.46 (t, *J* = 5.9 Hz, 2H), 3.34 – 3.17 (m, 4H), 2.59 (s, 3H), 2.40 (s, 3H), 1.62 (s, 3H).

^{13}C NMR (126 MHz, DMSO-*d*$_6$) δ 169.60, 164.77, 163.06, 155.06, 154.04, 149.87, 141.96, 136.67, 136.11, 135.25, 134.23, 134.17, 132.67, 132.22, 130.78, 130.17, 129.85, 129.62, 129.57, 128.45, 128.10, 126.95, 126.63, 123.62, 122.53, 120.24, 117.18, 108.99, 72.84, 69.94, 69.81, 69.63, 69.50, 69.22, 53.78, 37.44, 14.05, 12.67, 11.29.

HRMS calculated for $C_{48}H_{46}Br_2ClN_6O_6S$ [M + Na]$^+$: 1050.9861, found 1050.9866.

(Z)-N-(2-(2-(2-(2-(2,6-dibromo-4-((6-bromo-2-oxoindolin-3-ylidene)methyl)phenoxy) ethoxy)ethoxy)ethoxy)ethyl)-4-(2-(4-(3,4-dimethyl-7-oxo-2-(p-tolyl)-2,7-dihydro-6H-pyrazolo[3,4-d]pyridazin-6-yl)butanamido)ethyl)benzamide (54)

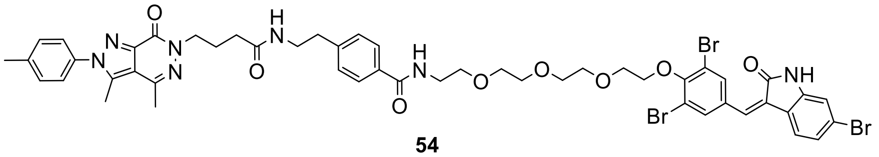

54

Compound **54** (6.7 mg, 11%) was obtained as a yellow solid.

^{1}H NMR (600 MHz, DMSO-*d*$_6$) δ 10.84 (s, 1H), 8.73 (s, 2H), 8.40 (t, *J* = 5.7 Hz, 1H), 7.89 (t, *J* = 5.7 Hz, 1H), 7.79 (s, 1H), 7.77 – 7.71 (m, 2H), 7.59 (d, *J* = 8.1 Hz, 1H), 7.48 – 7.39 (m, 4H), 7.29 – 7.23 (m, 2H), 7.19 (dd, *J* = 8.1, 1.8 Hz, 1H), 6.99 (d, *J* = 1.8 Hz, 1H), 4.15 (q, *J* = 4.5 Hz, 2H), 4.00 (t, *J* = 7.1 Hz, 2H), 3.84 – 3.77 (m, 2H), 3.59 – 3.54 (m, 2H), 3.54 – 3.50 (m, 8H), 3.26 (q, *J* = 7.0 Hz, 2H), 2.72 (t, *J* = 7.3 Hz, 2H), 2.57 (s, 3H), 2.42 (s, 3H), 2.11 – 2.05 (m, 2H), 1.89 (p, *J* = 7.4 Hz, 2H).

^{13}C NMR (151 MHz, DMSO-*d*$_6$) δ171.59, 166.92, 166.26, 155.27, 154.11, 142.99, 142.34, 141.19, 141.17, 139.46, 137.53, 136.13, 136.07, 135.94, 132.76, 132.32, 129.99, 128.60, 127.10, 125.79, 123.87, 122.13, 118.02, 117.27, 117.22, 112.41, 112.45, 72.89, 70.00, 69.86, 69.85 69.70, 69.58, 68.98, 48.64, 34.98, 32.70, 24.69, 20.82, 19.49, 11.93.

HRMS calculated for $C_{50}H_{53}Br_3N_7O_8$ [M + H]$^+$: 1116.1500, found 1116.1530.

(Z)-N-(2-(2-(2-(2-(2,6-dibromo-4-((2-oxo-1-phenylindolin-3-ylidene)methyl)phenoxy) ethoxy)ethoxy)ethoxy)ethyl)-4-(2-(4-(3,4-dimethyl-7-oxo-2-(p-tolyl)-2,7-dihydro-6H-pyrazolo[3,4-d]pyridazin-6-yl)butanamido)ethyl)benzamide (55)

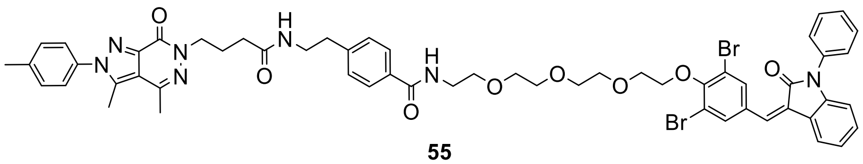

55

Compound **55** (4.9 mg, 8%) was obtained as a yellow solid.

^{1}H NMR (600 MHz, DMSO-d_6) δ 8.76 (s, 2H), 8.41 (t, *J* = 5.7 Hz, 1H), 7.93 (s, 1H), 7.89 (t, *J* = 5.7 Hz, 1H), 7.82 (dd, *J* = 7.5, 1.2 Hz, 1H), 7.79 – 7.73 (m, 2H), 7.63 – 7.57 (m, 2H), 7.51 – 7.41 (m, 7H), 7.31 – 7.24 (m, 3H), 7.14 (td, *J* = 7.5, 1.0 Hz, 1H), 6.75 – 6.72 (m, 1H), 4.16 (dd, *J* = 5.7, 3.7 Hz, 2H), 4.01 (t, *J* = 7.1 Hz, 2H), 3.83 – 3.78 (m, 2H), 3.59 (t, *J* = 3.2 Hz, 2H), 3.50 – 3.41 (m, 10H), 3.29 – 3.24 (m, 2H), 2.73 (t, *J* = 7.3 Hz, 2H), 2.58 (s, 3H), 2.42 (s, 3H), 2.12 – 2.06 (m, 2H), 1.94 – 1.85 (m, 2H).

^{13}C NMR (151 MHz, DMSO-d_6) δ 171.96, 166.63, 165.25, 155.65, 154.51, 143.36, 142.41, 141.58, 141.54, 139.84, 137.91, 136.55, 136.32, 134.69, 134.61, 133.12, 132.70, 130.37, 130.11, 130.06, 128.97, 128.60, 127.66, 127.41, 127.09, 126.17, 124.06, 123.02, 120.72, 117.63, 117.61, 109.45, 73.30, 70.38, 70.24, 70.08, 69.96, 69.36, 49.02, 35.36, 33.08, 25.07, 21.20, 19.87, 12.31.

HRMS calculated for $C_{56}H_{58}Br_2N_7O_8$ $[M + H]^+$: 1114.2708, found 1114.2721.

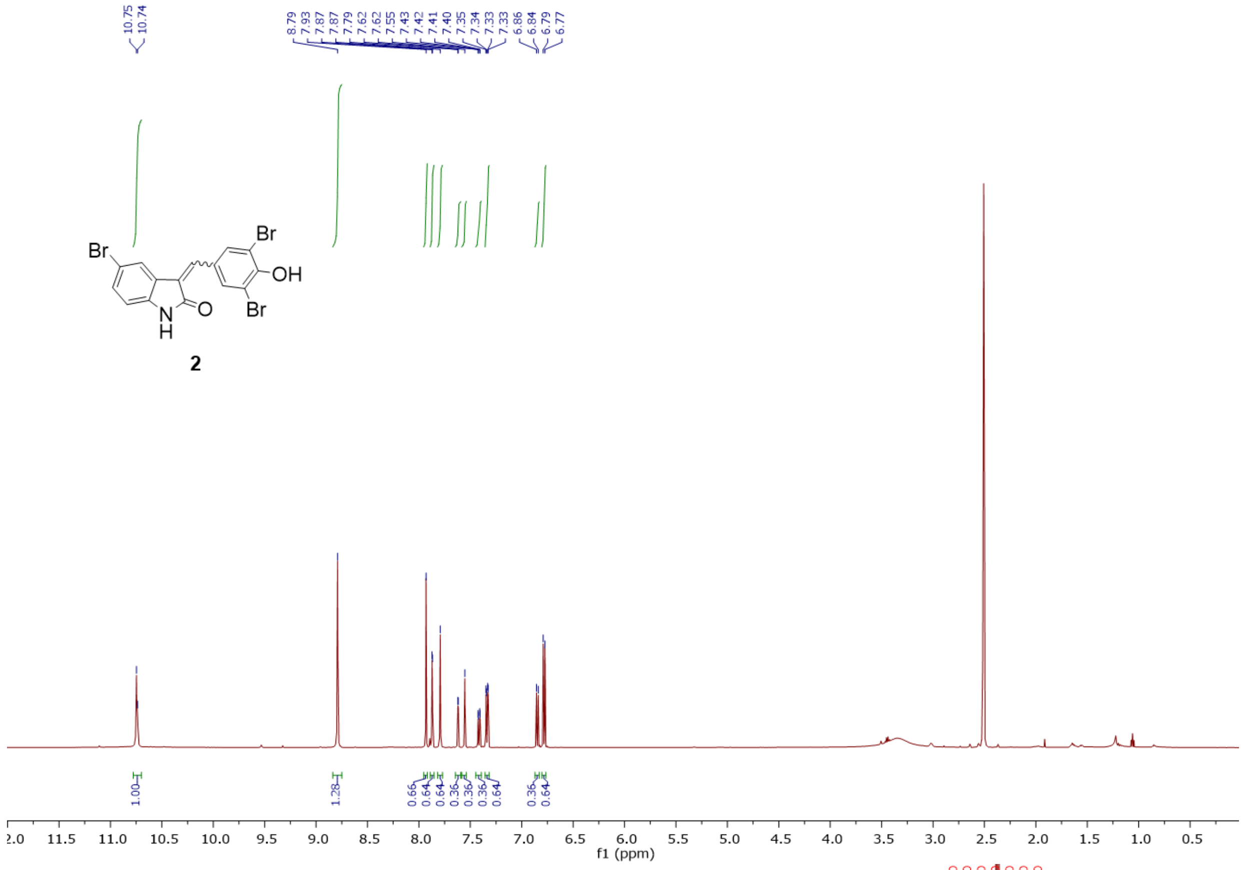
10.75
10.74
8.79
7.93
7.87
7.87
7.79
7.62
7.62
7.55
7.43
7.42
7.41
7.40
7.35
7.34
7.33
7.33
6.86
6.84
6.79
6.77
Br
Br
OH
Br
N
H
O
2
1.00
1.28
0.66
0.64
0.64
0.36
0.36
0.36
0.64
0.36
0.64
f1 (ppm)

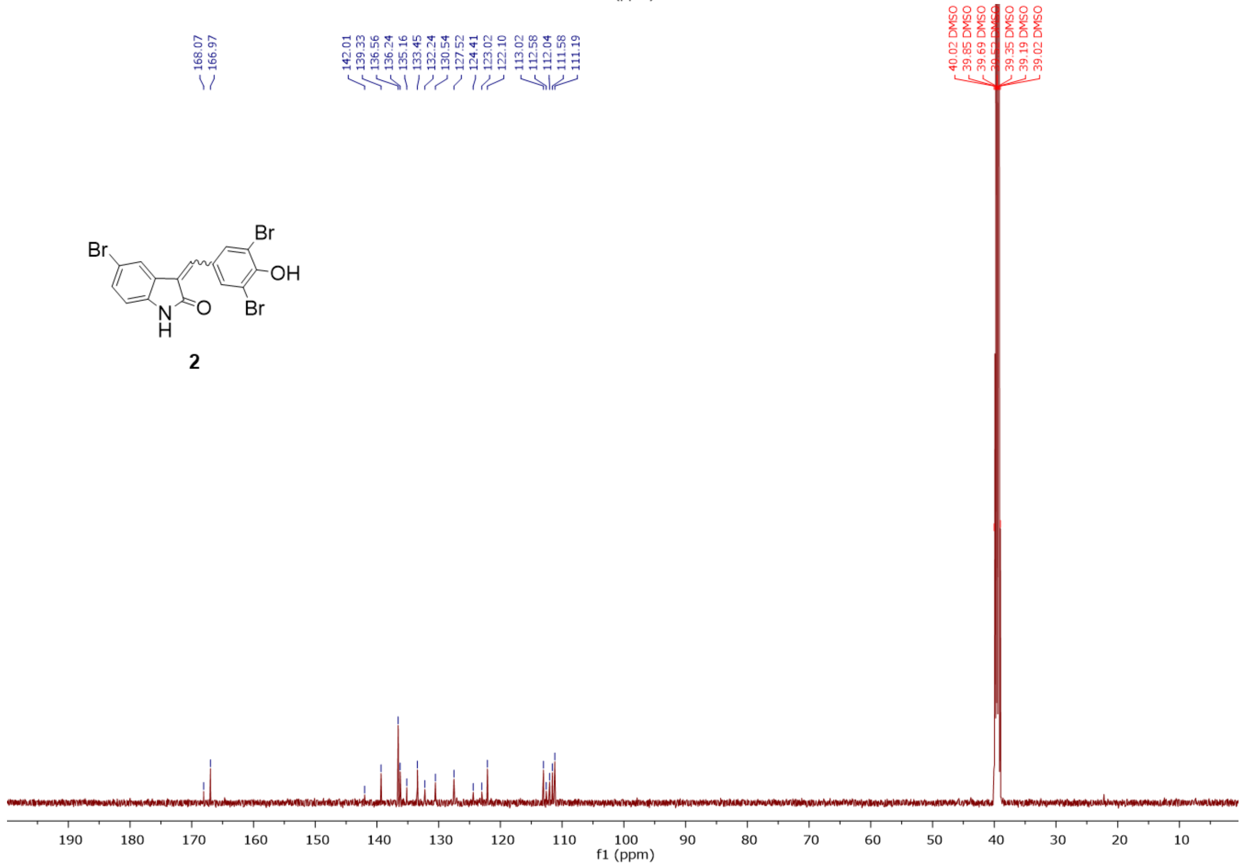
168.07
166.97
142.01
139.33
136.56
136.24
135.16
133.45
132.24
130.54
127.52
124.41
123.02
122.10
113.02
112.58
112.04
111.58
111.19
40.02 DMSO
39.85 DMSO
39.69 DMSO
39.35 DMSO
39.19 DMSO
39.02 DMSO
Br
Br
OH
Br
N
H
O
2
f1 (ppm)

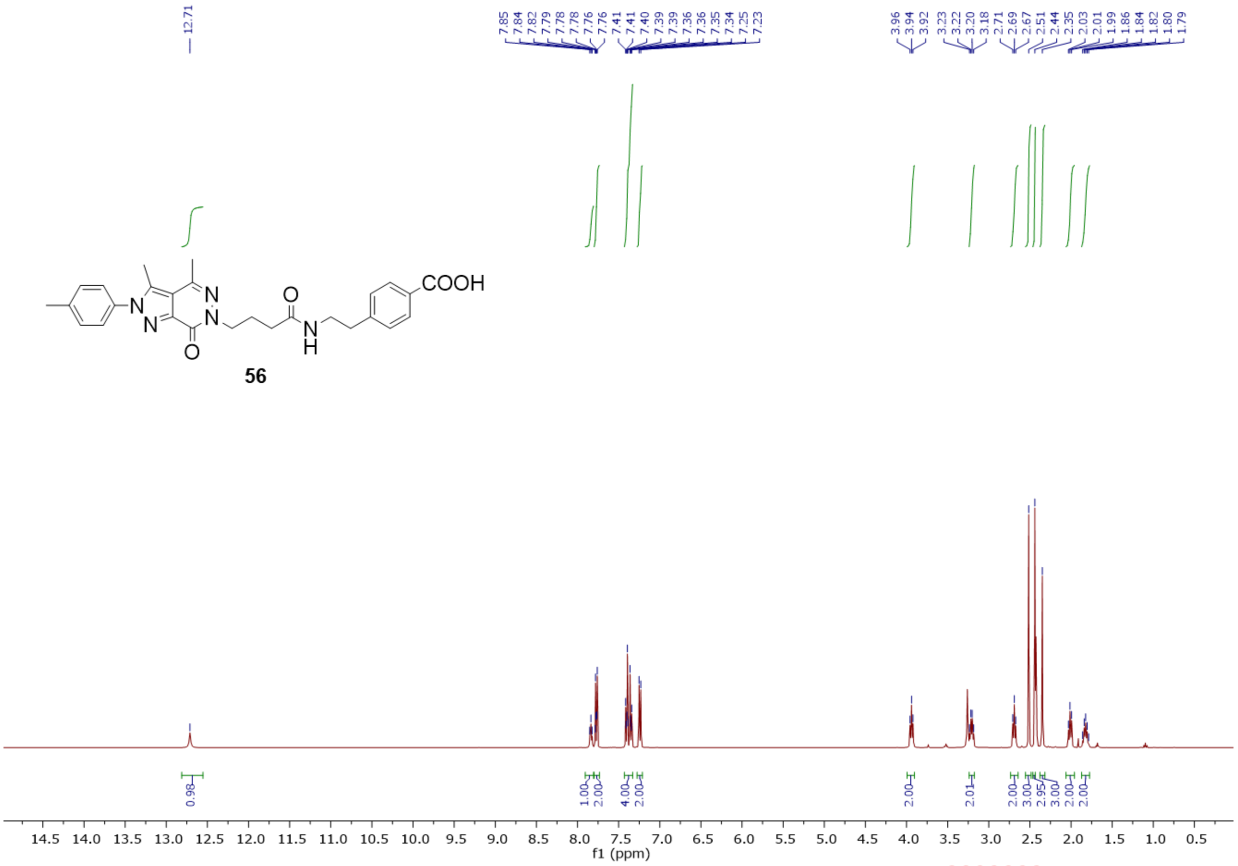
12.71
7.85 7.84 7.82 7.79 7.78 7.78 7.76 7.76 7.41 7.41 7.40 7.39 7.39 7.36 7.36 7.35 7.34 7.25 7.23
3.96 3.94 3.92 3.23 3.22 3.20 3.18 2.71 2.69 2.67 2.51 2.44 2.35 2.03 2.01 1.99 1.86 1.84 1.82 1.80 1.79
COOH
56
0.98
1.00 2.00 4.00 2.00
2.00 2.01 2.00 3.00 2.95 3.00 2.00 2.00
f1 (ppm)

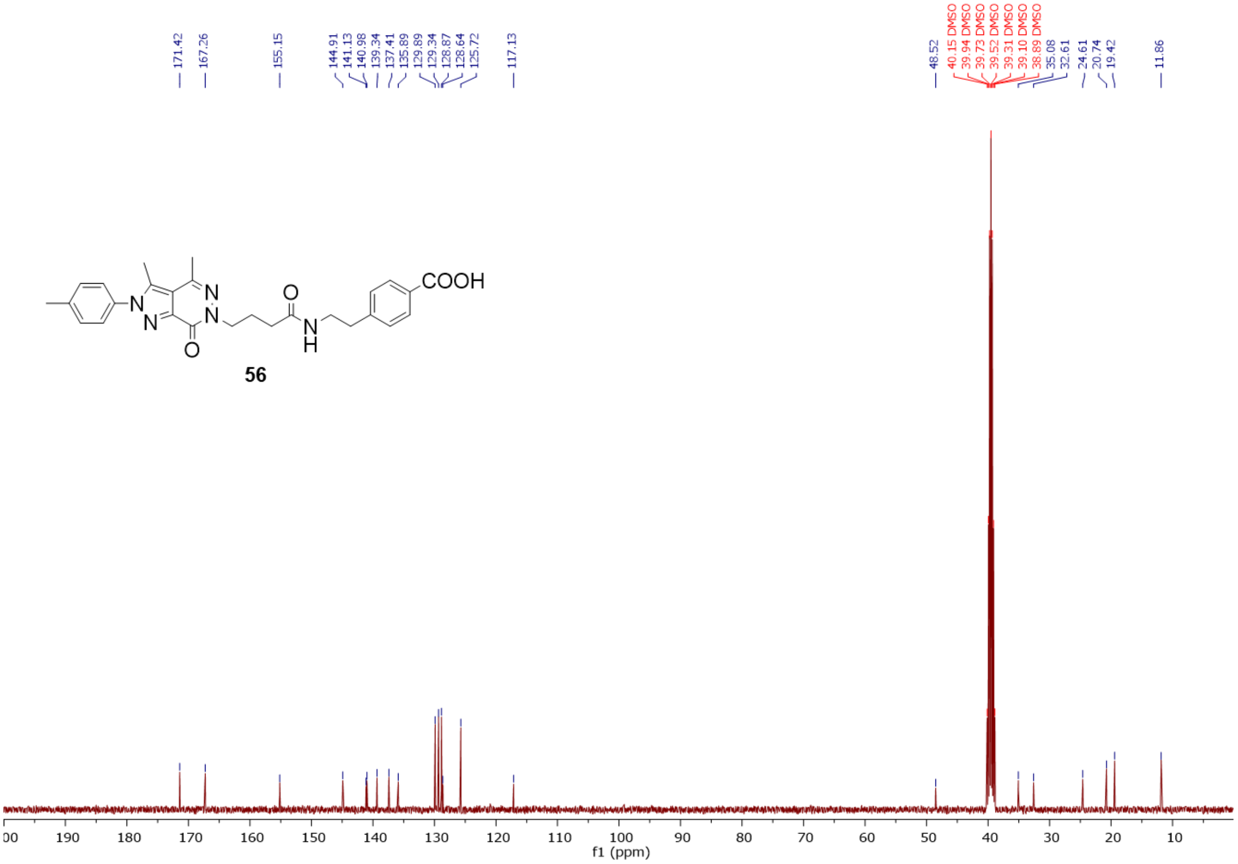
171.42 167.26 155.15 144.91 141.13 140.98 139.34 137.41 135.89 129.89 129.34 128.87 128.64 125.72 117.13
48.52 40.15 DMSO 39.94 DMSO 39.73 DMSO 39.52 DMSO 39.31 DMSO 39.10 DMSO 38.89 DMSO 35.08 32.61 24.61 20.74 19.42 11.86
COOH
56
f1 (ppm)

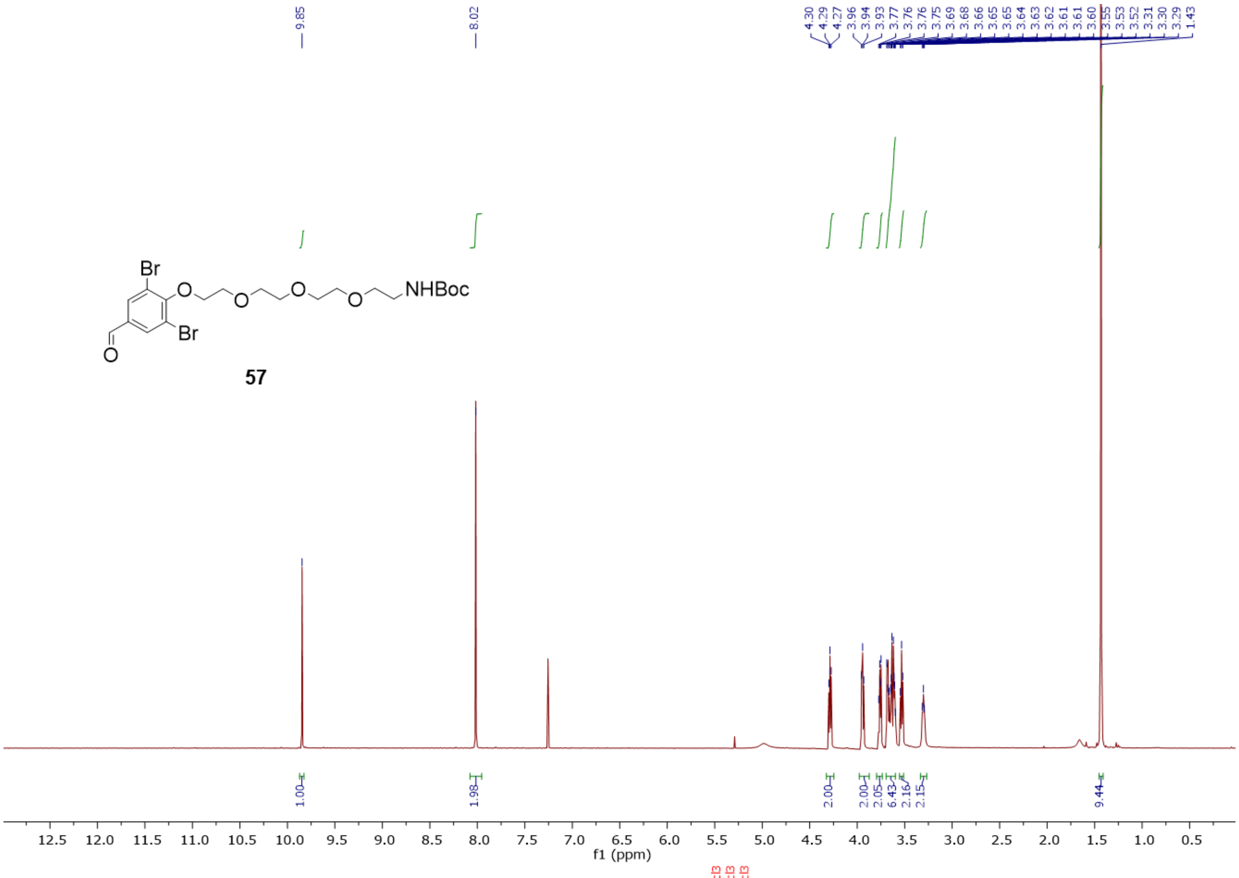

9.85
8.02
4.30 4.29 4.27 3.96 3.94 3.93 3.77 3.76 3.76 3.75 3.69 3.68 3.66 3.65 3.65 3.64 3.63 3.62 3.61 3.61 3.60 3.55 3.53 3.52 3.31 3.30 3.29 1.43
Br
O
O
O
O
NHBoc
Br
O
57
1.00
1.98
2.00
2.00 2.05 6.43 2.16 2.15
9.44
f1 (ppm)

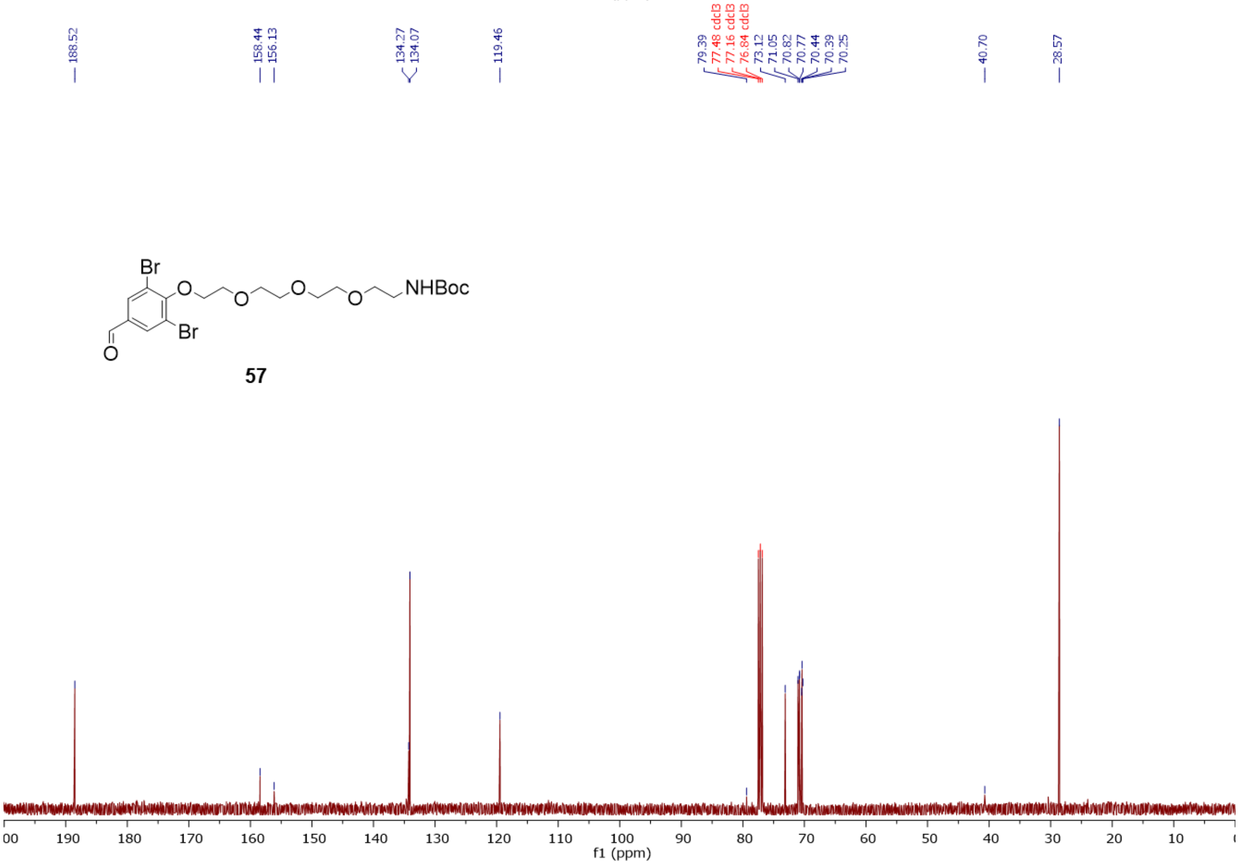

188.52
158.44 156.13
134.27 134.07
119.46
79.39 77.48 cdcl3 77.16 cdcl3 76.84 cdcl3 73.12 71.05 70.82 70.77 70.44 70.39 70.25
40.70
28.57
Br
O
O
O
O
NHBoc
Br
O
57
f1 (ppm)

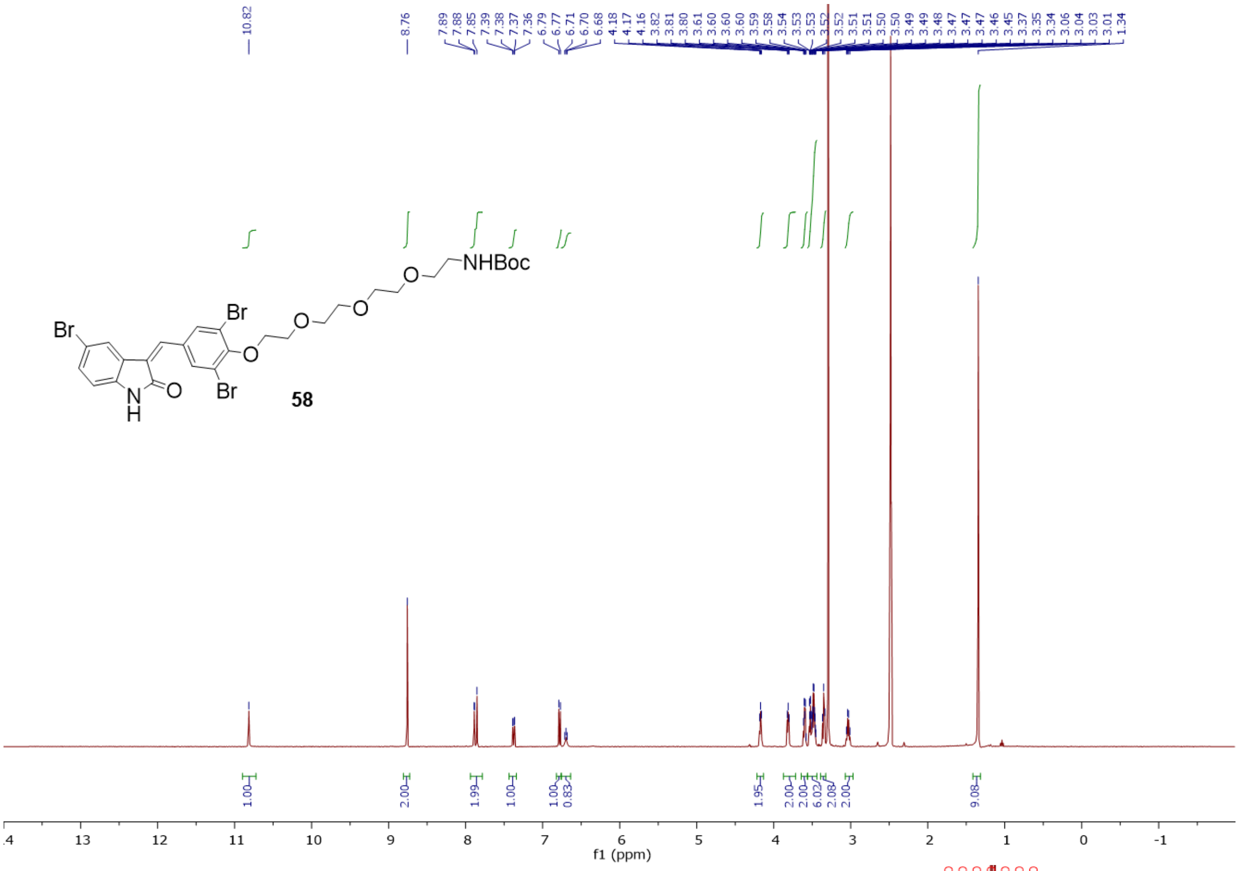
10.82
8.76
1.00
2.00
1.99
1.00
1.00
0.83
1.95
2.00
2.00
6.02
2.08
2.00
9.08
f1 (ppm)
NHBoc
Br
58

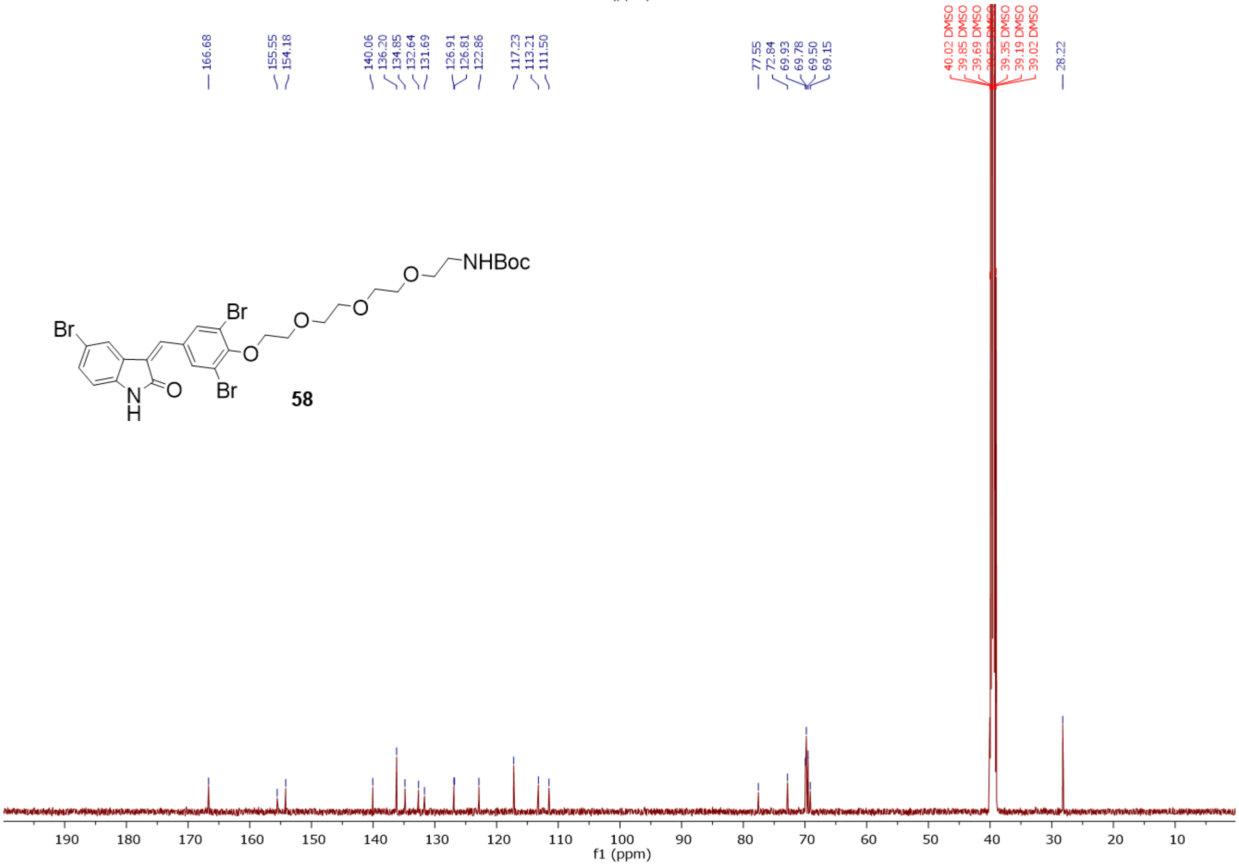
166.68
155.55
154.18
140.06
136.20
134.85
132.64
131.69
126.91
126.81
122.86
117.23
113.21
111.50
77.55
72.84
69.93
69.78
69.50
69.15
28.22
f1 (ppm)
NHBoc
Br
58

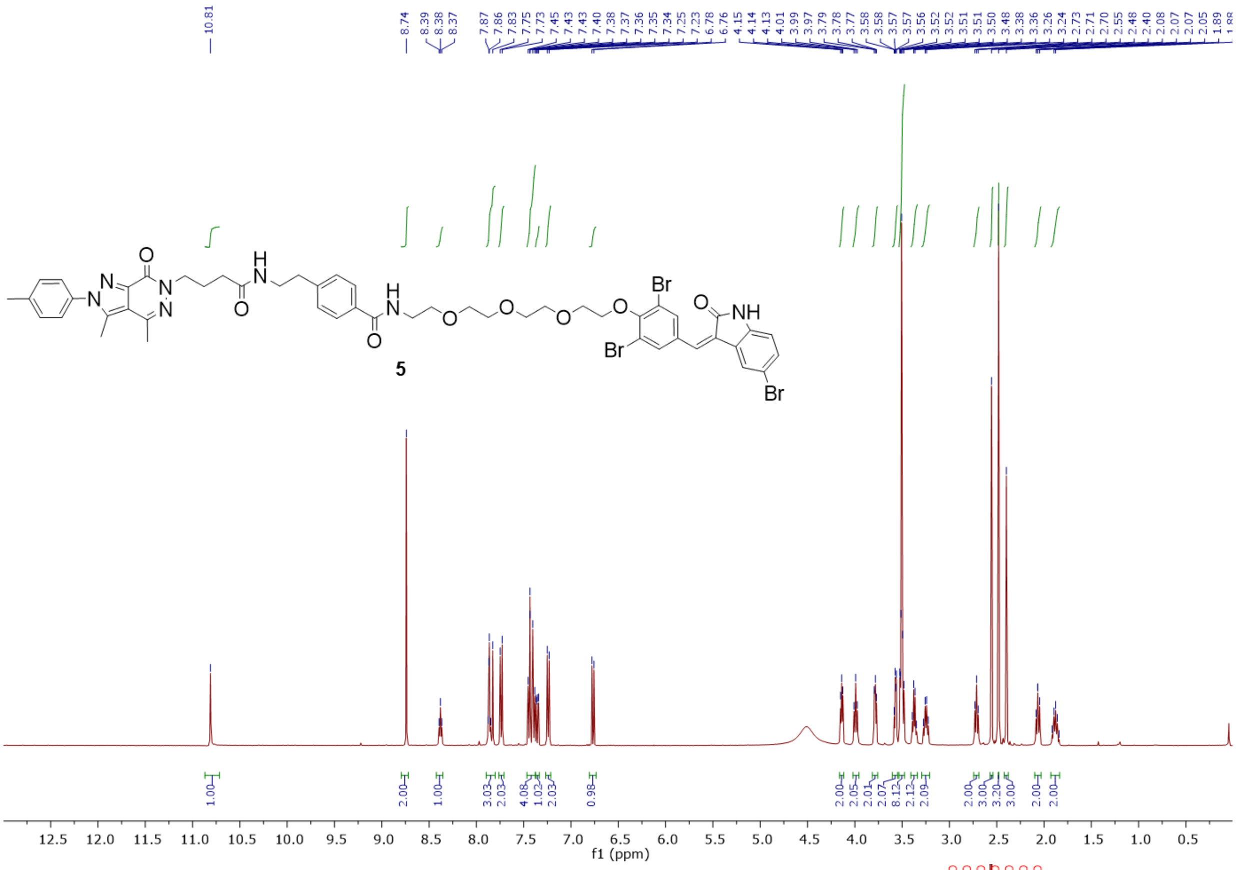
10.81
8.74 8.39 8.38 8.37
7.87 7.86 7.83 7.75 7.73 7.45 7.43 7.43 7.40 7.38 7.37 7.36 7.35 7.34 7.25 7.23 6.78 6.76
4.15 4.14 4.13 4.01 3.99 3.97 3.79 3.78 3.77 3.58 3.58 3.57 3.57 3.56 3.52 3.52 3.51 3.51 3.50 3.48 3.38 3.36 3.26 3.24 2.73 2.71 2.70 2.55 2.48 2.40 2.08 2.07 2.07 2.05 1.89
N
N
O
N
N
N
H
N
O
H
N
O
O
O
O
O
Br
Br
O
NH
Br
5
1.00
2.00
1.00
3.03
2.03
4.08
1.02
2.03
0.98
2.00
2.05
2.01
2.07
8.12
2.12
2.09
2.00
3.00
3.20
3.00
2.00
2.00
12.5 12.0 11.5 11.0 10.5 10.0 9.5 9.0 8.5 8.0 7.5 7.0 6.5 6.0 5.5 5.0 4.5 4.0 3.5 3.0 2.5 2.0 1.5 1.0 0.5
f1 (ppm)

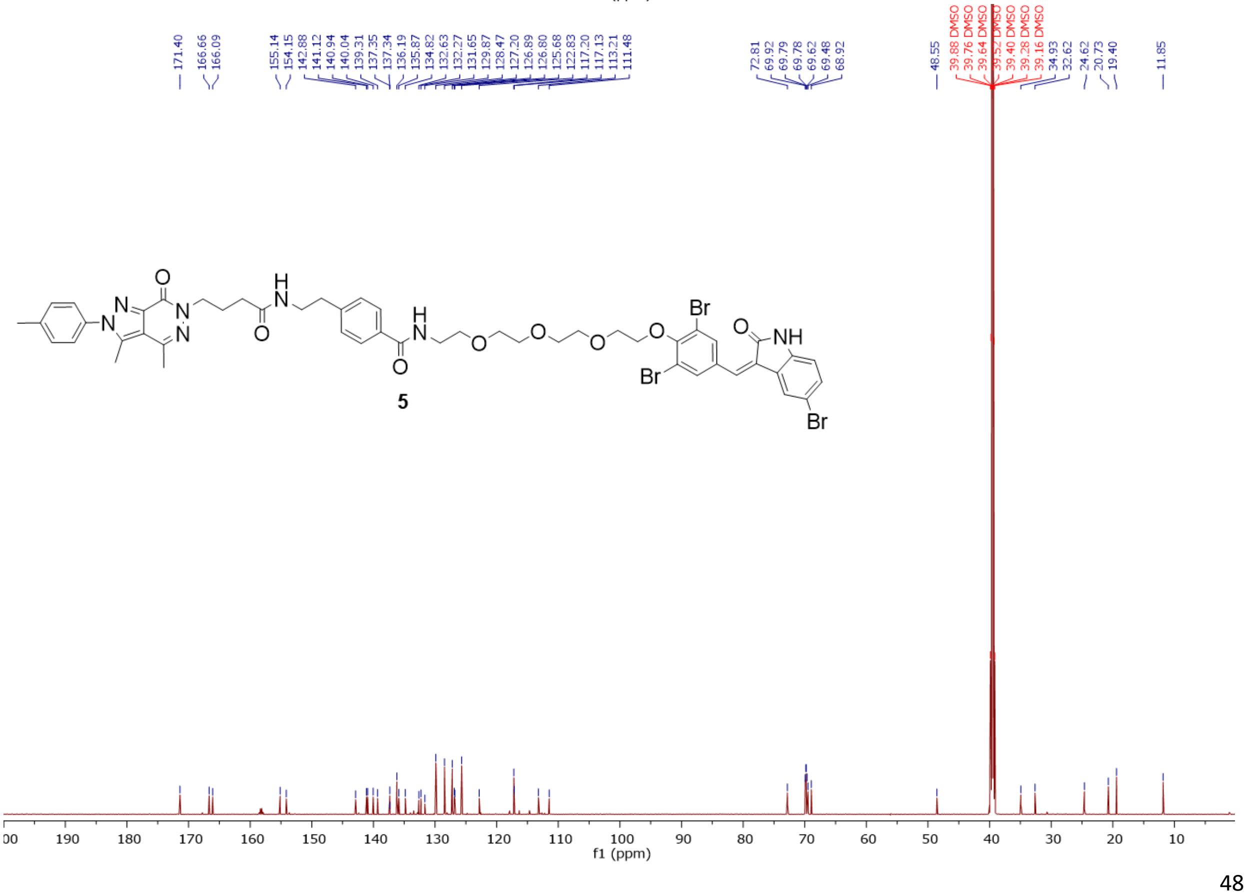
171.40
166.66 166.09
155.14 154.15 142.88 141.12 140.94 140.04 139.31 137.35 137.34 136.19 135.87 134.82 132.63 132.27 131.65 129.87 128.47 127.20 126.89 126.80 125.68 122.83 117.20 117.13 113.21 111.48
72.81 69.92 69.79 69.78 69.62 69.48 68.92
48.55
39.88 DMSO 39.76 DMSO 39.64 DMSO 39.52 DMSO 39.40 DMSO 39.28 DMSO 39.16 DMSO
34.93 32.62 24.62 20.73 19.40 11.85
N
N
O
N
N
N
H
N
O
H
N
O
O
O
O
O
Br
Br
O
NH
Br
5
00 190 180 170 160 150 140 130 120 110 100 90 80 70 60 50 40 30 20 10
f1 (ppm)

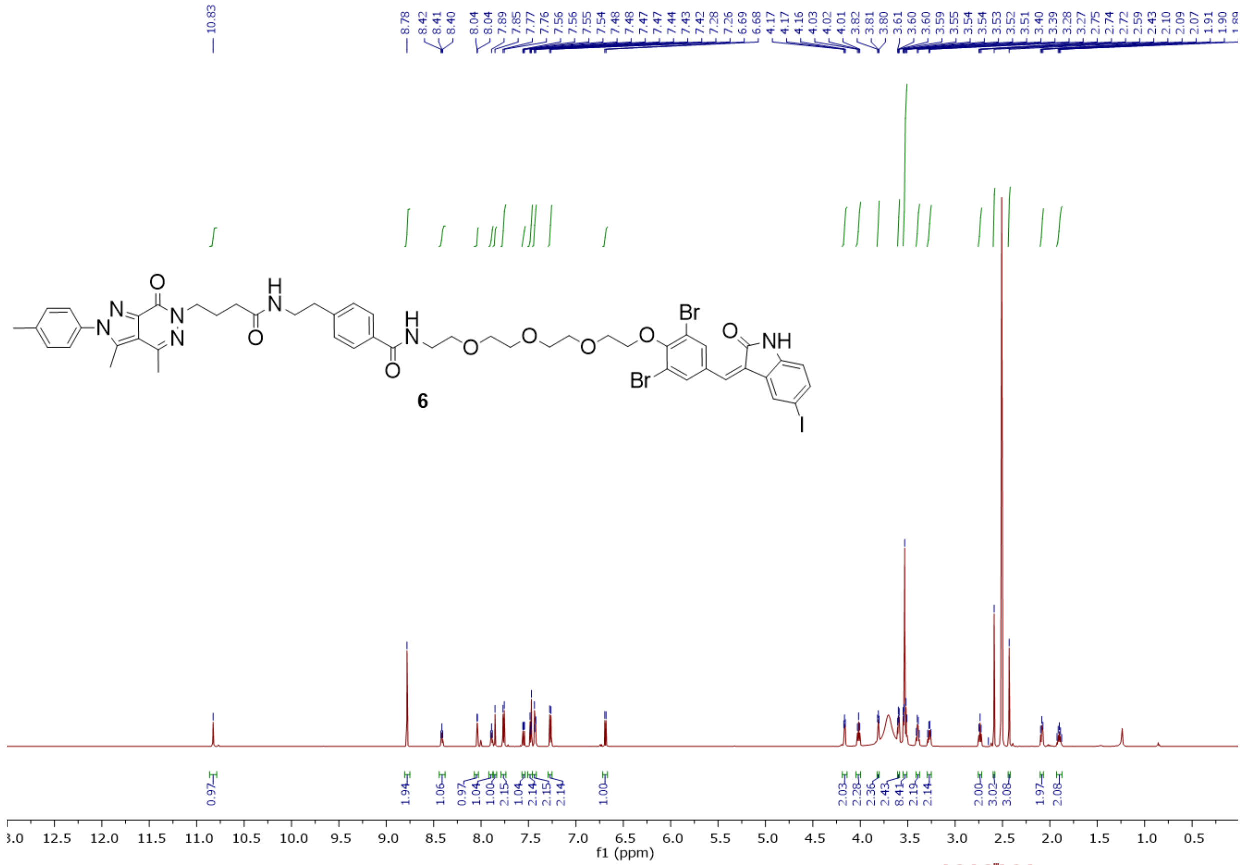
6
f1 (ppm)

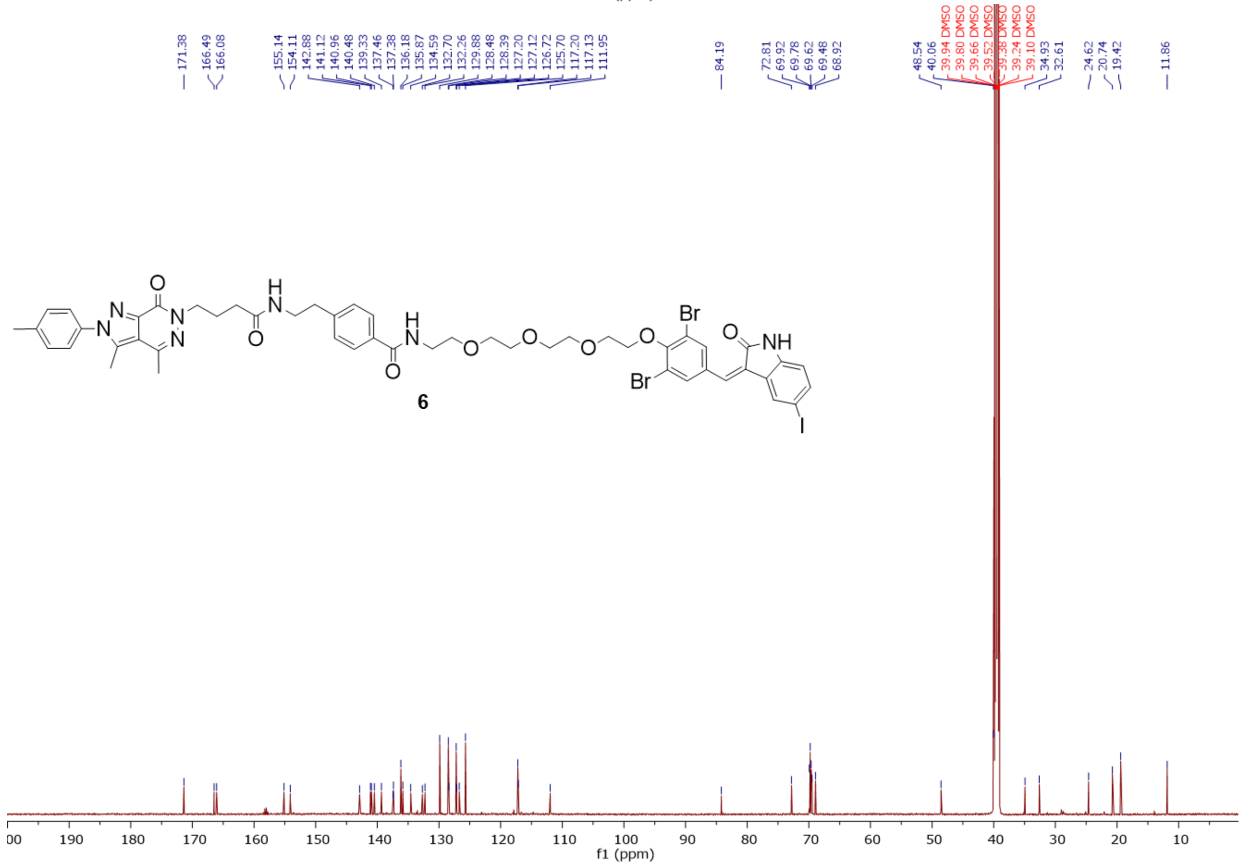
6
f1 (ppm)

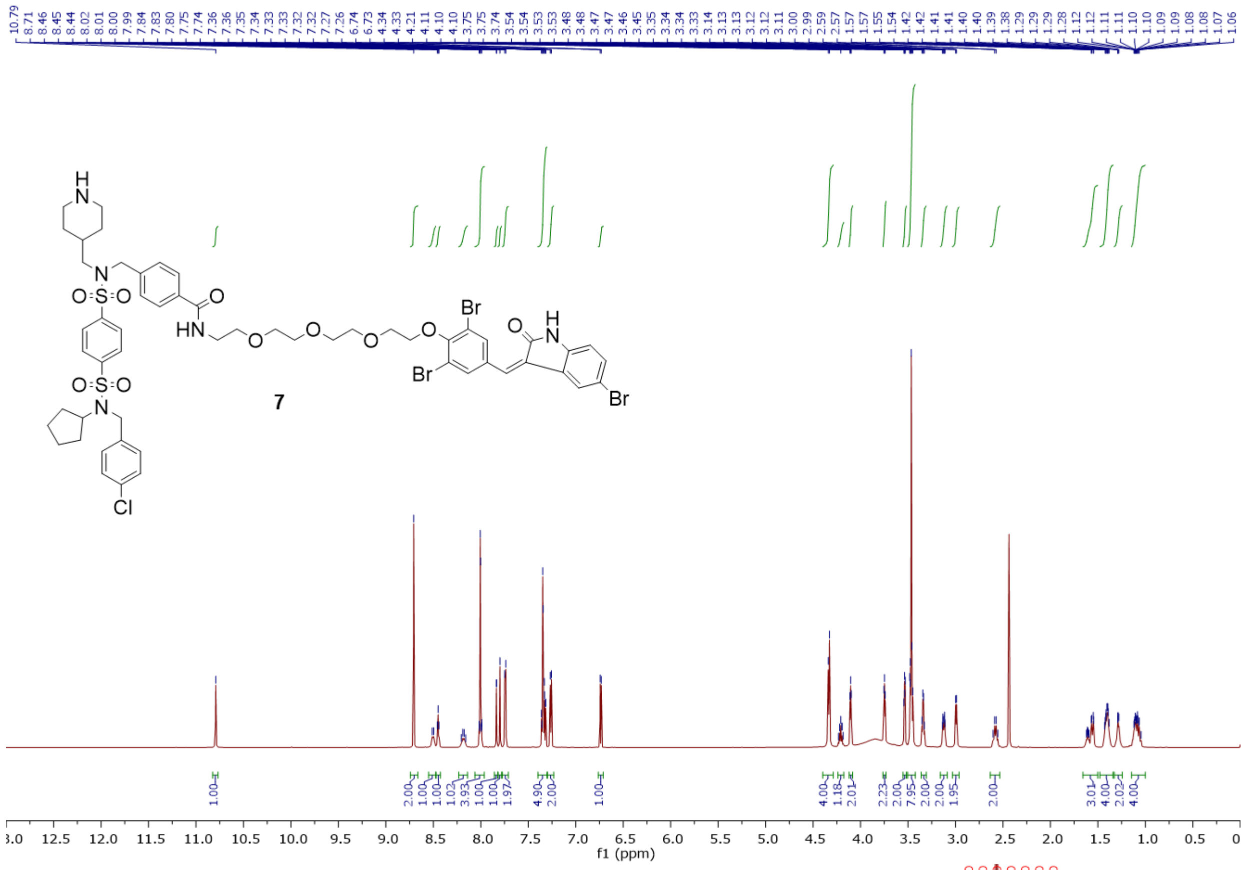

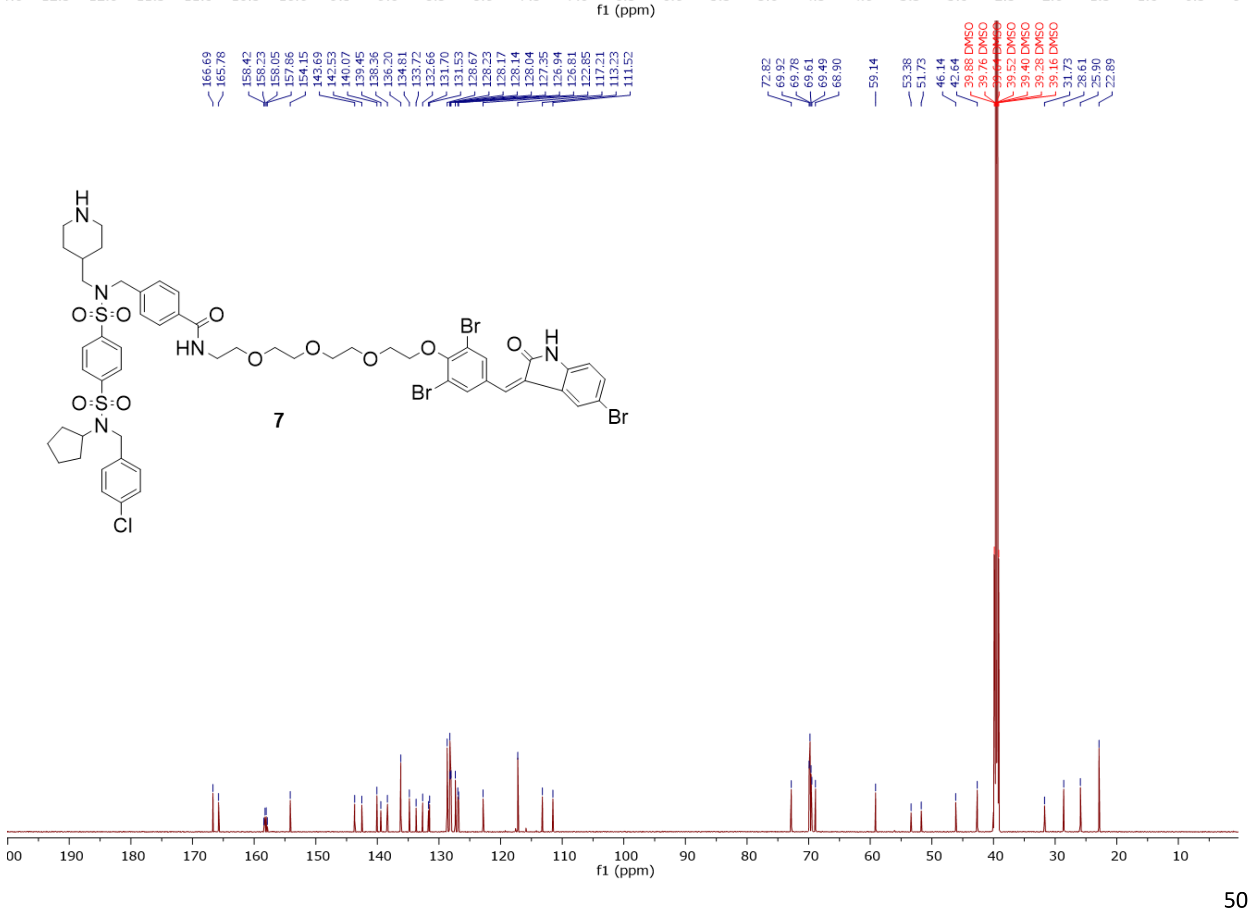

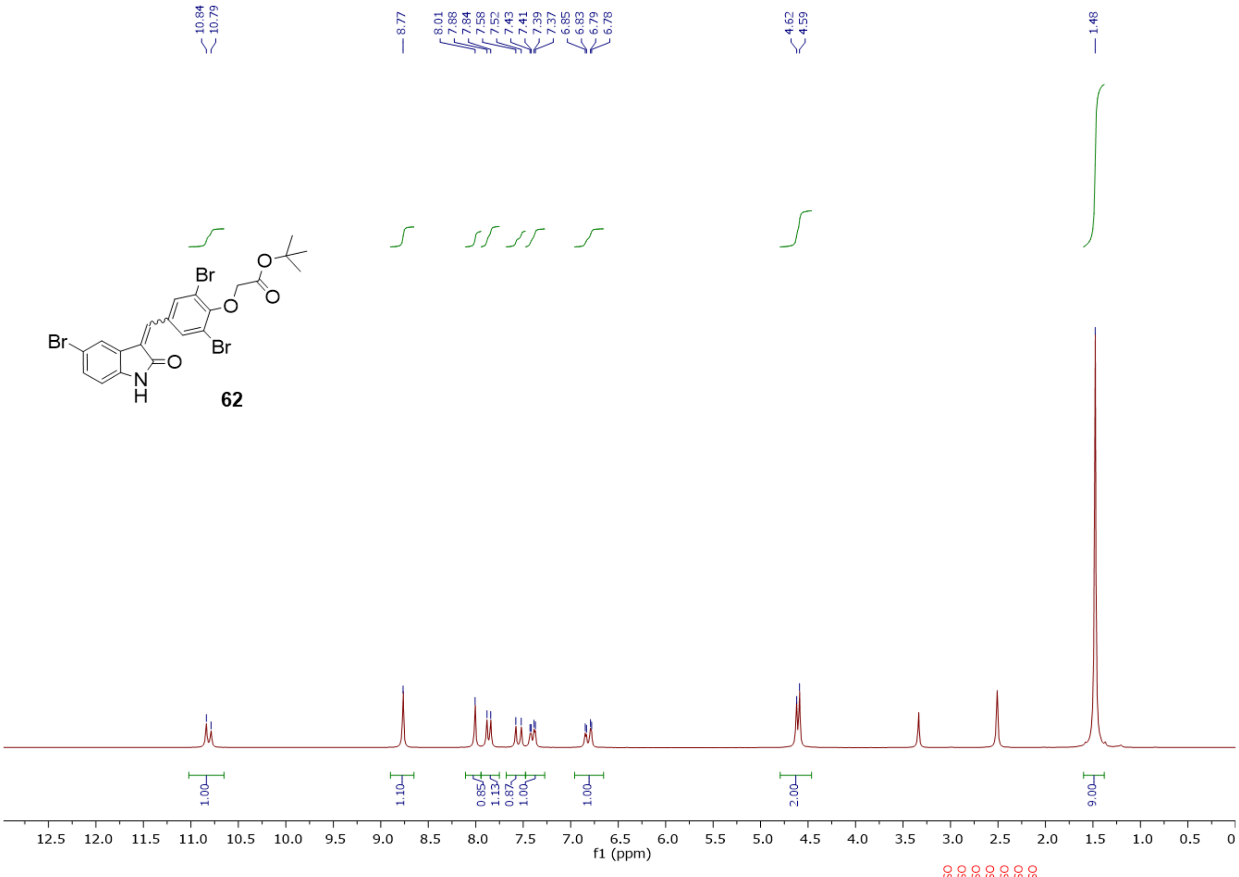
10.84
10.79
8.77
8.01
7.88
7.84
7.58
7.52
7.43
7.41
7.39
7.37
6.85
6.83
6.79
6.78
4.62
4.59
1.48
Br
Br
Br
O
O
O
O
N
H
62
1.00
1.10
0.85
1.13
0.87
1.00
1.00
2.00
9.00
f1 (ppm)

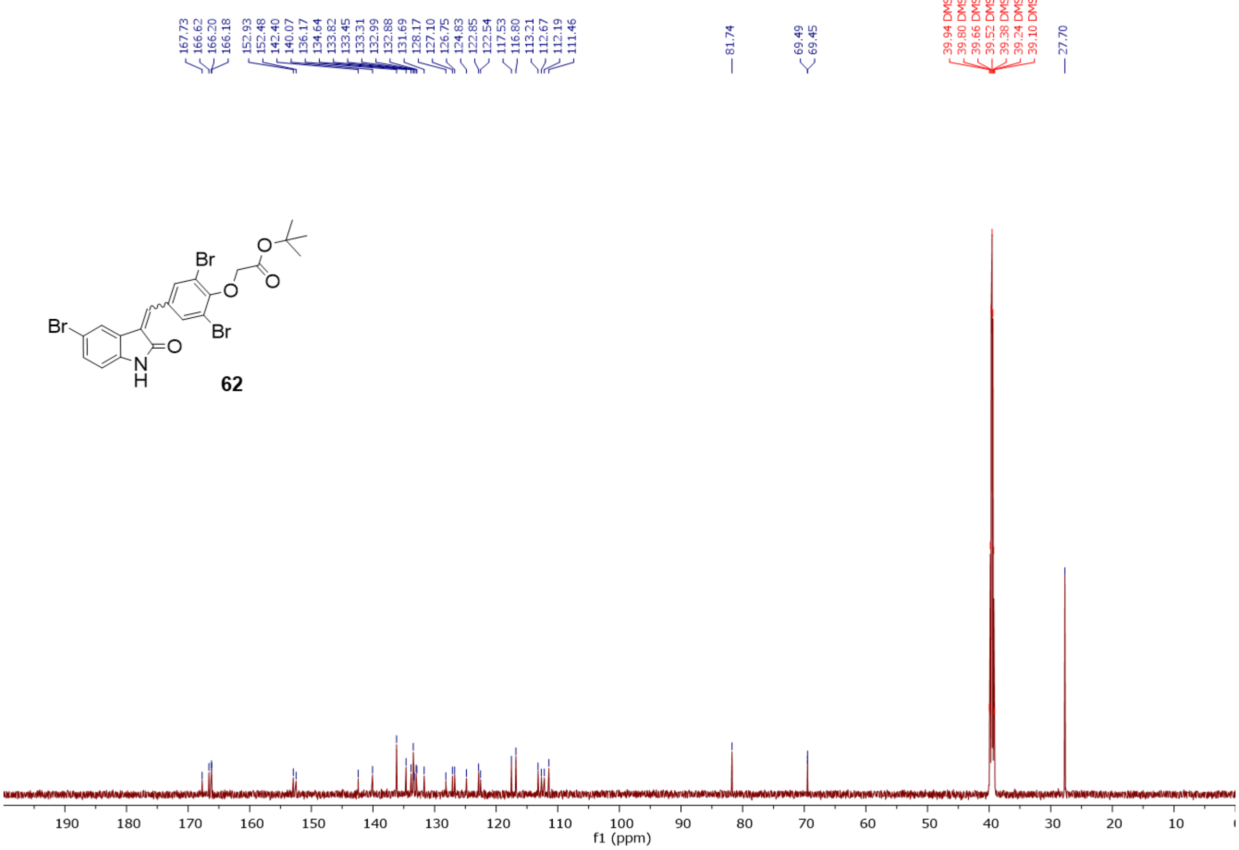
167.73
166.62
166.20
166.18
152.93
152.48
142.40
140.07
136.17
134.64
133.82
133.45
133.31
132.99
132.88
131.69
128.17
127.10
126.75
124.83
122.85
122.54
117.53
116.80
113.21
112.67
112.19
111.46
81.74
69.49
69.45
39.94 DMSO
39.80 DMSO
39.66 DMSO
39.52 DMSO
39.38 DMSO
39.24 DMSO
39.10 DMSO
27.70
Br
Br
Br
O
O
O
O
N
H
62
f1 (ppm)

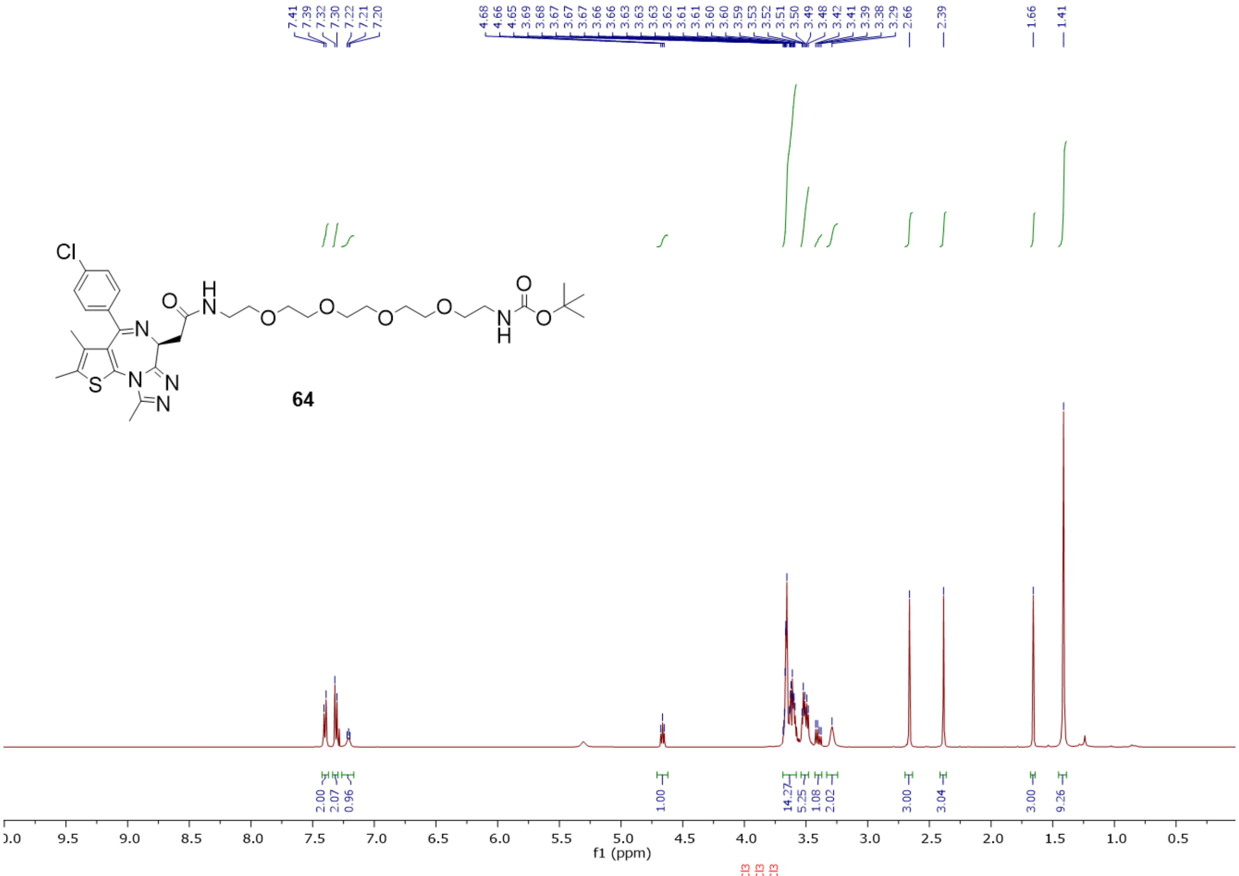
7.41 7.39 7.32 7.30 7.22 7.21 7.20
4.68 4.66 4.65 3.69 3.68 3.67 3.67 3.66 3.66 3.63 3.63 3.62 3.61 3.61 3.60 3.60 3.59 3.53 3.52 3.51 3.50 3.49 3.48 3.42 3.41 3.39 3.38 3.29
2.66
2.39
1.66
1.41
Cl
O
H
N
O
N
S
N
N
N
64
2.00 2.07 0.96
1.00
14.27 5.25 1.08 2.02
3.00
3.04
3.00
9.26
f1 (ppm)

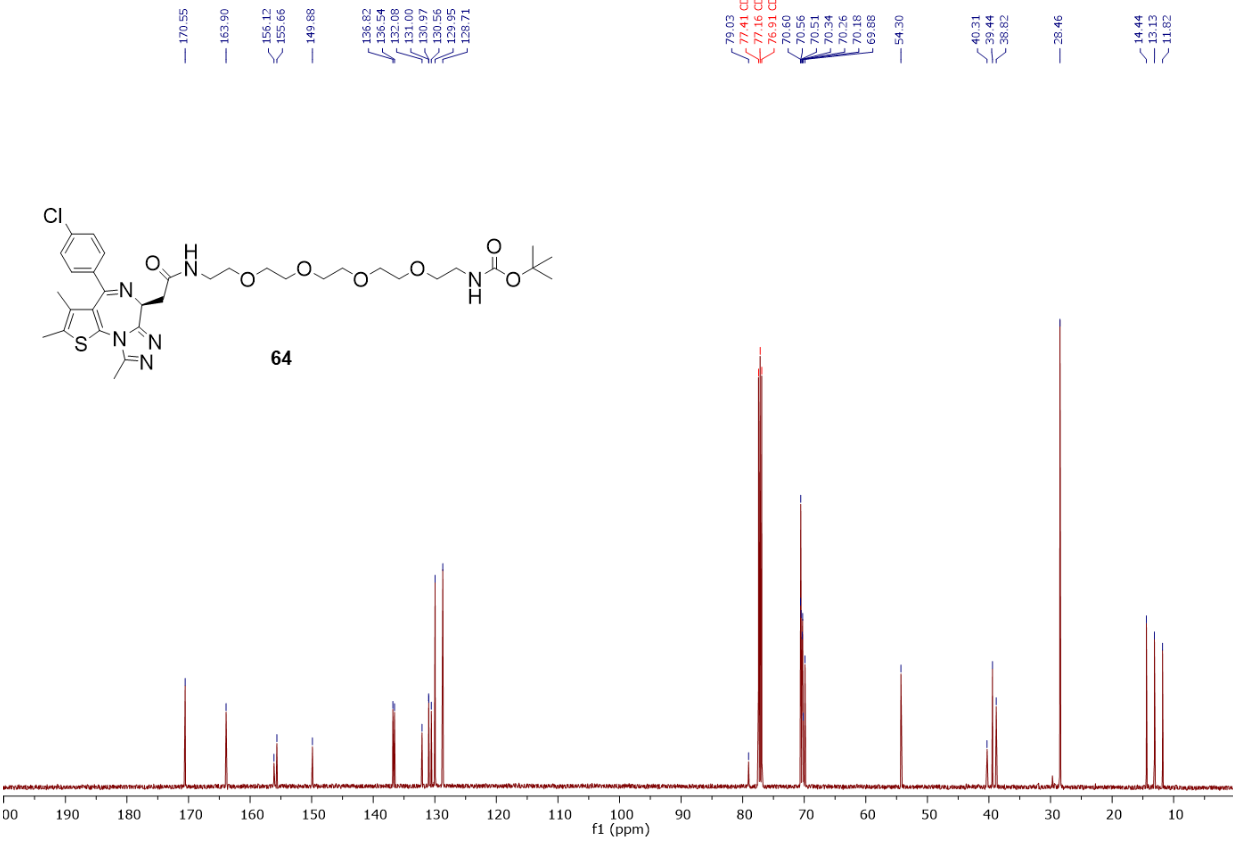
170.55
163.90
156.12 155.66
149.88
136.82 136.54 132.08 131.00 130.97 130.56 129.95 128.71
79.03
77.41 CDCl3
77.16 CDCl3
76.91 CDCl3
70.60 70.56 70.51 70.34 70.26 70.18 69.88
54.30
40.31 39.44 38.82
28.46
14.44 13.13 11.82
Cl
64
f1 (ppm)

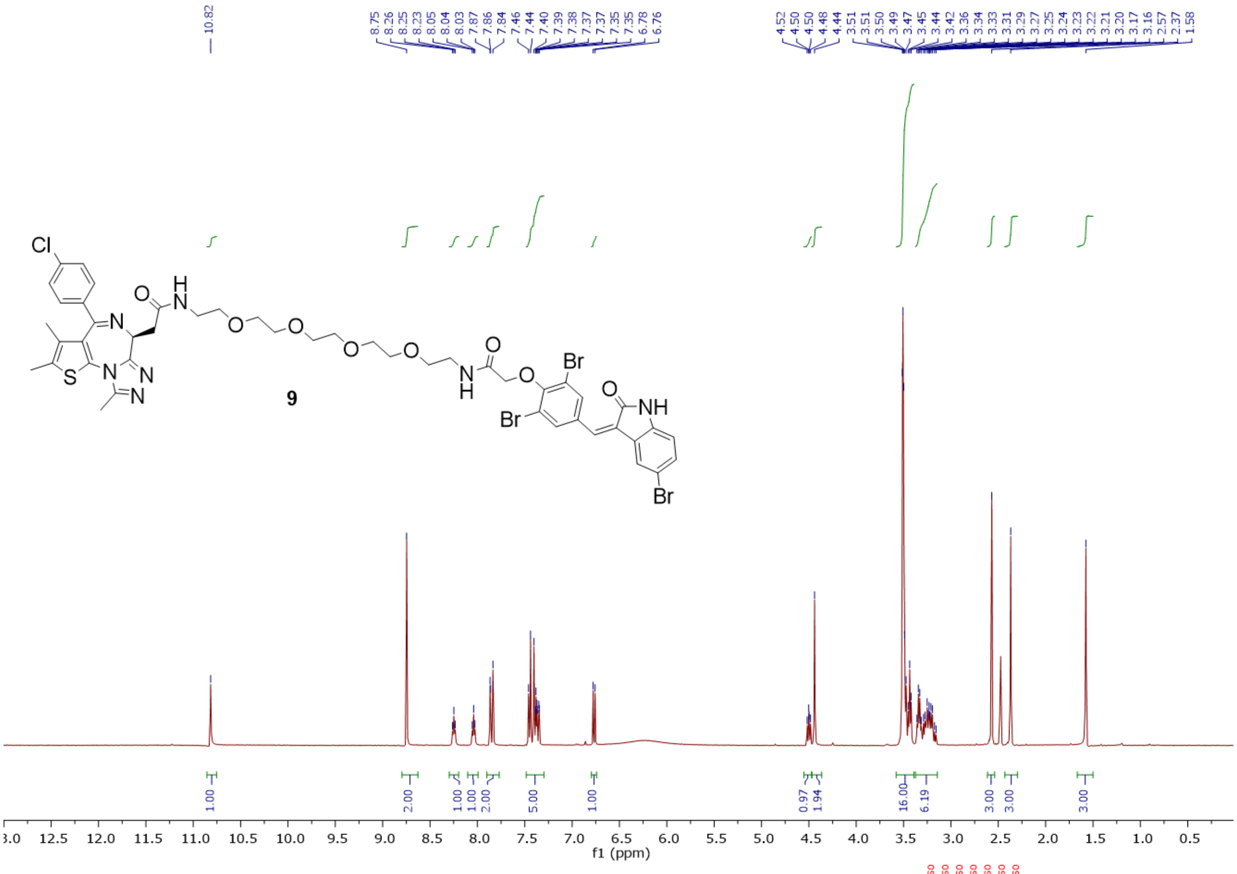
10.82
8.75 8.26 8.25 8.23 8.05 8.04 8.03 7.87 7.86 7.84 7.46 7.44 7.40 7.39 7.38 7.37 7.37 7.35 7.35 6.78 6.76
4.52 4.50 4.50 4.48 4.44 3.51 3.51 3.50 3.49 3.47 3.45 3.44 3.42 3.36 3.34 3.33 3.31 3.29 3.27 3.25 3.24 3.23 3.22 3.21 3.20 3.17 3.16 2.57 2.37 1.58
9
1.00 2.00 1.00 1.00 2.00 5.00 1.00 0.97 1.94 16.00 6.19 3.00 3.00 3.00
f1 (ppm)

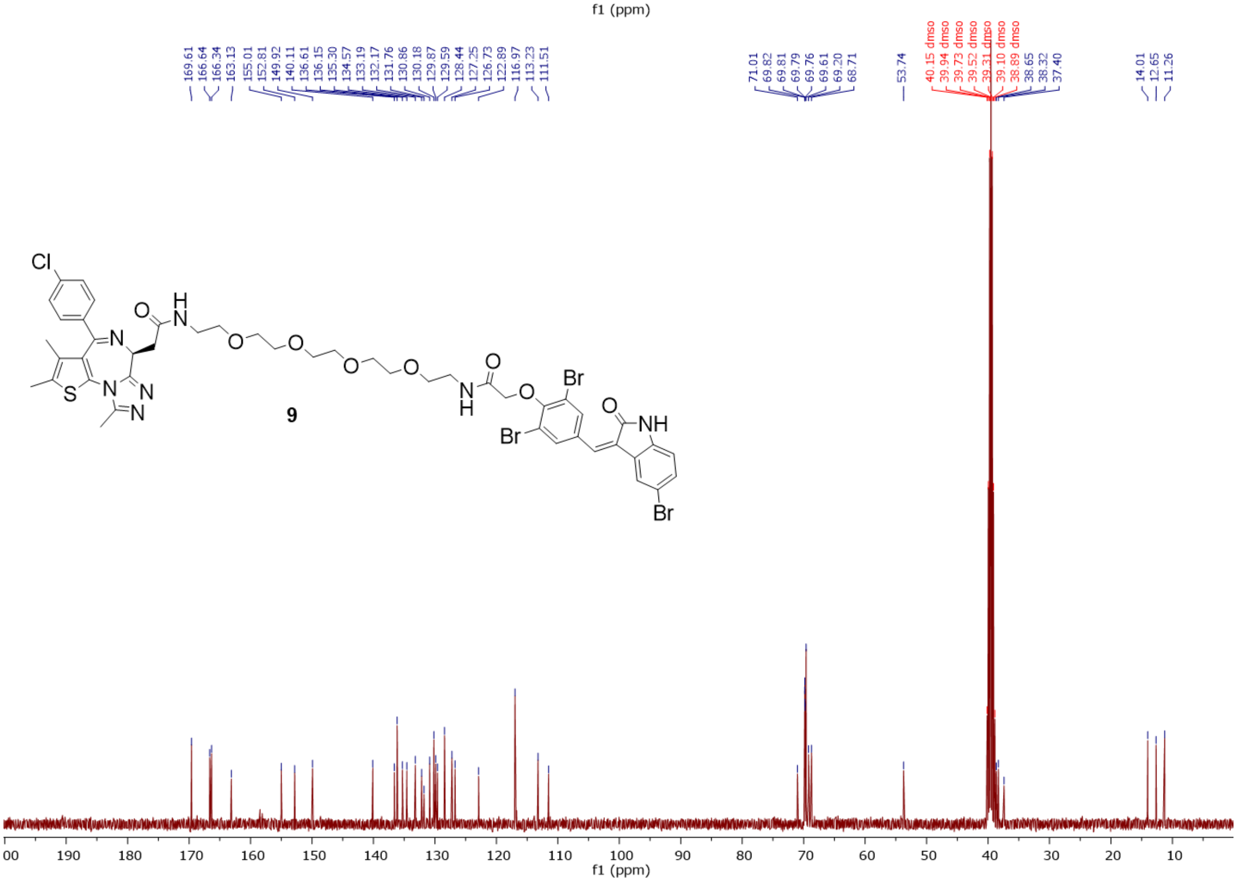
169.61 166.64 166.34 163.13 155.01 152.81 149.92 140.11 136.61 136.15 135.30 134.57 133.19 132.17 131.76 130.86 130.18 129.87 129.59 128.44 127.25 126.73 122.89 116.97 113.23 111.51
71.01 69.82 69.81 69.79 69.76 69.61 69.20 68.71
53.74
40.15 dmso 39.94 dmso 39.73 dmso 39.52 dmso 39.31 dmso 39.10 dmso 38.89 dmso 38.65 38.32 37.40
14.01 12.65 11.26
9
f1 (ppm)

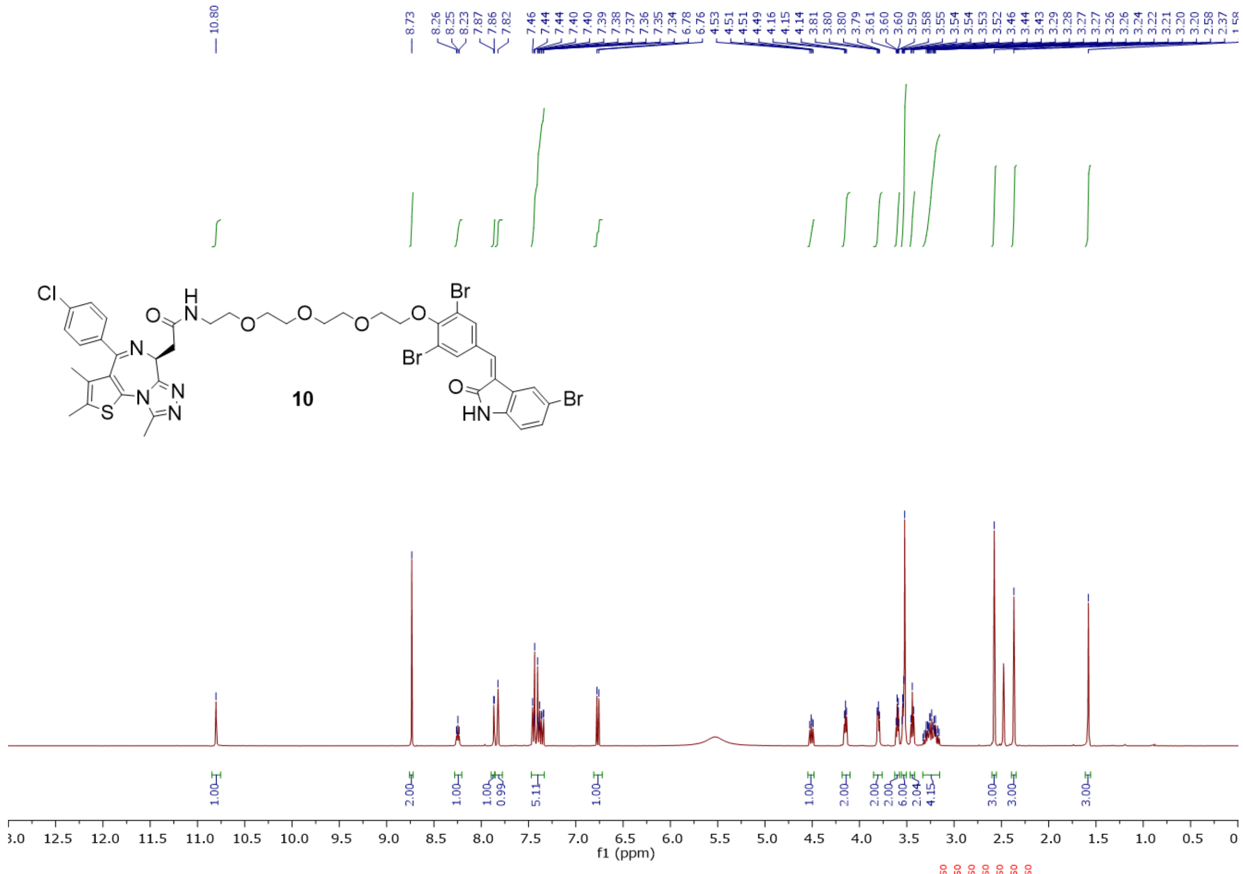
10
f1 (ppm)

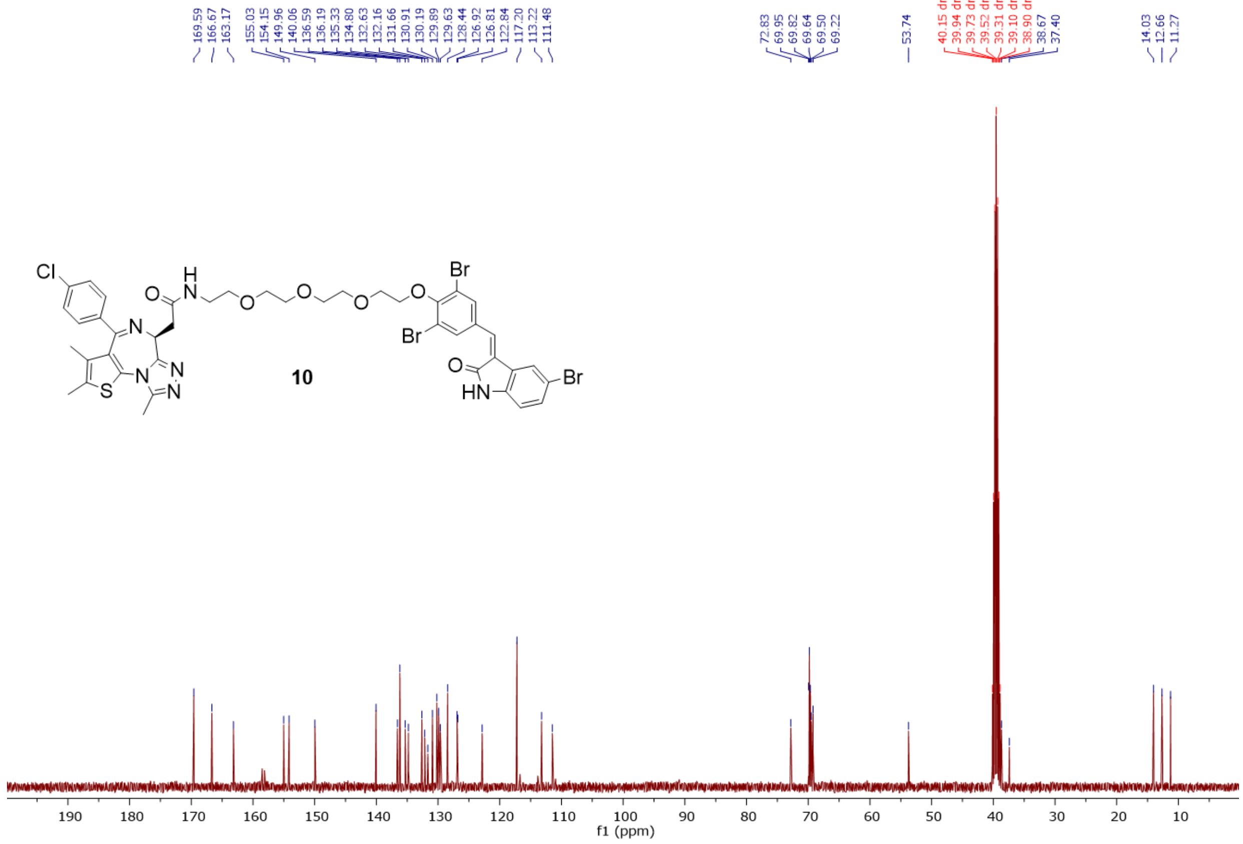
10
f1 (ppm)

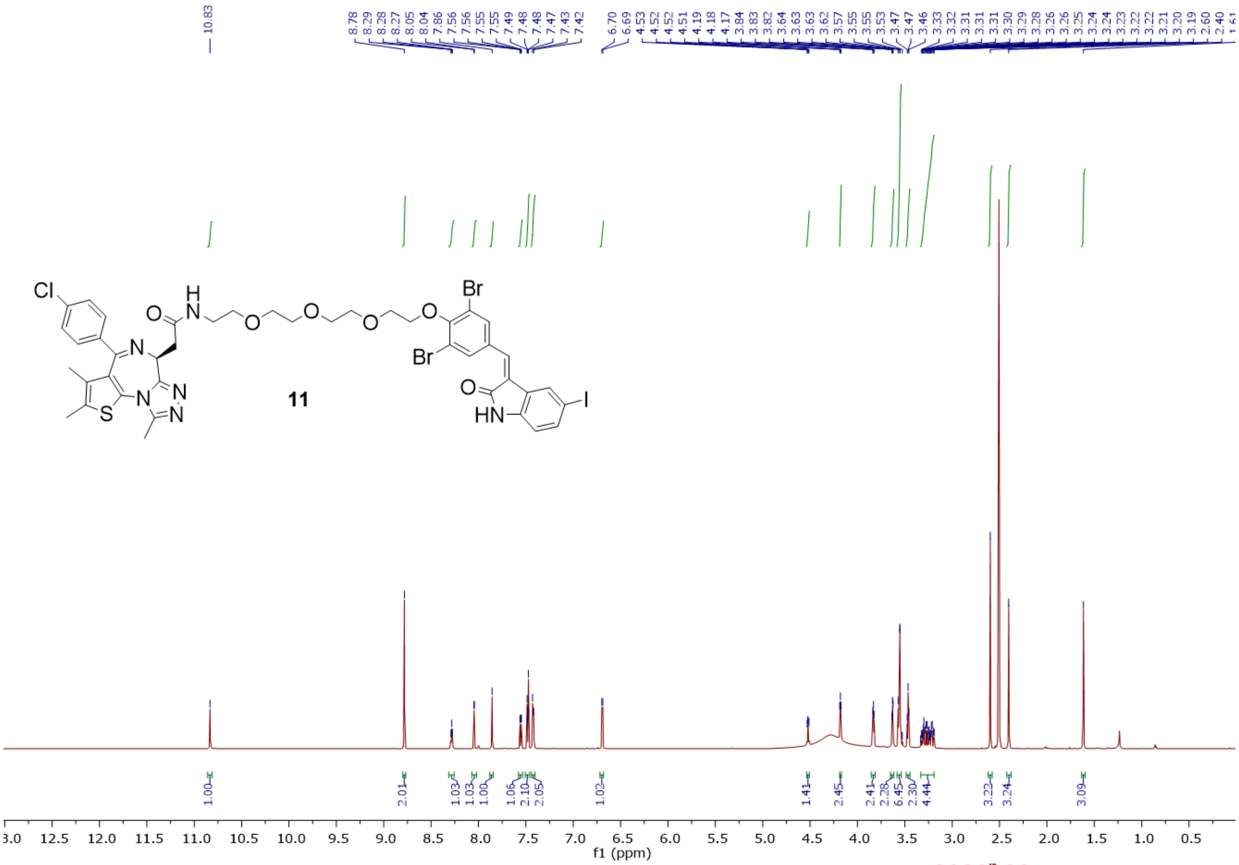

Cl
H
N
O
O
O
O
Br
Br
N
S
N
N
N
11
O
HN
I
f1 (ppm)

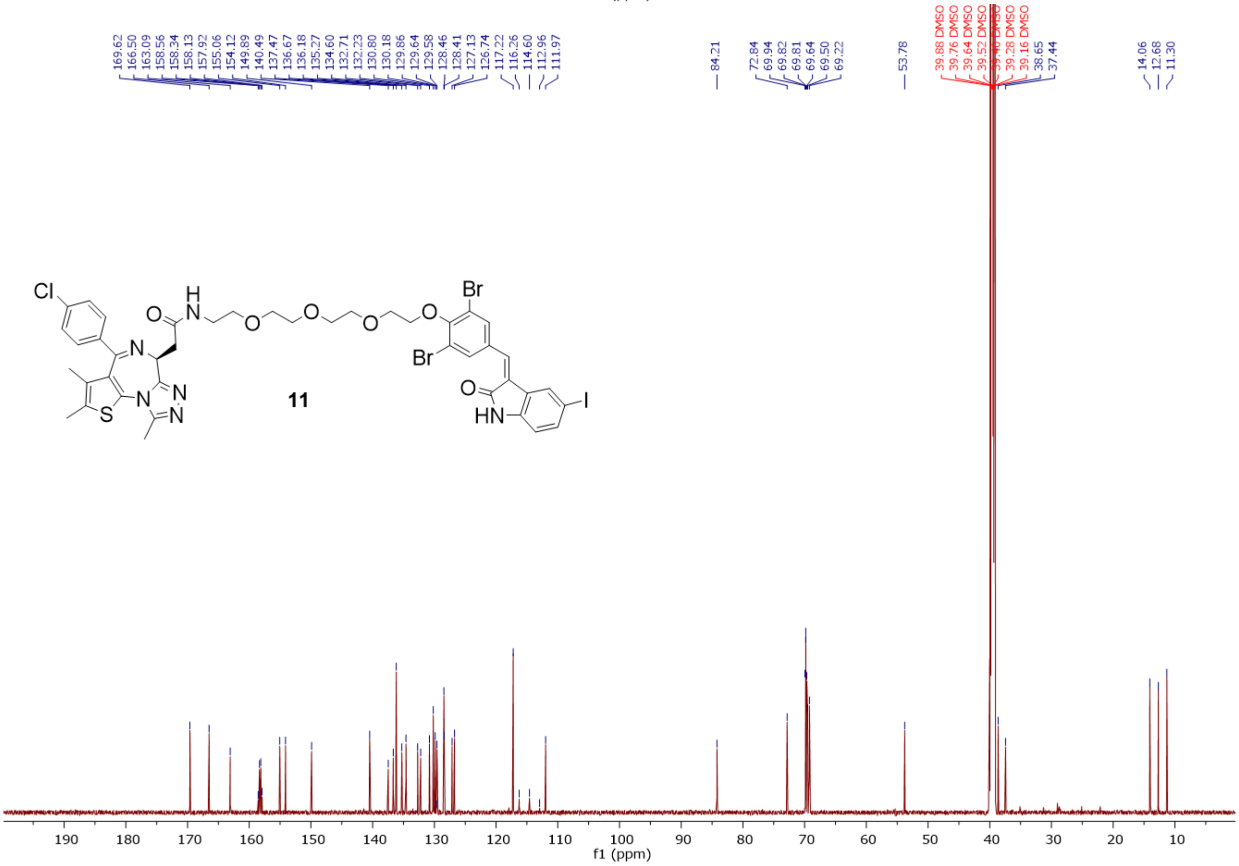

Cl
H
N
O
O
O
O
Br
Br
N
S
N
N
N
11
O
HN
I
f1 (ppm)

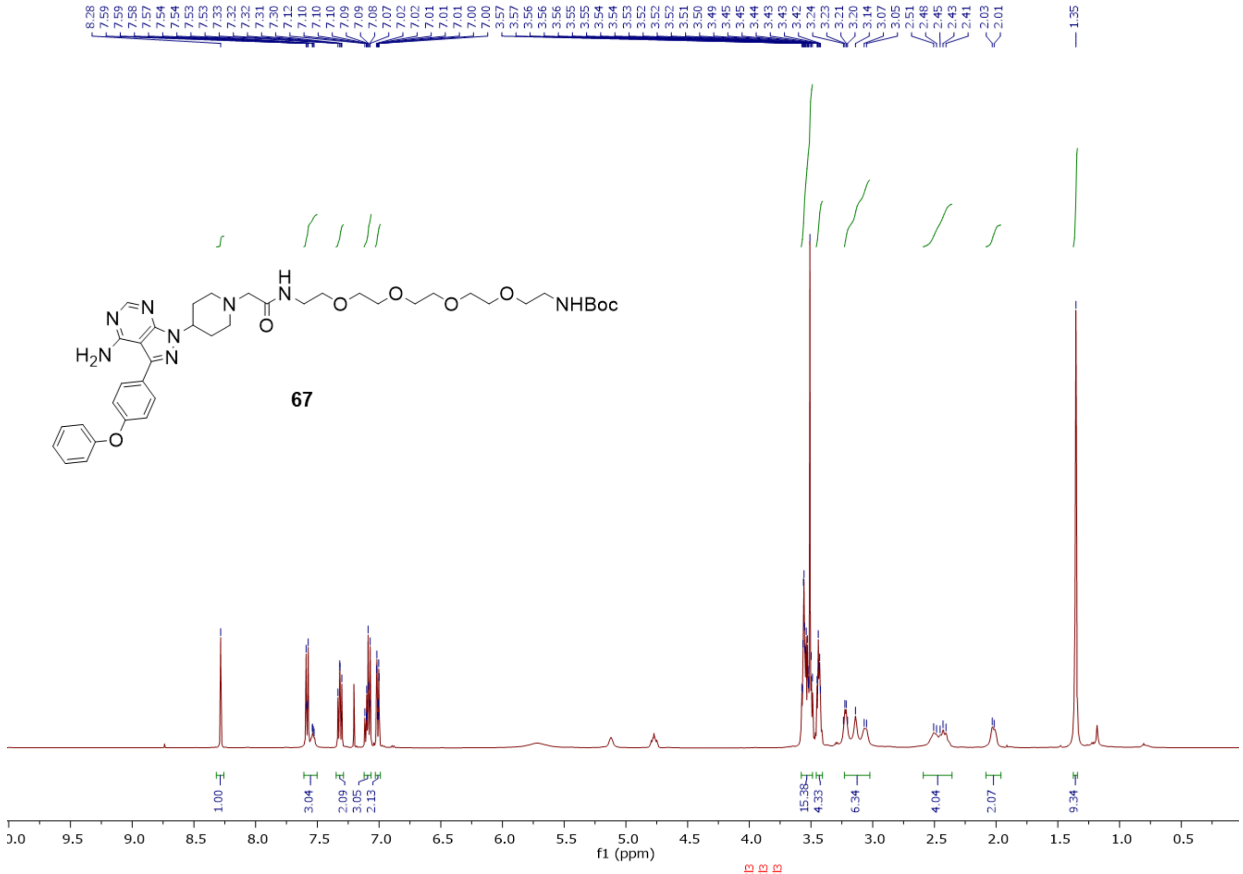

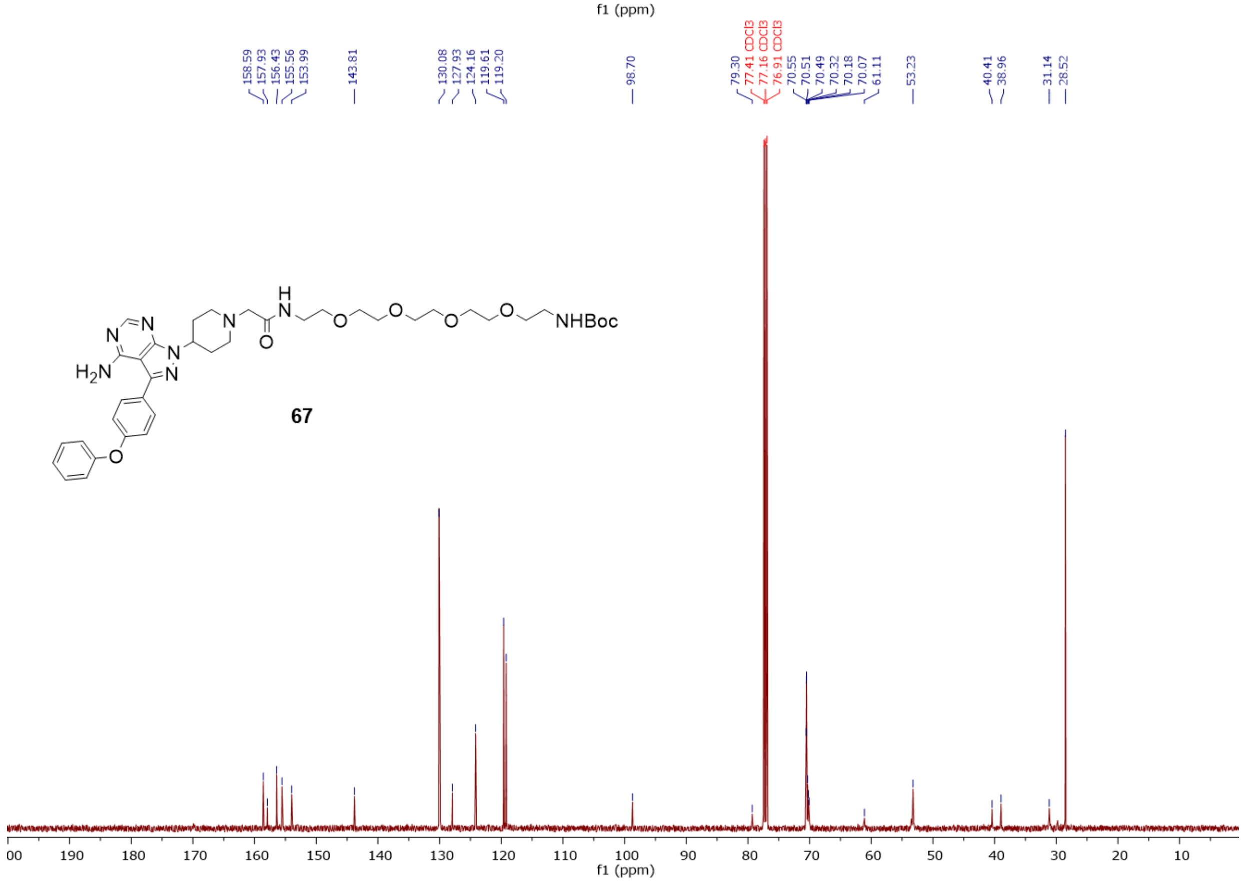

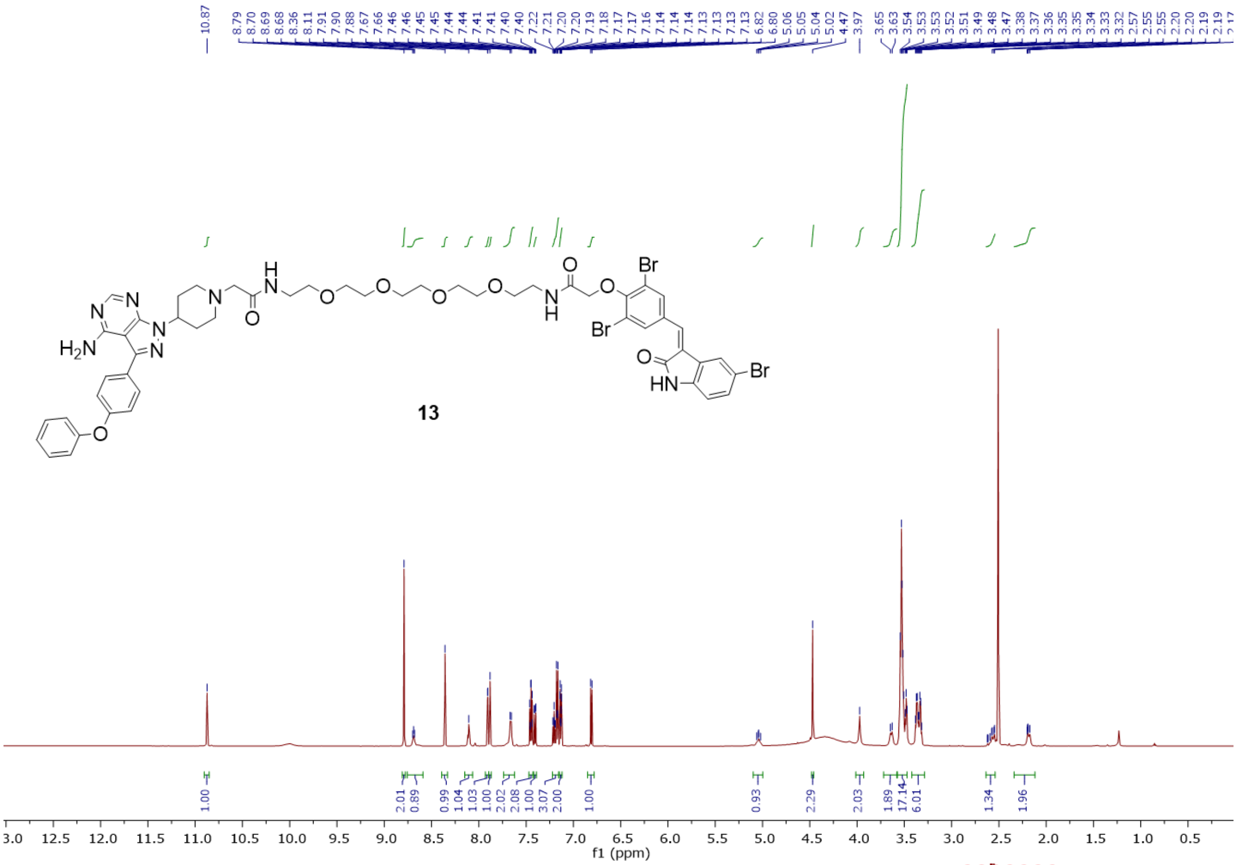
13
f1 (ppm)

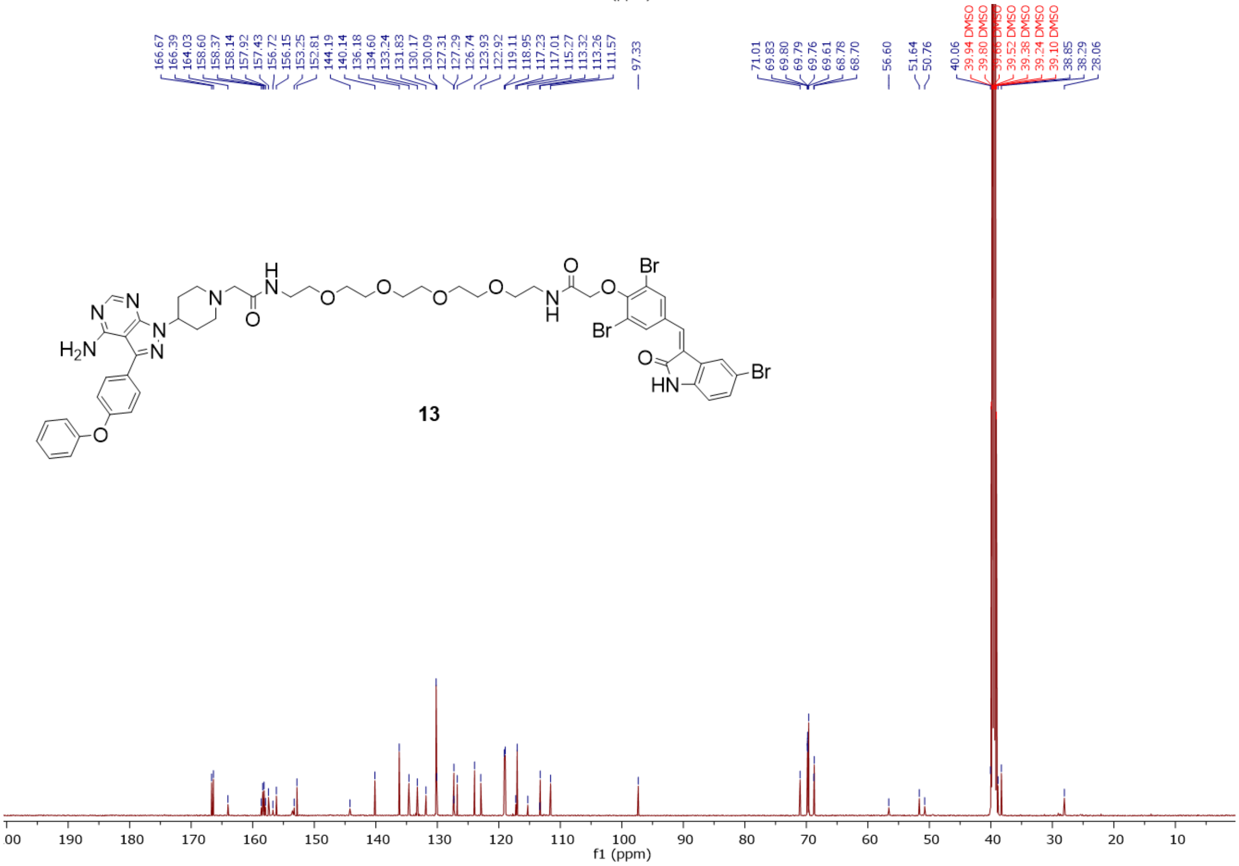
13
f1 (ppm)

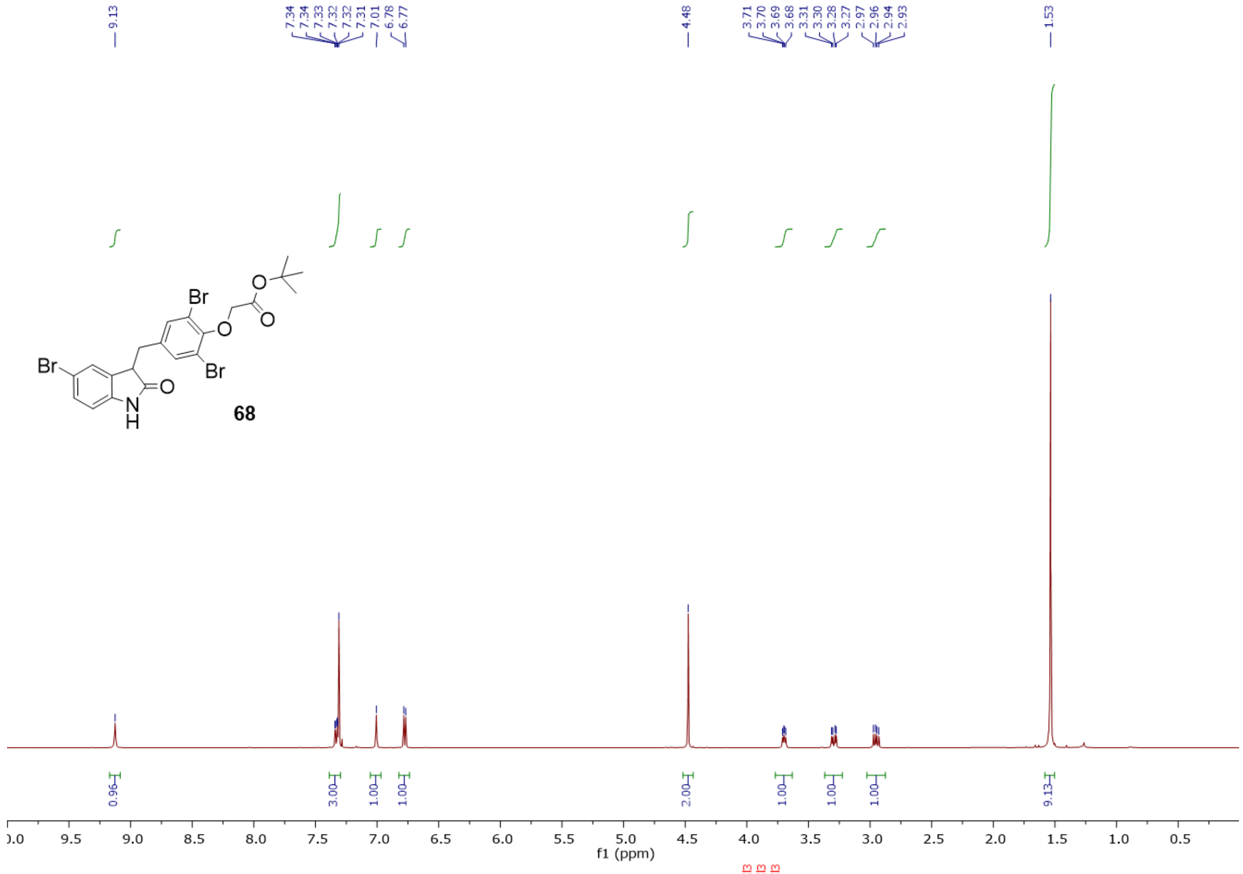
9.13
7.34
7.34
7.33
7.32
7.32
7.31
7.01
6.78
6.77
4.48
3.71
3.70
3.69
3.68
3.31
3.30
3.28
3.27
2.97
2.96
2.94
2.93
1.53
Br
O
O
O
Br
Br
N
H
O
68
0.96
3.00
1.00
1.00
2.00
1.00
1.00
1.00
9.13
f1 (ppm)

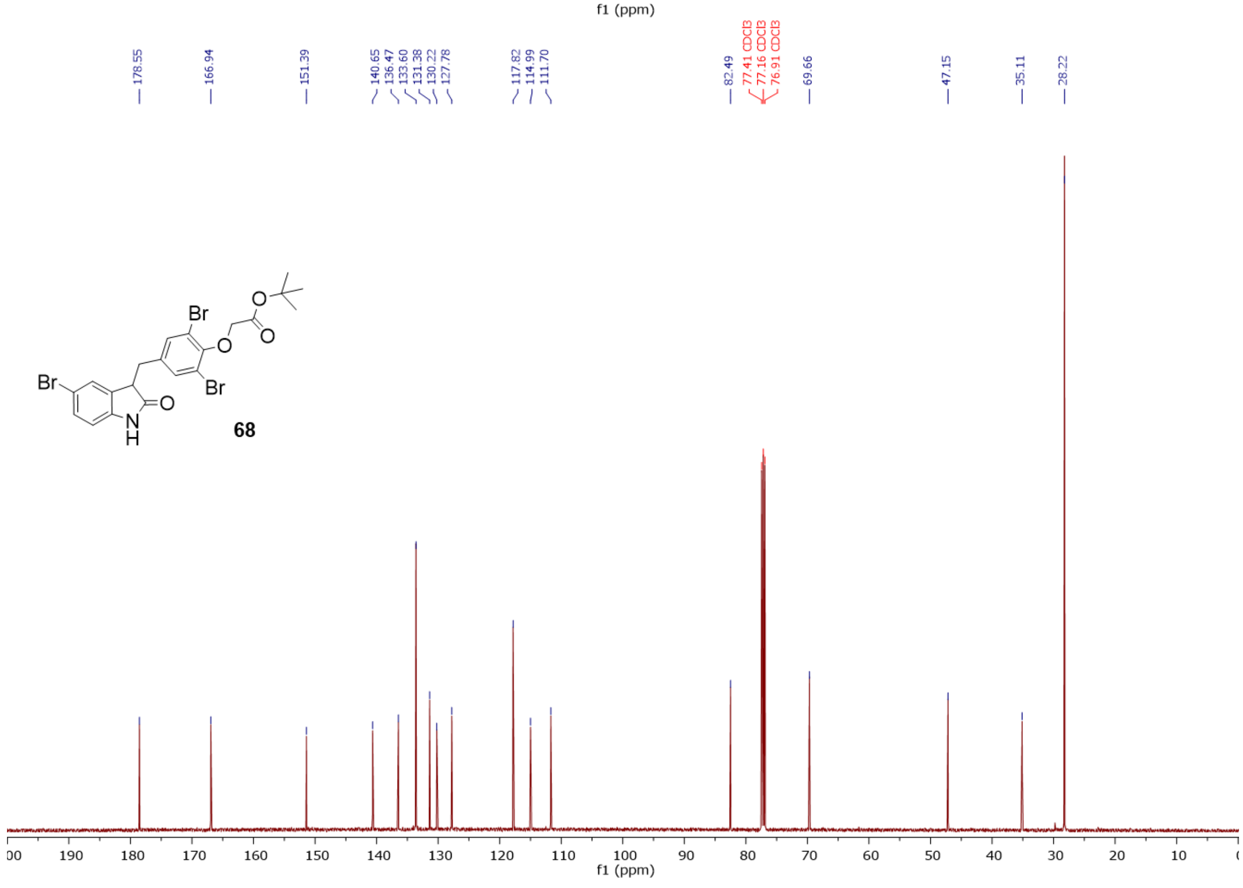
178.55
166.94
151.39
140.65
136.47
133.60
131.38
130.22
127.78
117.82
114.99
111.70
82.49
77.41 CDCl3
77.16 CDCl3
76.91 CDCl3
69.66
47.15
35.11
28.22
Br
O
O
O
Br
Br
N
H
O
68
f1 (ppm)

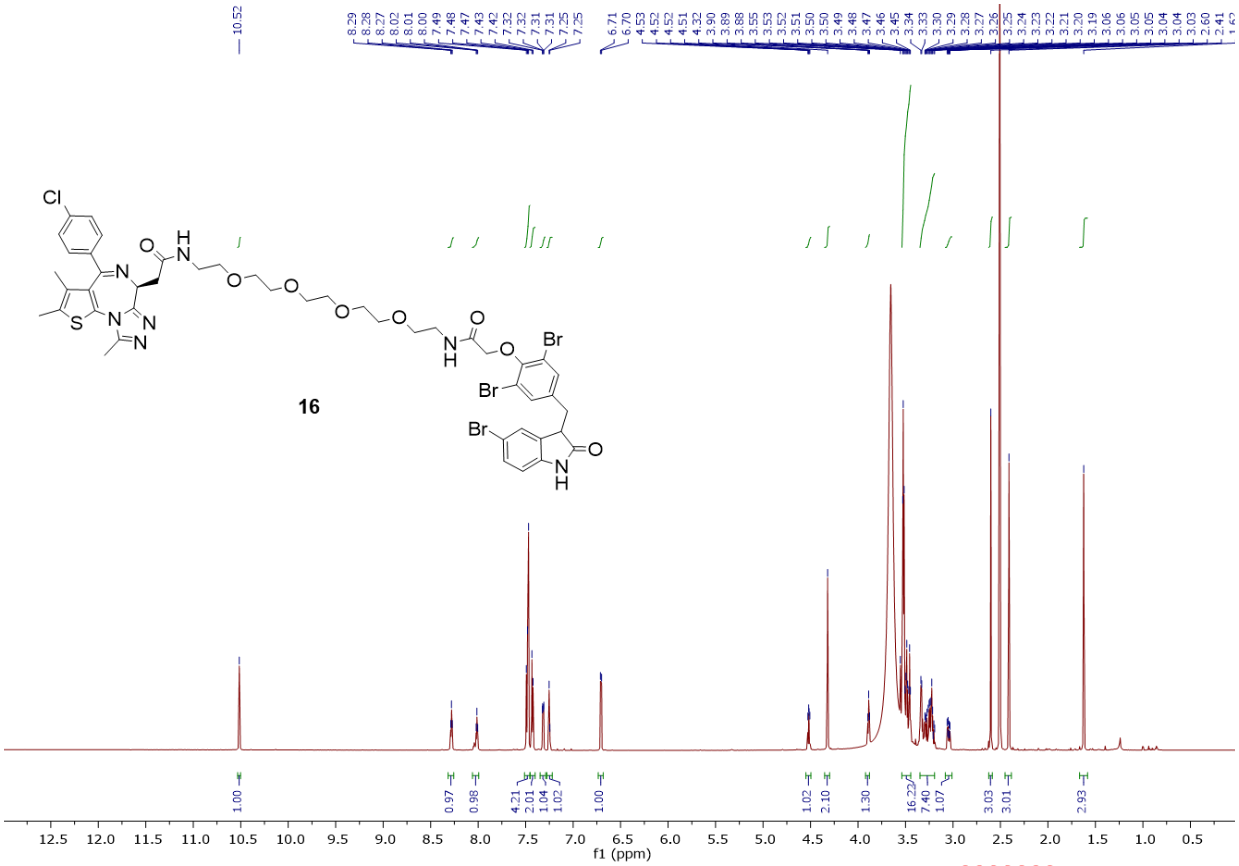
10.52
8.29 8.28 8.27 8.02 8.01 8.00 7.49 7.48 7.47 7.43 7.42 7.32 7.32 7.31 7.31 7.25 7.25
6.71 6.70 4.53 4.52 4.52 4.51 4.32 3.90 3.89 3.88 3.55 3.53 3.52 3.51 3.50 3.50 3.49 3.48 3.47 3.46 3.45 3.34 3.33 3.30 3.29 3.28 3.27 3.26 3.25 3.24 3.23 3.22 3.21 3.20 3.19 3.06 3.05 3.05 3.04 3.04 3.03 2.60 2.41 1.62
Cl
H
N
O
N
S
N
N
O
O
O
O
N
H
O
O
Br
Br
Br
N
H
O
16
1.00
0.97
0.98
4.21
2.01
1.04
1.02
1.00
1.02
2.10
1.30
16.22
7.40
1.07
3.03
3.01
2.93
12.5 12.0 11.5 11.0 10.5 10.0 9.5 9.0 8.5 8.0 7.5 7.0 6.5 6.0 5.5 5.0 4.5 4.0 3.5 3.0 2.5 2.0 1.5 1.0 0.5
f1 (ppm)

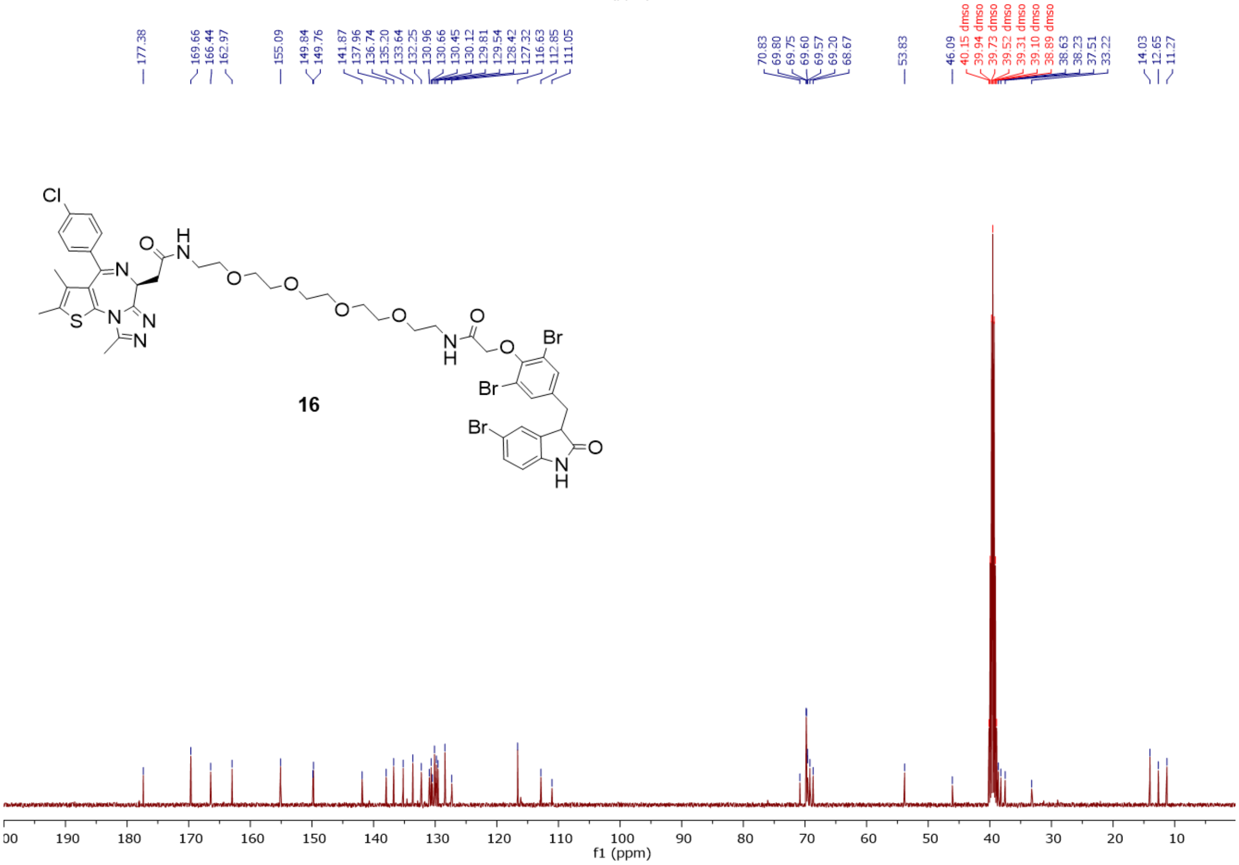
177.38
169.66 166.44 162.97
155.09
149.84 149.76
141.87 137.96 136.74 135.20 133.64 132.25 130.96 130.66 130.45 130.12 129.81 129.54 128.42 127.32 116.63 112.85 111.05
70.83 69.80 69.75 69.60 69.57 69.20 68.67
53.83
46.09
40.15 dmso 39.94 dmso 39.73 dmso 39.52 dmso 39.31 dmso 39.10 dmso 38.89 dmso
38.63 38.23 37.51 33.22
14.03 12.65 11.27
16
00 190 180 170 160 150 140 130 120 110 100 90 80 70 60 50 40 30 20 10
f1 (ppm)

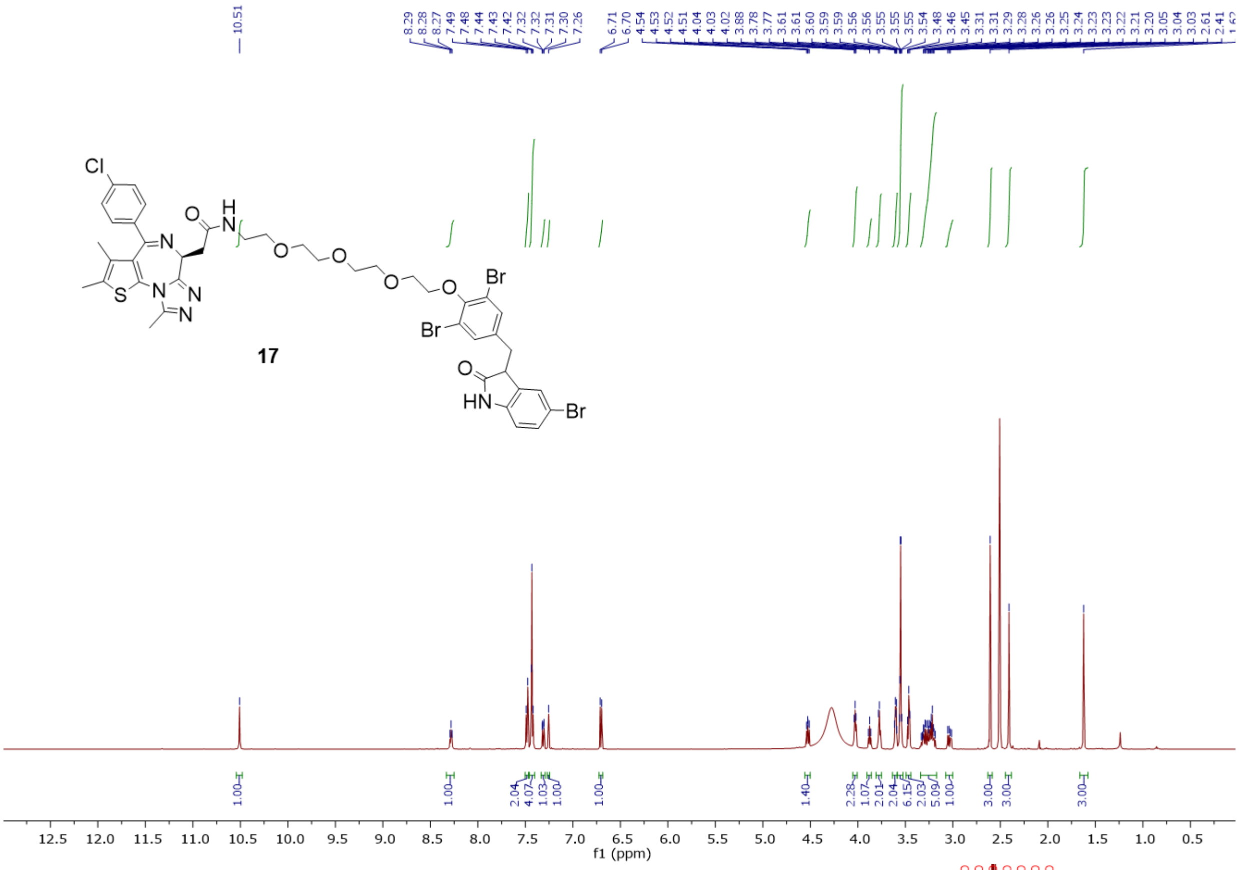
10.51
8.29 8.28 8.27 7.49 7.48 7.44 7.43 7.42 7.32 7.32 7.31 7.30 7.26
6.71 6.70 4.54 4.53 4.52 4.51 4.04 4.03 4.02 3.88 3.78 3.77 3.61 3.61 3.60 3.59 3.59 3.56 3.56 3.55 3.55 3.55 3.54 3.48 3.46 3.45 3.31 3.31 3.29 3.28 3.26 3.26 3.25 3.24 3.23 3.23 3.22 3.21 3.20 3.05 3.04 3.03 2.61 2.41
Cl
O
H
N
N
S
N
N
N
O
O
O
O
Br
Br
17
O
HN
Br
1.00 1.00 2.04 4.07 1.03 1.00 1.00 1.40 2.28 1.07 2.01 2.04 6.15 2.03 5.09 1.00 3.00 3.00 3.00
12.5 12.0 11.5 11.0 10.5 10.0 9.5 9.0 8.5 8.0 7.5 7.0 6.5 6.0 5.5 5.0 4.5 4.0 3.5 3.0 2.5 2.0 1.5 1.0 0.5
f1 (ppm)

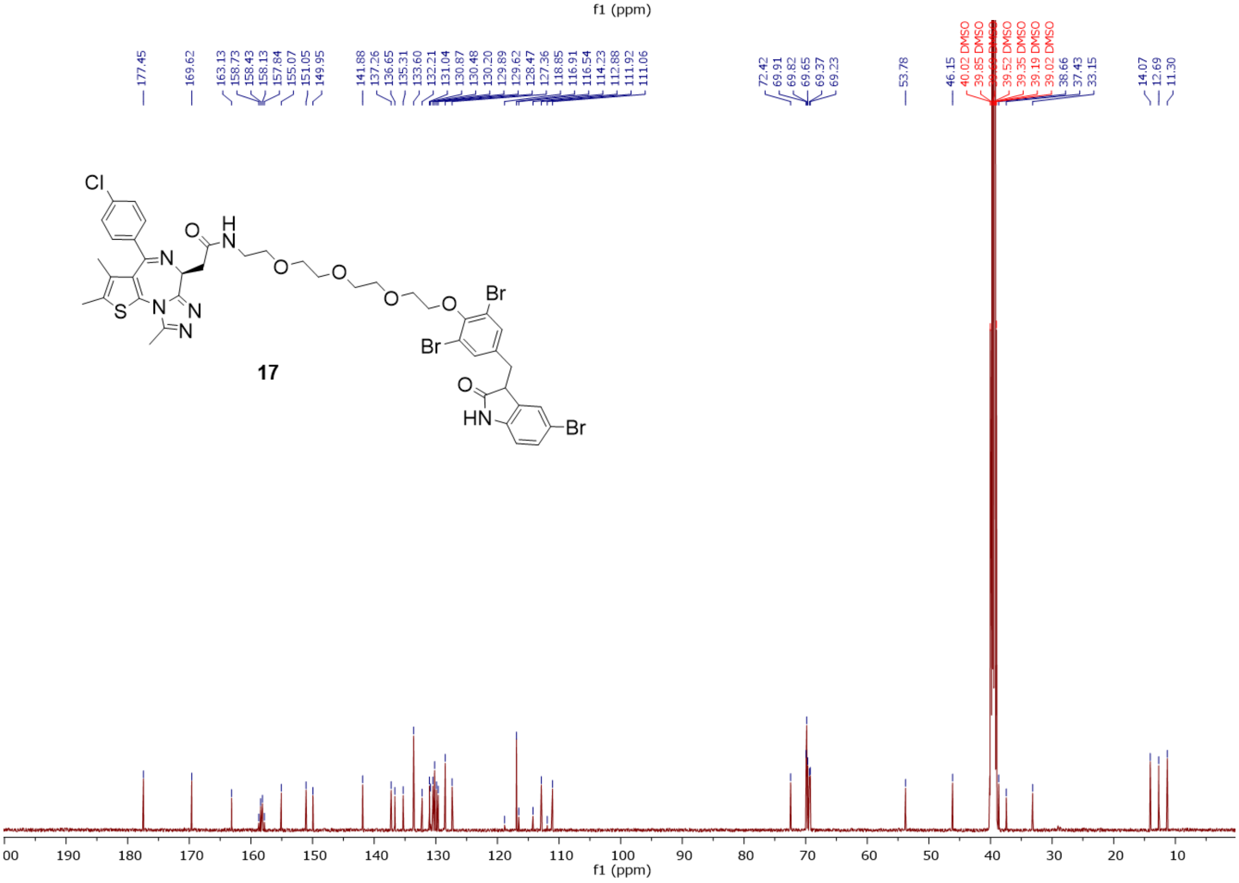
177.45
169.62
163.13 158.73 158.43 158.13 157.84 155.07 151.05 149.95
141.88 137.26 136.65 135.31 133.60 132.21 131.04 130.87 130.48 130.20 129.89 129.62 128.47 127.36 118.85 116.91 116.54 114.23 112.88 111.92 111.06
72.42 69.91 69.82 69.65 69.37 69.23
53.78
46.15
40.02 DMSO 39.85 DMSO 39.52 DMSO 39.35 DMSO 39.19 DMSO 39.02 DMSO
38.66 37.43 33.15
14.07 12.69 11.30
Cl
O
H
N
N
S
N
N
N
O
O
O
O
Br
Br
17
O
HN
Br
00 190 180 170 160 150 140 130 120 110 100 90 80 70 60 50 40 30 20 10
f1 (ppm)

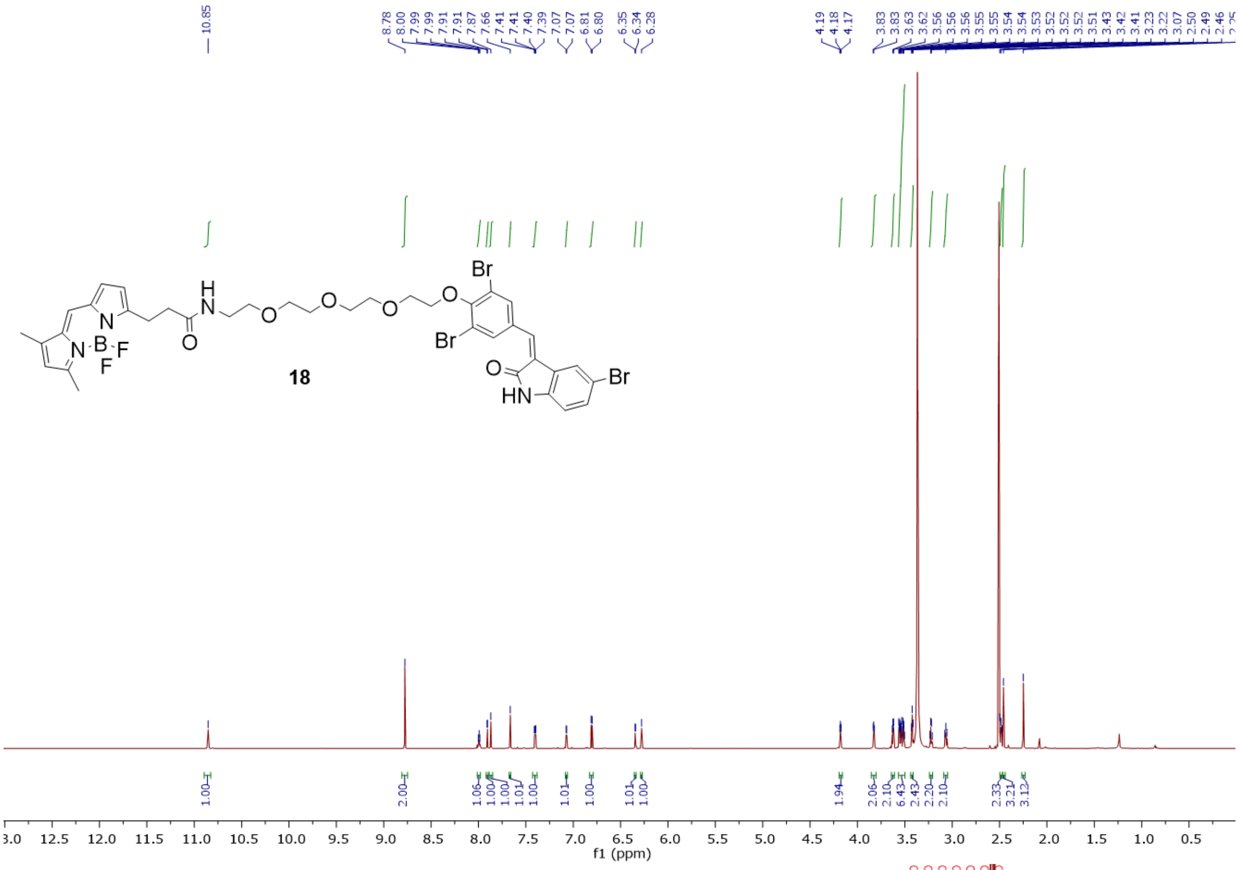
10.85
8.78 8.00 7.99 7.99 7.91 7.91 7.87 7.66 7.41 7.41 7.40 7.39 7.07 7.07 6.81 6.80 6.35 6.34 6.28
4.19 4.18 4.17 3.83 3.83 3.63 3.62 3.56 3.56 3.56 3.55 3.55 3.54 3.54 3.53 3.52 3.52 3.52 3.51 3.43 3.42 3.41 3.23 3.22 3.07 2.50 2.49 2.46
Br
O
H
N
O
O
O
N
N
B
F
F
O
Br
O
HN
Br
18
1.00 2.00 1.06 1.00 1.00 1.01 1.00 1.01 1.00 1.01 1.00 1.94 2.06 2.10 6.43 2.43 2.20 2.10 2.33 3.21 3.12
f1 (ppm)

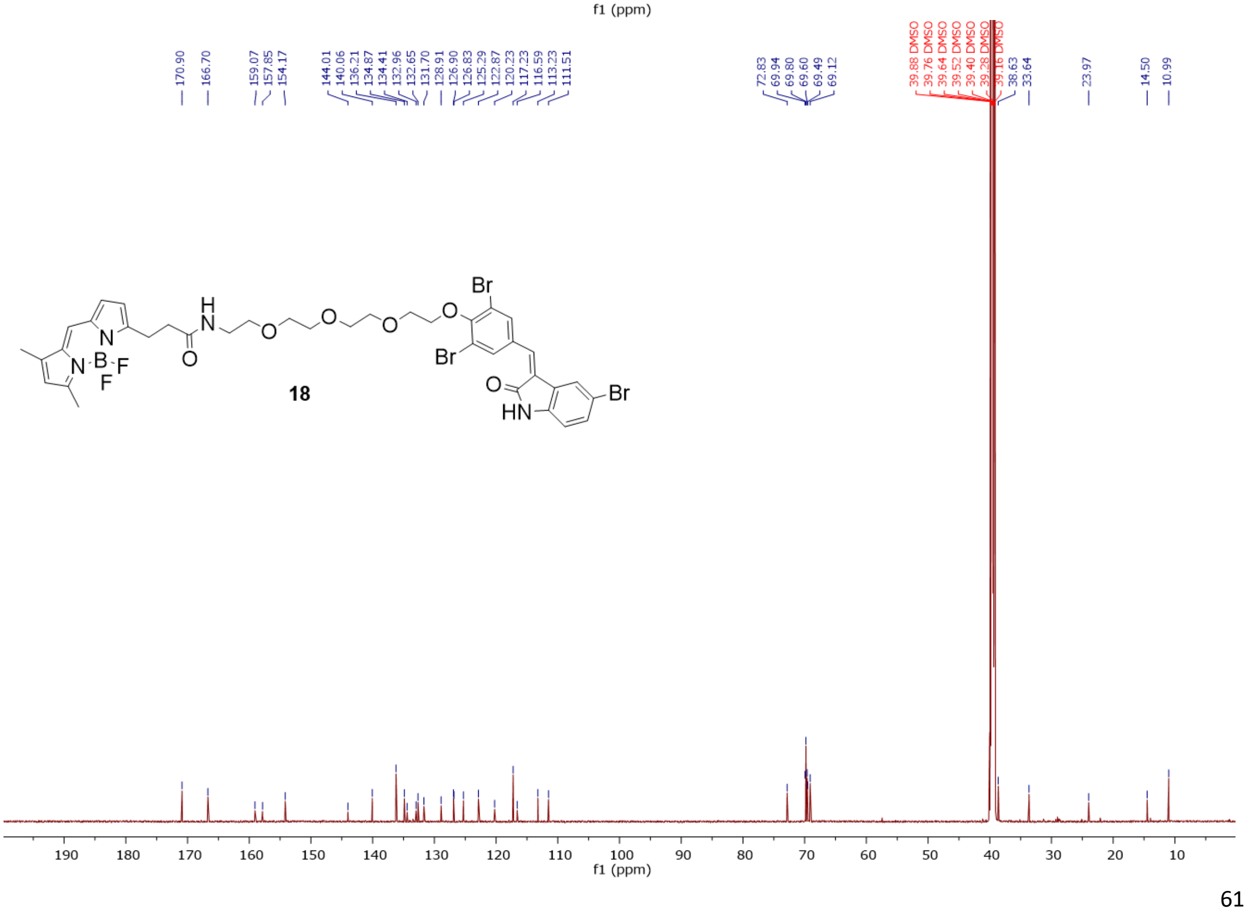
170.90 166.70 159.07 157.85 154.17 144.01 140.06 136.21 134.87 134.41 132.96 132.65 131.70 128.91 126.90 126.83 125.29 122.87 120.23 117.23 116.59 113.23 111.51
72.83 69.94 69.80 69.60 69.49 69.12
39.88 DMSO 39.76 DMSO 39.64 DMSO 39.52 DMSO 39.40 DMSO 39.28 DMSO 39.16 DMSO 38.63 33.64 23.97 14.50 10.99
18
f1 (ppm)

References

1. Cheng, J. et al. Discovery of novel PDEδ degraders for the treatment of KRAS mutant colorectal cancer. *J. Med. Chem.* **63**, 7892-7905 (2020).

2. Winzker, M. et al. Development of a PDEδ-targeting PROTACs that impair lipid metabolism. *Angew. Chem. Int. Ed.* **59**, 5595-5601 (2020).

3. Pei, J. *et al.* Developing potent LC3-targeting AUTAC tools for protein degradation with selective autophagy. *Chem. Commun.* **57**, 13194-13197 (2021).

4. In-Cell Western Assay. LI-COR Biosciences, https://www.licor.com/bio/applications/in-cell-western-assay (11.01.2023).

5. Murarka, S. et al. Development of pyridazinone chemotypes targeting the PDEδ prenyl binding site. *Chem. Eur. J.* **23**, 6083-6093 (2017).

6. Hirst, Gavin C. et al. Preparation of 3-(azahetero)aryl-1H-pyrazolo[3,4-d] pyrimidin-3-amines as protein kinase inhibitors with antiangiogenic properties. PCT Int. Appl., 2002080926, 17 Oct 2002.
