# Supplementary material for: Discovery of a Drug-like, Natural Product-Inspired DCAF11 Ligand Chemotype
Source: Nat Commun. 2023 Nov 30;14:7908. doi: 10.1038/s41467-023-43657-6 (PMC10689823; doi:10.1038/s41467-023-43657-6)
Supplement: Supplementary file 5 — Reporting Summary [file 41467_2023_43657_MOESM5_ESM.pdf]

nature portfolio

Corresponding author(s): Georg Winter and Herbert Waldmann

Last updated by author(s): Oct 28, 2023

Reporting Summary

Nature Portfolio wishes to improve the reproducibility of the work that we publish. This form provides structure for consistency and transparency in reporting. For further information on Nature Portfolio policies, see our Editorial Policies and the Editorial Policy Checklist.

Statistics

For all statistical analyses, confirm that the following items are present in the figure legend, table legend, main text, or Methods section.

n/a	Confirmed	
☐	☒	The exact sample size (*n*) for each experimental group/condition, given as a discrete number and unit of measurement
☐	☒	A statement on whether measurements were taken from distinct samples or whether the same sample was measured repeatedly
☐	☒	The statistical test(s) used AND whether they are one- or two-sided<br>*Only common tests should be described solely by name; describe more complex techniques in the Methods section.*
☒	☐	A description of all covariates tested
☐	☒	A description of any assumptions or corrections, such as tests of normality and adjustment for multiple comparisons
☐	☒	A full description of the statistical parameters including central tendency (e.g. means) or other basic estimates (e.g. regression coefficient) AND variation (e.g. standard deviation) or associated estimates of uncertainty (e.g. confidence intervals)
☐	☒	For null hypothesis testing, the test statistic (e.g. *F*, *t*, *r*) with confidence intervals, effect sizes, degrees of freedom and *P* value noted<br>*Give P values as exact values whenever suitable.*
☒	☐	For Bayesian analysis, information on the choice of priors and Markov chain Monte Carlo settings
☒	☐	For hierarchical and complex designs, identification of the appropriate level for tests and full reporting of outcomes
☒	☐	Estimates of effect sizes (e.g. Cohen's *d*, Pearson's *r*), indicating how they were calculated

Our web collection on statistics for biologists contains articles on many of the points above.

Software and code

Policy information about availability of computer code

Data collection	Western blots: Bio-Rad ChemiDoc MP Imaging System. in-cell Western: Licor Odyssey CLx. Cell viability and Caspase activity: Tecan Spark® Multimode Microplate Reader. qPCR: CFX96 Touch™ Real-Time PCR Detection System and Bio-Rad CFX Manager v3.1. DSF experiment: Lightcycler L480.<br>Flow cytometry: Data was collected on a BD LSRFortessa using BD FACSDiva software (v9.0), BD FACSAria Fusion using BD FACSDiva software (v8.0.2) or Beckman Coulter CytoFLEX SRT using CytExpert SRT (v 1.1.0.10007)<br>NGS: Illumina HiSeq3500 (https://www.illumina.com/)
Data analysis	Western blots: ImageLab (v6.0.1). in-cell Western: Image Studio (v5.2). qPCR, Cell viability and Caspase activity: Graphpad Prism (v9.2.0). DSF: Graphpad Prism (v9.5.1)<br>Flow Cytometry Analysis: Flowjo (v10.8.1)<br>FACS-based CRISPR screens: All relevant software is described in detail in the corresponding methods section. Pipelines for sgRNA quantification and statistical analysis are available on Github (https://github.com/ZuberLab/crispr-processnf/tree/566f6d46bbcc2a3f49f51bbc96b9820f408ec4a3 and https://github.com/ZuberLab/crisprmageck-nf/tree/c75a90f670698bfa78bfd8be786d6e5d6d4fc455). Used packages: fastx-toolkit (v0.0.14), bowtie2 (v2.4.5), featureCounts (v2.0.1), MAGeCK (v0.5.9).<br>Data compiling, processing and statistical analyses: Microsoft Excel for Microsoft 365 (v2208.16.0.15601.20526), R Studio (v2022.12.0 Build 353) with R (v4.2.2)

For manuscripts utilizing custom algorithms or software that are central to the research but not yet described in published literature, software must be made available to editors and reviewers. We strongly encourage code deposition in a community repository (e.g. GitHub). See the Nature Portfolio guidelines for submitting code & software for further information.

Data

Policy information about availability of data

All manuscripts must include a data availability statement. This statement should provide the following information, where applicable:

- Accession codes, unique identifiers, or web links for publicly available datasets
- A description of any restrictions on data availability
- For clinical datasets or third party data, please ensure that the statement adheres to our policy

Data in the FACS-based CRISPR screen are presented in supplementary Table 1 and Table 2. The inhibition activities of 42 against kinases generated in this study are presented in supplementary Table 4. The remaining data are available within the paper, Supplementary Information or Source Data File. Source data are provided with this paper.

Research involving human participants, their data, or biological material

Policy information about studies with human participants or human data. See also policy information about sex, gender (identity/presentation), and sexual orientation and race, ethnicity and racism.

Reporting on sex and gender	N/A
Reporting on race, ethnicity, or other socially relevant groupings	N/A
Population characteristics	N/A
Recruitment	N/A
Ethics oversight	N/A

Note that full information on the approval of the study protocol must also be provided in the manuscript.

Field-specific reporting

Please select the one below that is the best fit for your research. If you are not sure, read the appropriate sections before making your selection.

☒ Life sciences ☐ Behavioural & social sciences ☐ Ecological, evolutionary & environmental sciences

For a reference copy of the document with all sections, see nature.com/documents/nr-reporting-summary-flat.pdf

Life sciences study design

All studies must disclose on these points even when the disclosure is negative.

Sample size	For quantitative experiments, a sample size of at least 2, typically 3 to 5 independent experiment was chosen in line with what is the standard of the field in the molecular biosciences. For non-quantitative experiments, a sample size of at least 2, typically 3 to 5 independent experiment was chosen in line with what is the standard of the field in the molecular biosciences.
Data exclusions	No data was excluded.
Replication	All observations were made in at least two independent experiments, typically with technical replicates, all with consistent results. The numbers of independent experiments is given in the "Statistics and reproducibility" section and individual results are shown were possible.
Randomization	Randomization was not applicable as no experiments involving human/animals were performed, and no included experiment were sensitive to the order of measurement/treatment.
Blinding	Blinding was not carried out as no subjective analysis was performed.

Behavioural & social sciences study design

All studies must disclose on these points even when the disclosure is negative.

Study description	*Briefly describe the study type including whether data are quantitative, qualitative, or mixed-methods (e.g. qualitative cross-sectional, quantitative experimental, mixed-methods case study).*
Research sample	*State the research sample (e.g. Harvard university undergraduates, villagers in rural India) and provide relevant demographic information (e.g. age, sex) and indicate whether the sample is representative. Provide a rationale for the study sample chosen. For*

	studies involving existing datasets, please describe the dataset and source.
Sampling strategy	*Describe the sampling procedure (e.g. random, snowball, stratified, convenience). Describe the statistical methods that were used to predetermine sample size OR if no sample-size calculation was performed, describe how sample sizes were chosen and provide a rationale for why these sample sizes are sufficient. For qualitative data, please indicate whether data saturation was considered, and what criteria were used to decide that no further sampling was needed.*
Data collection	*Provide details about the data collection procedure, including the instruments or devices used to record the data (e.g. pen and paper, computer, eye tracker, video or audio equipment) whether anyone was present besides the participant(s) and the researcher, and whether the researcher was blind to experimental condition and/or the study hypothesis during data collection.*
Timing	*Indicate the start and stop dates of data collection. If there is a gap between collection periods, state the dates for each sample cohort.*
Data exclusions	*If no data were excluded from the analyses, state so OR if data were excluded, provide the exact number of exclusions and the rationale behind them, indicating whether exclusion criteria were pre-established.*
Non-participation	*State how many participants dropped out/declined participation and the reason(s) given OR provide response rate OR state that no participants dropped out/declined participation.*
Randomization	*If participants were not allocated into experimental groups, state so OR describe how participants were allocated to groups, and if allocation was not random, describe how covariates were controlled.*

Ecological, evolutionary & environmental sciences study design

All studies must disclose on these points even when the disclosure is negative.

Study description	*Briefly describe the study. For quantitative data include treatment factors and interactions, design structure (e.g. factorial, nested, hierarchical), nature and number of experimental units and replicates.*
Research sample	*Describe the research sample (e.g. a group of tagged Passer domesticus, all Stenocereus thurberi within Organ Pipe Cactus National Monument), and provide a rationale for the sample choice. When relevant, describe the organism taxa, source, sex, age range and any manipulations. State what population the sample is meant to represent when applicable. For studies involving existing datasets, describe the data and its source.*
Sampling strategy	*Note the sampling procedure. Describe the statistical methods that were used to predetermine sample size OR if no sample-size calculation was performed, describe how sample sizes were chosen and provide a rationale for why these sample sizes are sufficient.*
Data collection	*Describe the data collection procedure, including who recorded the data and how.*
Timing and spatial scale	*Indicate the start and stop dates of data collection, noting the frequency and periodicity of sampling and providing a rationale for these choices. If there is a gap between collection periods, state the dates for each sample cohort. Specify the spatial scale from which the data are taken*
Data exclusions	*If no data were excluded from the analyses, state so OR if data were excluded, describe the exclusions and the rationale behind them, indicating whether exclusion criteria were pre-established.*
Reproducibility	*Describe the measures taken to verify the reproducibility of experimental findings. For each experiment, note whether any attempts to repeat the experiment failed OR state that all attempts to repeat the experiment were successful.*
Randomization	*Describe how samples/organisms/participants were allocated into groups. If allocation was not random, describe how covariates were controlled. If this is not relevant to your study, explain why.*
Blinding	*Describe the extent of blinding used during data acquisition and analysis. If blinding was not possible, describe why OR explain why blinding was not relevant to your study.*

Did the study involve field work? ☐ Yes ☐ No

Field work, collection and transport

Field conditions	*Describe the study conditions for field work, providing relevant parameters (e.g. temperature, rainfall).*
Location	*State the location of the sampling or experiment, providing relevant parameters (e.g. latitude and longitude, elevation, water depth).*
Access & import/export	*Describe the efforts you have made to access habitats and to collect and import/export your samples in a responsible manner and in compliance with local, national and international laws, noting any permits that were obtained (give the name of the issuing authority, the date of issue, and any identifying information).*

Disturbance | Describe any disturbance caused by the study and how it was minimized.

Reporting for specific materials, systems and methods

We require information from authors about some types of materials, experimental systems and methods used in many studies. Here, indicate whether each material, system or method listed is relevant to your study. If you are not sure if a list item applies to your research, read the appropriate section before selecting a response.

Materials & experimental systems

n/a	Involved in the study
☐	☒ Antibodies
☐	☒ Eukaryotic cell lines
☒	☐ Palaeontology and archaeology
☒	☐ Animals and other organisms
☒	☐ Clinical data
☒	☐ Dual use research of concern
☒	☐ Plants

Methods

n/a	Involved in the study
☒	☐ ChIP-seq
☐	☒ Flow cytometry
☒	☐ MRI-based neuroimaging

Antibodies

Antibodies used

The following primary antibodies were used for immunoblot analyses:
BRD2 (5848S; Cell Signaling, RRID:AB_10835146, 1:1000), BRD4 (13440S, Cell Signaling, RRID:AB_2687578, 1:1000), LC3B (2775S, Cell Signaling, RRID:AB_915950, 1:1000), GAPDH (2118S, Cell Signaling, RRID:AB_561053, 1:1000), DDB1 (6998S, Cell Signaling, RRID:AB_10829458, 1:1000), BTK (8547S, Cell Signaling, RRID:AB_10950506, 1:1000) and BLK (3262S, Cell Signaling, RRID:AB_2063358, 1:1000), BRD3 (ab50818, Abcam, RRID:AB_868478. 1:200), RBX1 (ab133565, Abcam, 1:1000), PDEdelta (PA5-22008, Invitrogen, RRID:AB_11154288,, 1:1000), Vinculin (V9131, Sigma, RRID:AB_477629, 1:1000), DCAF11 (A15519, ABclonal, RRID:AB_2762920, 1:1000). HA tag antibody: (thermo fisher, RRID:AB_10978021, 26183)

The following primary antibodies were used for In-Cell Western:
BRD2 (ab139690, Abcam, RRID:AB_2737409; 1:800)

The following secondary antibodies were used for immunoblot analyses:
goat anti-rabbit (31460 , Invitrogen, RRID:AB_228341, 1:500), anti-mouse HRP (62-6520, Invitrogen, RRID:AB_88369; 1:500)

The following antibodies were used for flow cytometry: APC anti-CD90.1/Thy1.1 (202526, BioLegend, RRID:AB_1595470, 1:500), human Fc Receptor Blocking Solution (422302, BioLegend, RRID:AB_2818986, 1:100 – 1:1000)

Validation

Antibodies were validated for the application of Western Blotting as per statements on their manufacturers websites. Antibodies for DCAF11, DDB1 and LC3B were further validated via knockout cells, and antibody for RBX1 were validated via siRNA.

Validation statements of primary antibodies as per manufactures' websites:
5848S targets BRD2 in WB, ChIP and C&R application and shows reactivity with human and mouse samples.
13440S targets BRD4 in WB, IP, IHC, ChIP, eCLIP and C&R application and shows reactivity with human samples.
2775S targets LC3B in WB application and shows reactivity with human, mouse and rat samples.
2118S targets GAPDH in WB, IHC, IF and F application and shows reactivity with human, mouse, monkey, bovine, pig and rat samples.
6998S targets DDB1 in WB application and shows reactivity with human, mouse, monkey and rat samples.
8547S targets BTK in WB, IP, IHC and F application and shows reactivity with human and mouse samples.
3262S targets BLK in WB application and shows reactivity with human samples.
ab50818 targets BRD3 in Flow Cyt, WB, IP and ICC/IF application and shows reactivity with human, mouse and rat samples.
ab133565 targets RBX1 in Flow Cyt, IP, IHC-P, WB and ICC/IF application and shows reactivity with human, mouse and rat samples.
PA5-22008 targets PDEdelta in IHC-P, WB and ICC/IF application and shows reactivity with human and mouse samples.
V9131 targets Vinculin in WB, IHC, ICC/IF and IP application and shows reactivity with human and mouse samples.
A15519 targets DCAF11 in WB application and shows reactivity with human, rat and mouse samples.
26183 targets HA, and was verified by Relative expression to ensure that the antibody binds to the antigen stated.
ab139690 targets BRD2 in WB, ICC/IF, IHC-P and Flow Cyt application and shows reactivity with human samples.

Eukaryotic cell lines

Policy information about cell lines and Sex and Gender in Research

Cell line source(s)

HAP1 (male, C631, Horizon, RRID:CVCL_Y019), Jurkat (male, ACC-282, DSMZ, RID:CVCL_0065), Ramos (male, ACC-603, DSMZ, RRID:CVCL_0597), HEK293T (female, CRL-11268, ATCC, RRID:CVCL_1926), MDA-MB-231 (female, ACC 732, DSMZ, RRID:CVCL_0062) and Hep G2 (male, ACC 180, DSMZ, CVCL_0027)
KBM7 cells (male, from T. Brummelkamp lab (Carette et al, Science, 2009); KBM7 cells constitutively expressing Cas9 (from Mayor-Ruiz et al, Nat Chem Biol, 2020). KBM7 iCas9 cells were a gift from Johannes Zuber (IMP - Research Institute of

	Molecular Pathology).
Authentication	Used KBM cell lines were authenticated by short tandem repeat profiling. Other cells were used without authentication.
Mycoplasma contamination	All used cell lines were routinely tested and confirmed negative for mycoplasma contamination.
Commonly misidentified lines (See ICLAC register)	No commonly misidentified cell lines were used.

Palaeontology and Archaeology

Specimen provenance	*Provide provenance information for specimens and describe permits that were obtained for the work (including the name of the issuing authority, the date of issue, and any identifying information). Permits should encompass collection and, where applicable, export.*
Specimen deposition	*Indicate where the specimens have been deposited to permit free access by other researchers.*
Dating methods	*If new dates are provided, describe how they were obtained (e.g. collection, storage, sample pretreatment and measurement), where they were obtained (i.e. lab name), the calibration program and the protocol for quality assurance OR state that no new dates are provided.*

☐ Tick this box to confirm that the raw and calibrated dates are available in the paper or in Supplementary Information.

Ethics oversight	*Identify the organization(s) that approved or provided guidance on the study protocol, OR state that no ethical approval or guidance was required and explain why not.*

Note that full information on the approval of the study protocol must also be provided in the manuscript.

Animals and other research organisms

Policy information about studies involving animals; ARRIVE guidelines recommended for reporting animal research, and Sex and Gender in Research

Laboratory animals	*For laboratory animals, report species, strain and age OR state that the study did not involve laboratory animals.*
Wild animals	*Provide details on animals observed in or captured in the field; report species and age where possible. Describe how animals were caught and transported and what happened to captive animals after the study (if killed, explain why and describe method; if released, say where and when) OR state that the study did not involve wild animals.*
Reporting on sex	*Indicate if findings apply to only one sex; describe whether sex was considered in study design, methods used for assigning sex. Provide data disaggregated for sex where this information has been collected in the source data as appropriate; provide overall numbers in this Reporting Summary. Please state if this information has not been collected. Report sex-based analyses where performed, justify reasons for lack of sex-based analysis.*
Field-collected samples	*For laboratory work with field-collected samples, describe all relevant parameters such as housing, maintenance, temperature, photoperiod and end-of-experiment protocol OR state that the study did not involve samples collected from the field.*
Ethics oversight	*Identify the organization(s) that approved or provided guidance on the study protocol, OR state that no ethical approval or guidance was required and explain why not.*

Note that full information on the approval of the study protocol must also be provided in the manuscript.

Clinical data

Policy information about clinical studies

All manuscripts should comply with the ICMJEguidelines for publication of clinical research and a completedCONSORT checklist must be included with all submissions.

Clinical trial registration	*Provide the trial registration number from ClinicalTrials.gov or an equivalent agency.*
Study protocol	*Note where the full trial protocol can be accessed OR if not available, explain why.*
Data collection	*Describe the settings and locales of data collection, noting the time periods of recruitment and data collection.*
Outcomes	*Describe how you pre-defined primary and secondary outcome measures and how you assessed these measures.*

Dual use research of concern

Policy information about dual use research of concern

Hazards

Could the accidental, deliberate or reckless misuse of agents or technologies generated in the work, or the application of information presented in the manuscript, pose a threat to:

No	Yes	
☐	☐	Public health
☐	☐	National security
☐	☐	Crops and/or livestock
☐	☐	Ecosystems
☐	☐	Any other significant area

Experiments of concern

Does the work involve any of these experiments of concern:

No	Yes	
☐	☐	Demonstrate how to render a vaccine ineffective
☐	☐	Confer resistance to therapeutically useful antibiotics or antiviral agents
☐	☐	Enhance the virulence of a pathogen or render a nonpathogen virulent
☐	☐	Increase transmissibility of a pathogen
☐	☐	Alter the host range of a pathogen
☐	☐	Enable evasion of diagnostic/detection modalities
☐	☐	Enable the weaponization of a biological agent or toxin
☐	☐	Any other potentially harmful combination of experiments and agents

Plants

Seed stocks	N/A
Novel plant genotypes	N/A
Authentication	N/A

ChIP-seq

Data deposition

☐ Confirm that both raw and final processed data have been deposited in a public database such as GEO.

☐ Confirm that you have deposited or provided access to graph files (e.g. BED files) for the called peaks.

Data access links<br>*May remain private before publication.*	*For "Initial submission" or "Revised version" documents, provide reviewer access links. For your "Final submission" document, provide a link to the deposited data.*
Files in database submission	*Provide a list of all files available in the database submission.*
Genome browser session<br>(e.g. UCSC)	*Provide a link to an anonymized genome browser session for "Initial submission" and "Revised version" documents only, to enable peer review. Write "no longer applicable" for "Final submission" documents.*

Methodology

Replicates	*Describe the experimental replicates, specifying number, type and replicate agreement.*
Sequencing depth	*Describe the sequencing depth for each experiment, providing the total number of reads, uniquely mapped reads, length of reads and whether they were paired- or single-end.*
Antibodies	*Describe the antibodies used for the ChIP-seq experiments; as applicable, provide supplier name, catalog number, clone name, and lot number.*
Peak calling parameters	*Specify the command line program and parameters used for read mapping and peak calling, including the ChIP, control and index files used.*
Data quality	*Describe the methods used to ensure data quality in full detail, including how many peaks are at FDR 5% and above 5-fold enrichment.*
Software	*Describe the software used to collect and analyze the ChIP-seq data. For custom code that has been deposited into a community repository, provide accession details.*

Flow Cytometry

Plots

Confirm that:

- [x] The axis labels state the marker and fluorochrome used (e.g. CD4-FITC).
- [x] The axis scales are clearly visible. Include numbers along axes only for bottom left plot of group (a 'group' is an analysis of identical markers).
- [x] All plots are contour plots with outliers or pseudocolor plots.
- [x] A numerical value for number of cells or percentage (with statistics) is provided.

Methodology

Sample preparation	Sample preparation for FACS-based BRD4 stability screens is described in detail in the methods section.<br>To engineer a BRD4 protein stability reporer, KBM7 iCas9 cells were transduced with lentivirus expressing SFFV-BRD4(S)-mTagBFP-P2A-mCherry and BFP/mCherry double positive cells were sorted on a Cytoflex SRT (Beckman Coulter) to generate a stable reporter cell line. For evaluation of BRD4-TagBFP reporter degradation, cells were treated with DMSO or BRD4 degraders for 6 hours before flow cytometry analysis on an LSRFortessa (BD Biosciences).<br>For BRD4 stability CRISPR screens, library transduced cells were induced with doxycycline for 3 days and harvested after 6 hours of treatment with DMSO or degraders. Cells were washed with PBS, stained with Zombie NIR™ Fixable Viability Dye (1:1000, BioLegend) and APC anti-mouse CD90.1/Thy-1.1 antibody (1:400, BioLegend) in the presence of Human TruStain FcX™ Fc Receptor Blocking Solution (1:400, BioLegend), and fixed with 0.5 mL methanol-free paraformaldehyde 4% (Thermo Scientific™ Pierce™) for 30 min at 4 ºC, while protected from light. Cells were washed with, and stored in FACS buffer (PBS containing 5% FBS and 1 mM EDTA) at 4 ºC over night. The next day, cells were strained trough a 35 µm nylon mesh and sorted on a BD FACSAria™ Fusion (BD Biosciences) using a 85 µm nozzle. Aggregates, dead (ZombieNIR positive), Cas9-negative (GFP) and sgRNA library-negative (Thy1.1-APC) cells were excluded, and the remaining cells were sorted based on their BRD4-BFP and mCherry levels into BRD4HIGH (~10% of cells), BRD4MID (~30%) and BRD4LOW (~10%) fractions. For each sample, cells corresponding to at least 1,500-fold library representation were sorted per replicate.
Instrument	All flow cytometric analyses were performed on BD LSRFortessa (4 laser, 16 detector configuration; BD Bioscience). All sorts were performed on BD FACSAria Fusion (5 lasers, 16 detectors; BD Bioscience) or CytoFLEX SRT (4 lasers, 15 detectors; Beckman Coulter) cell sorters.
Software	BD FACSDiva software (v8.0.2 and v9.0), Beckman Coulter CytExpert SRT (v 1.1.0.10007), Flowjo (v10.8.1)
Cell population abundance	In BRD4 stability CRISPR screens, cells were sorted into BRD4HIGH (~10% of cells), BRD4LOW (~10%),<br>and BRD4MID (~30%) populations. All collected fractions were reanalyzed for purity on an LSRFortessa before further processing.
Gating strategy	In all analyses, forward scatter area vs. side scatter area plot was used to separate cell events from debris and dead cells.<br>Forward scatter height vs. forward scatter area and/or side scatter width vs. side scatter height plots were used to separate single cells from aggregates.<br>In BRD4 protein stability screens, Zombie-NIR staining (BV786-A) vs. forward scatter area was used to exclude dead cells. Furthermore, Cas9-GFP (FITC-A) negative and sgRNA library (Thy1.1-APC-A) negative cells were excluded. Cells were sorted into BRD4HIGH, BRD4MID and BRD4LOW fractions based on BRD4-TagBFP (BV421-A) vs. mCherry (PE-Texas Red-A) scatter plots. These gates were dynamically adjusted to keep the percentage to ~10% for BRD4HIGH and BRD4LOW and ~30% for BRD4MID populations.<br>A figure exemplifying the gating strategy for all FACS experiments and FACS-based screens in provided in the Supplementary information file of the manuscript.

- [x] Tick this box to confirm that a figure exemplifying the gating strategy is provided in the Supplementary Information.

Magnetic resonance imaging

Experimental design

Design type — *Indicate task or resting state; event-related or block design.*

Design specifications — *Specify the number of blocks, trials or experimental units per session and/or subject, and specify the length of each trial or block (if trials are blocked) and interval between trials.*

Behavioral performance measures — *State number and/or type of variables recorded (e.g. correct button press, response time) and what statistics were used to establish that the subjects were performing the task as expected (e.g. mean, range, and/or standard deviation across subjects).*

Acquisition

Imaging type(s) — *Specify: functional, structural, diffusion, perfusion.*

Field strength — *Specify in Tesla*

Sequence & imaging parameters — *Specify the pulse sequence type (gradient echo, spin echo, etc.), imaging type (EPI, spiral, etc.), field of view, matrix size, slice thickness, orientation and TE/TR/flip angle.*

Area of acquisition — *State whether a whole brain scan was used OR define the area of acquisition, describing how the region was determined.*

Diffusion MRI ☐ Used ☐ Not used

Preprocessing

Preprocessing software — *Provide detail on software version and revision number and on specific parameters (model/functions, brain extraction, segmentation, smoothing kernel size, etc.).*

Normalization — *If data were normalized/standardized, describe the approach(es): specify linear or non-linear and define image types used for transformation OR indicate that data were not normalized and explain rationale for lack of normalization.*

Normalization template — *Describe the template used for normalization/transformation, specifying subject space or group standardized space (e.g. original Talairach, MNI305, ICBM152) OR indicate that the data were not normalized.*

Noise and artifact removal — *Describe your procedure(s) for artifact and structured noise removal, specifying motion parameters, tissue signals and physiological signals (heart rate, respiration).*

Volume censoring — *Define your software and/or method and criteria for volume censoring, and state the extent of such censoring.*

Statistical modeling & inference

Model type and settings — *Specify type (mass univariate, multivariate, RSA, predictive, etc.) and describe essential details of the model at the first and second levels (e.g. fixed, random or mixed effects; drift or auto-correlation).*

Effect(s) tested — *Define precise effect in terms of the task or stimulus conditions instead of psychological concepts and indicate whether ANOVA or factorial designs were used.*

Specify type of analysis: ☐ Whole brain ☐ ROI-based ☐ Both

Statistic type for inference (See Eklund et al. 2016) — *Specify voxel-wise or cluster-wise and report all relevant parameters for cluster-wise methods.*

Correction — *Describe the type of correction and how it is obtained for multiple comparisons (e.g. FWE, FDR, permutation or Monte Carlo).*

Models & analysis

n/a	Involved in the study
☐	☐ Functional and/or effective connectivity
☐	☐ Graph analysis
☐	☐ Multivariate modeling or predictive analysis

Functional and/or effective connectivity	*Report the measures of dependence used and the model details (e.g. Pearson correlation, partial correlation, mutual information).*
Graph analysis	*Report the dependent variable and connectivity measure, specifying weighted graph or binarized graph, subject- or group-level, and the global and/or node summaries used (e.g. clustering coefficient, efficiency, etc.).*
Multivariate modeling and predictive analysis	*Specify independent variables, features extraction and dimension reduction, model, training and evaluation metrics.*
